# Evolutionary dynamics and impacts of chromosome regions carrying *R*-gene clusters in rice

**DOI:** 10.1038/s41598-020-57729-w

**Published:** 2020-01-21

**Authors:** Hiroshi Mizuno, Satoshi Katagiri, Hiroyuki Kanamori, Yoshiyuki Mukai, Takuji Sasaki, Takashi Matsumoto, Jianzhong Wu

**Affiliations:** 1 Institute of Crop Science (NICS), National Agriculture and Food Research Organization, 1-2, Ohwashi, Tsukuba, Ibaraki 305-8634 Japan; 2 Tokyo University of Agriculture, 1-1-1 Sakuragaoka, Setagaya-ku, Tokyo 156-0054 Japan

**Keywords:** Genome evolution, Comparative genomics, Molecular evolution, Genetic variation, Evolutionary genetics

## Abstract

To elucidate *R*-gene evolution, we compared the genomic compositions and structures of chromosome regions carrying *R*-gene clusters among cultivated and wild rice species. Map-based sequencing and gene annotation of orthologous genomic regions (1.2 to 1.9 Mb) close to the terminal end of the long arm of rice chromosome 11 revealed *R*-gene clusters within six cultivated and ancestral wild rice accessions. NBS-LRR *R*-genes were much more abundant in Asian cultivated rice (*O*. *sativa* L.) than in its ancestors, indicating that homologs of functional genes involved in the same pathway likely increase in number because of tandem duplication of chromosomal segments and were selected during cultivation. Phylogenetic analysis using amino acid sequences indicated that homologs of paired *Pikm1–Pikm2* (NBS-LRR) genes conferring rice-blast resistance were likely conserved among all cultivated and wild rice species we examined, and the homolog of *Xa3*/*Xa26* (LRR-RLK) conferring bacterial blight resistance was lacking only in Kasalath.

## Introduction

Resistance genes (*R*-genes) confer disease resistance on plants, often forming clusters and showing frequent changes in copy number among genomes^1–3^. As the sequences of cluster members are highly homologous, it is believable that the individual genes have evolved possibly through duplication events^4,5^. Considerably, clustering of similar *R*-genes with the highly conserved sequences may lead to the creation of new resistance specificities via unequal crossing-over, gene conversion, or both which can be an important genetic resource^4,6^. Models including the birth-and-death model and the balancing model are often used to explain the evolutionary process of *R*-genes. Based on a prediction that defeated *R*-genes get lost rapidly from the host population due to a metabolic cost associated with the gene maintenance, the birth-and-death model proposes that new disease-resistance genes are born by gene duplication^4,7^. In plants, several *R*-genes such as wheat *Pm3*, *Arabidopsis thaliana RPP13*, flax *L* and capsicum *eIF4E*, for example, seem to have evolved in this way since they are likely involved in co-evolutionary relationships with pathogens^8–11^. The balancing model suggests that genetic variation in disease resistance is maintained, even though there exists a fitness cost associated with the preservation of temporarily non-functional *R*-genes^12^. *R*-genes of *RPM1*, *RPS2* and *RPS5* in Arabidopsis and *Lr21* in wheat have been studied, revealing that both functional and non-functional alleles show the coexistence over a long period of evolutionary time in wild populations^12–15^. Factors that explain the differences about the evolutionary patterns of *R*-genes in these two distinct models are still not well known.

Cultivated rice varieties are either Asian or African in origin. The Asian cultivated rice *Oryza sativa* L. consists of two main subspecies, *indica* and *japonica*, each of which has a wild rice as its ancestor. The wild rice that was the source of *O*. *sativa* ssp. *japonica* is *O*. *rufipogon* (a perennial wild rice); the one that was the source of *O*. *sativa* ssp. *indica* is *O*. *nivara* (an annual wild rice, also called annual-growth-type *O*. *rufipogon*); and the one that was the source of the African cultivated rice *O*. *glaberrima* is *O*. *barthii*^16–19^. It remains controversial whether or not *japonica* and *indica* were domesticated independently^20,21^.

Gene annotation from the whole genomic sequence of the *japonica* rice cultivar Nipponbare has revealed the existence of more than 500 *R*-gene models along its 12 chromosomes, encoding nucleotide binding site (NBS) – leucine-rich repeat (LRR) domains^22,23^. In particular, rice chromosome 11 is highly enriched in *R*-genes; up to 201 loci encode the domains of not only NBS-LRR but also LRR – receptor-like kinase (LRR-RLK) or wall-associated serine/threonine protein kinase (WAK), mostly in clusters^24^.

Breeding for a wide range of disease-resistance qualities is one of the major goals of rice improvement. To achieve this goal, various alleles of *R*-genes have been discovered and put to practical use. The rice gene *Xa3*/*Xa26*, identified on the long arm of chromosome 11 (Chr11L) in *indica* rice, encodes an LRR-RLK conferring resistance to *Xanthomonas oryzae*, which causes bacterial blight disease^25,26^. Another major blast-resistance locus located on Chr11L is *Pik*, derived from the *japonica* rice Tsuyuake. The blast resistance provided by one of the *Pik* alleles, *Pikm*, is conferred not by a single gene, but rather by a combination of two adjacent NBS-LRR genes (*Pikm1-TS*, *Pikm2-TS*)^27^. Although a few *R*-genes have been found to confer disease resistance, the functions of most of the clustered *R*-genes are still unknown.

To clarify how *R*-genes arise and evolve, it is desirable to compare gene duplication among plant accessions exposed and not exposed to disease for a long time. A comprehensive comparison of the sequences and structures of *R*-gene cluster regions between domesticated rice species and their wild ancestors would be an optimal way to give insights into the molecular evolution of *R*-genes of plants grown in different areas for a long time in cultivated fields or in wild environments. Current whole-genome resequencing with advanced next-generation sequencing (NGS) technology has promoted large-scale and genome-wide comparative genomics among different rice cultivars and wild rice accessions^18^. However, simply mapping the NGS short-read sequences onto the reference genome created from the *japonica* rice Nipponbare^23^ will likely not be effective for uncovering the structural complexity of *R*-gene cluster regions because of the rapid and dynamic changes in genomic composition and structure, caused particularly by chromosomal rearrangement events such as duplication and insertion or deletion of DNA segments during the evolution of rice species and even cultivated accessions. Therefore, to uncover the dynamics and evolution of *R*-gene cluster formation we need to obtain all of the relevant genomic information by completely sequencing these kinds of special regions independently in both cultivated and wild rice species.

Here, by map-based sequencing, we performed a comprehensive comparison among different cultivars and their ancestral wild accessions of the genomic composition and structure of a genomic region that is known to contain *R*-gene clusters and is located close to the terminal end of rice Chr11L. In the *japonica* rice cultivar Nipponbare, this chromosomal region, flanked by the two DNA markers R0251 and E50301, carries about 1.35 Mb of genomic sequences and at least 38 genes annotated to hold NBS-LRR or LRR-RLK domains^24,28^. Known as the largest *R*-gene cluster detected in the rice genome, this region includes important loci of the disease resistance genes *Pikm1* and *Pikm2* as well as *Xa3*/*Xa26*^26,27,29^.

## Results

### Overall comparison of chromosomal composition and structure

**Figure 1:**
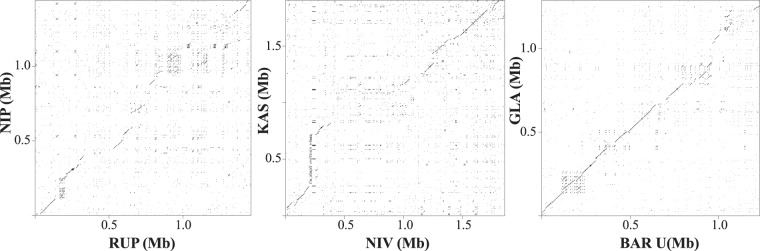
Comparative analysis of genomic sequences among *R*-gene cluster regions between cultivated and wild rice species. Sequence alignment between the Asian *japonica* rice variety Nipponbare (NIP) and its ancestor wild rice species *O*. *rufipogon* (RUP), the Asian *indica* rice variety Kasalath (KAS) and its ancestor wild rice species *O*. *nivara* (NIV) as well as the Africa cultivated rice *O*. *glaberrima* (GLA) and its ancestor wild rice species *O*. *barthii* (BAR) is shown respectively from the left to right. The position of matched sequences detected by BLASTZ is dot-plotted.

**Figure 2:**
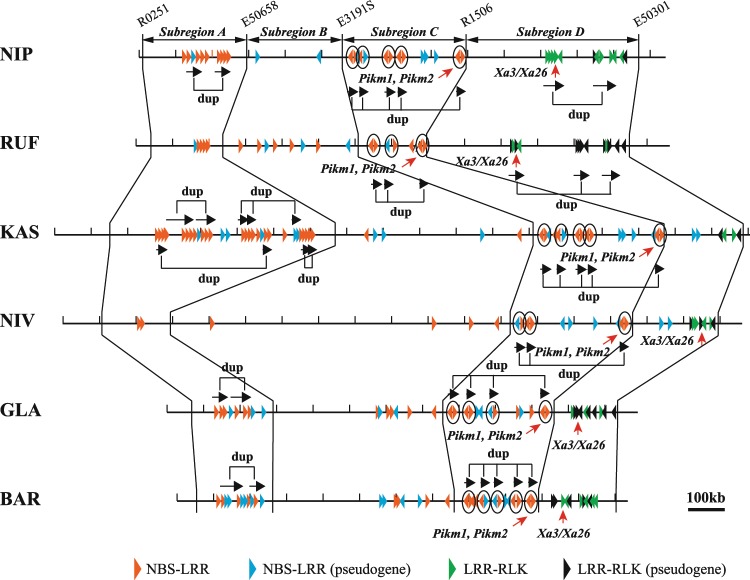
Detection of location and distribution pattern of *R*-genes. On the basis of the positions of DNA markers, the whole *R*-gene cluster region examined is divided into four subregions, A to D. Arrowheads indicate intact or pseudo NBS-LRR and LRR-RLK genes. Subregion C contains the *Pik*/*Pikm* locus responsible for rice blast resistance and subregion D contains the *Xa3*/*Xa26* locus responsible for bacterial blight disease resistance indicated by red arrows. The black arrows grouped by “dup” indicates the unit of segmental duplication. The duplicated gene pairs in opposite directions were circled.

By screening BAC libraries using nine DNA markers as well as BAC-end sequences, we constructed BAC-based chromosomal physical maps that completely covered the *R*-gene cluster region on each accession, with the sole exception of *O*. *rufipogon* W1943 (RUF), which had one gap remaining (Supplementary Fig. S1). Complete sequencing of the minimum-tiling-path BAC clones from each accession finally yielded non-redundant DNA sequences ranging from 1.23 to 1.91 Mb in length (Table 1, Supplementary Table S1). Sequence comparison by BLAST analysis proved the sequence conservation and chromosomal synteny of the above genomic region among cultivated and wild species, although substantial structural variations (as represented by large-scale insertions and deletions) were present, causing differences in the region’s physical length (Fig. 1, Supplementary Table S2). The Asian cultivated rice *O*. *sativa* ssp. *indica* Kasalath (KAS) and its wild ancestor *O*. *nivara* W0106 (NIV) had the most abundant DNA sequences on the orthologous region flanked by the two conserved DNA markers R0251 and E50301, amounting to 1.74 Mb and 1.69 Mb, respectively, of DNA (Fig. 2, Table 1). The Asian cultivated rice *O*. *sativa* ssp. *japonica* Nipponbare (NIP) and its wild ancestor RUF had 1.35 and 1.32 Mb, respectively—less than those of KAS and NIV. The African cultivated rice *O*. *glaberrima* IRGC104038 (GLA) and its wild ancestor *O*. *barthii* W1588 (BAR) had the smallest sequences, both with 1.17 Mb—smaller than in the Asian cultivated and wild accessions. Very clearly, the two Asian rice cultivars and their wild ancestors—particularly the annual-growth-type wild rice NIV—had a higher (34.3% to 50.1%) repeat content than the African cultivated rice GLA and its wild ancestor BAR (30.9% and 30.7%, respectively). The high content of repetitive sequences detected in the Asian rice species was probably due to the frequent amplification of retrotransposable elements in them (Supplementary Table S3). Annotation of transcripts including non-coding genes and pseudogenes within the above genomic sequences resulted in a total of 62 to 97 gene models (Table 1, Supplementary Table S4). At least 40.3% of the total predicted genes belonged to the *R*-gene family, because they encoded domains highly similar to those of the NBS-LRR and LRR-RLK gene sequences in public databases. KAS had many more *R*-gene loci than any of the other cultivated or wild rice accessions had, including 53 NBS-LRR genes and 4 LRR-RLK genes, which accounted for 58.8% of its total number of genes. The total numbers of *R*-genes were increased in both NIP and KAS but were slightly decreased in GLA relative to the numbers in their respective wild ancestors.

**Table 1:** Summary of map-based sequences and gene annotation determined from the *R*-gene cluster region on rice chromosome 11L.

Name	*Oryza sativa* Nipponbare	*Oryza rufipogon* W1943	*Oryza sativa* Kasalath	*Oryza nivara* W0106	*Oryza glaberrima* IRGC104038	*Oryza barthii* W1588
Abbreviation	NIP	RUF	KAS	NIV	GLA	BAR
Region	Asia	Asia	Asia	Asia	Africa	Africa
Cultivated or wild	cultivated	wild	cultivated	wild	cultivated	wild
Total length of non-redundant DNA sequences (bp)^a^	1,354,172	1,320,785^b^	1,740,657	1,690,167	1,172,253	1,165,008
GC content (%)	43.0	43.8	43.2	44.4	42.6	42.8
Repeat content (%)	34.3	39.4	38.2	50.1	30.9	30.7
Total number of annotated genes	84	79	97	62	82	95
Number of NBS-LRR *R*-genes^c^	26 (8)	20 (5)	53 (17)	18 (7)	29 (9)	33 (14)
Number of LRR-RLK *R*-genes^c^	12 (2)	13 (10)	4 (2)	7 (3)	10 (7)	11 (5)

^a^Calculated from the orthologous chromosomal region flanked by the two conserved DNA markers R0251 and E50301 (see Fig. 2) in all cultivated and wild rice accessions. ^b^Including 200 “Ns” used to represent the single physical gap remaining unclosed in *O*. *rufipogon*. ^c^Arabic numbers in blankets indicate pseudogenes.

### Chromosomal organization of *R*-genes

To describe and compare the constitutions and patterns of organization of all *R*-gene loci, for convenience we divided the whole genomic region into four subregions, A to D, on the basis of the conserved marker positions (Fig. 2, Supplementary Table S4). Within subregion A (between R0251 and E50658) we found 10 *R*-genes encoding the NBS-LRR domain of NIP (282 kb), but only five in its wild ancestor RUF (198 kb). We found up to 30 NBS-LRR family genes in KAS (618 kb), but only two in its wild ancestor NIV (183 kb). Thus, there were many more NBS-LRR genes in the Asian cultivated rices, particularly KAS, than in their ancestors. We found 9 NBS-LRR-family genes in the African GLA (219 kb) and 11 in its wild ancestor BAR (219 kb), indicating comparatively high conservation of *R*-gene-family numbers between the two.

Within subregion B (between E50658 and E3191S) we found only two NBS-LRR-family genes in NIP (250 kb), but eight within the same subregion of RUF (366 kb) (Fig. 2). KAS (542 kb) had five and NIV (926 kb) had four, whereas GLA (462 kb) had seven and BAR (494 kb) had eight, reflecting the lack of substantial change in copy number of *R*-genes between each cultivated rice and its wild ancestor. The big change in physical length between KAS and NIV was due to the marked difference in the amount of repetitive sequences abundant in long-terminal-repeat retrotransposons (Table 1, Supplementary Table S3).

Subregion C (between E3191S and R1506) corresponded to the region in rice that contains the *Pik/Pikm* locus, which confers blast resistance. We found 14 NBS-LRR-family genes in NIP (340 kb) and seven in RUP (181 kb), and 16 in KAS (348 kb) and 10 in NIV (335 kb), indicating that there was a marked increment in *R*-gene copy numbers in the Asian cultivated rices relative to their ancestors, as in subregion A. *Pikm*-specific resistance is conferred by the cooperation of the *Pimk1-TS* and *Pikm2-TS* genes, which are adjacent to each other and are transcribed in opposite directions^27^. Very interestingly, many adjacent NBS-LRR gene pairs with opposite transcription directions were found within this region, including five pairs in NIP, three in RUF, five in KAS, three in NIV, four in GLA, and five in BAR (Fig. 2). On the other hand, there was almost no change in NBS-LRR gene numbers between the two African rice accessions, with 13 in GLA (309 kb) and 14 (221 kb) in BAR. No LRR-RLK genes were detected within subregion A, B or C.

Subregion D (between R1506 and E50301) carries the bacterial blight resistance gene locus *Xa3*/*Xa26*^26,29^. Only *R*-genes encoding the LRR-RLK domain were observed within subregion D of four of the accessions, at 12 in NIP (459 kb), 13 in RUF (554 kb), 10 in GLA (164 kb) and 11 in BAR (208 kb). In contrast, two NBS-LRR genes, together with four and seven LRR-RLK family genes, were detected respectively within the KAS (209 kb) and NIV (228 kb). There was thus a reduction in *R*-gene numbers in the *indica* rice relative to its ancestor.

### Phylogenetic tree of *R*-genes

**Figure 3:**
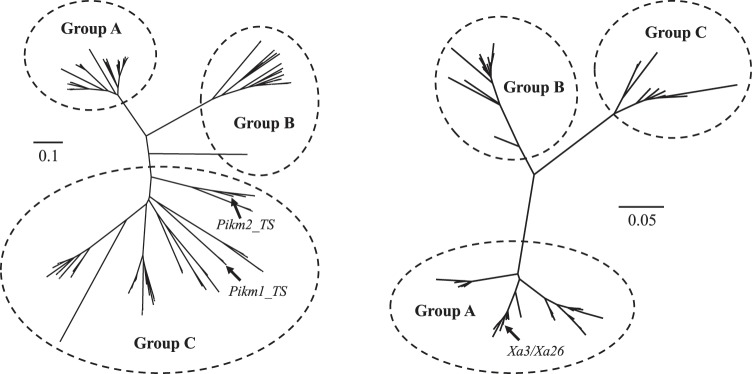
Phylogenetic analysis of *R*-genes. Phylogenetic trees are created by the neighbour-joining method from amino acid sequences derived from the domain region of NBS-LRR (left) and LRR-LRK (right) genes in all species analyzed. Arrows indicate locations of *R*-genes with known functions.

Phylogenetic analysis of all *R*-genes by using amino acid sequences from the NBS domain identified three major groups of NBS-LRR genes (Fig. 3 also see Supplementary Fig. S2 for details). Group A consisted of 67 NBS-LRR family genes, all derived from subregion A. Group B had 34 genes, all from subregion B. Group C had 78 genes, most from subregion C, and four (two in KAS and two in NIV) from subregion D. These data suggest that gene duplication was restricted to within each subregion. Clearly, the pair of *Pikm1-TS* and *Pikm-TS2*, both necessary for disease resistance, fell into Group C; their orthologs are conserved within the genomes of all of the cultivated and wild rice accessions: respectively RG025 and RG026 in NIP, RG050 and RG051 in KAS, RG019 and RG020 in RUP, RG015 and RG016 in NIV, RG028 and RG029 in GLA, and RG032 and RG033 in BAR. The phylogenetic tree revealed low sequence identity between the pairs of adjacent *R*-genes, clearly indicating that they are not paralogs generated directly by gene duplication but that they were instead duplicated in pairs.

Phylogenetic analysis of *R*-genes by using amino acid sequences from the kinase domains also yielded three major groups of LRR-RLK family genes, all within subregion D of the accessions (Fig. 3; also see Supplementary Fig. S3 for details). *Xa3*/*Xa26* was a member of Group A; its ortholog was likely conserved in NIP (RLK004), RUP (RLK003), NIV (RLK004), GLA (RLK003) and BAR (RLK003), but it seems to have been lost in KAS.

The sequence identities (phylogenetic tree) and chromosomal organization shown by the *R*-genes provide strong evidence for the evolutionary event of segmental duplication in all rice accessions we investigated. For example, at least five segmental duplication events seem to have happened so far within subregion A of KAS, unlike in its wild ancestor NIV (Fig. 2). Only one duplication of *R*-genes, however, likely occurred in the African cultivated GLA within the same subregion; it obviously had its origin in the wild ancestor BAR, indicating that the event took place before the divergence of the two closely related species. Within subregion C, carrying the paired genes *Pikm1* and *Pikm2* and their homologs, on the other hand, NIP and KAS both seem to have experienced gene duplications four times, two of which could be traced back to their wild rice ancestors, RUF and NIV (Fig. 2). Some of the duplications revealed species specificity: for example, the segmental duplications carrying LRR-RLK genes within subregion D were likely restricted to NIP and its ancestor RUP, because no strong evidence indicating segmental duplication with multiple *R*-genes was found in the same subregion of KAS, NIV, GLA or BAR.

## Discussion

### Domestication and subsequent repetitive cultivation by humans have increased the number of *R*-genes

**Figure 4:**
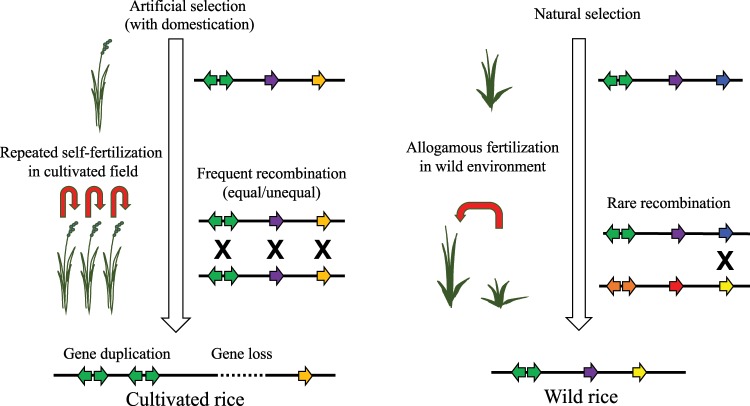
Differences in the evolution of *R*-genes between cultivated and wild rice species. Cultivated rice loses its heterozygosity during domestication as self-fertile individuals are cultivated repeatedly. As a result, duplication of *R*-genes tends to occur, and sometimes loss of *R*-genes happens in parallel. The characteristics of the birth-and-death model are therefore notable in cultivated rice. In contrast, wild rice maintains non-functional alleles. Allele introgression from other individuals occurs, causing the accumulation of mutations, thus potentially leading to the creation of functional alleles under natural conditions. The characteristics of the balancing model are therefore notable in wild rice.

The numbers of NBS-LRR family genes were substantially greater in the Asian cultivated rices than in their wild ancestors (Table 1). This difference is probably due to the effects of domestication and of repeated cultivation on genotype (cultivar vs. wild: homozygous or heterozygous), fertilisation process (self-fertilising vs. allogamous), plant population density (dense vs. sparse), and generation time (annual single or multiple harvests vs. perennial or annual). In cultivated rice, it is desirable for alleles to be homozygous and for plants to be self-fertilising so as to maintain the phenotype. The copy number of tandem *R*-genes increases or decreases with chromosomal recombination, but the rate of these changes will increase if genetically homozygous, self-fertilising individuals are raised repeatedly in densely planted fields. In addition, the life cycle of cultivated rice is annual even with multiple cropping, whereas that of wild rice is either annual or perennial. Cultivated rice has been artificially selected for adaptation to each cultivation area. We consider that these factors have promoted the duplication of *R*-genes in cultivated rice: the ready potential of *R*-gene clusters to multiply has been promoted markedly by domestication and repeated cultivation (Fig. 4).

The region including the NBS-LRR genes showed comparatively high sequence identity and similar *R*-gene numbers in the African cultivated rice GLA and its wild ancestor BAR, indicating an extremely high level of conservation of chromosomal synteny (Fig. 1, Table 1). In Africa, *O*. *glaberrima* was domesticated from *O*. *barthii* about 3000 years ago, much later than domestication of *O*. *sativa* approximately 9000 years ago^30^. In future, it will be important to perform a comprehensive analysis and comparison of the population structures of the African and Asian rice Accessions to determine whether the above differences in evolutionary and domestication history have had a marked influence on the changes in *R*-gene copy numbers in rice genomes.

### Features of the birth-and-death model predominate in *R*-genes of Asian cultivated rice

The major mechanism of *R*-gene birth was tandem duplication, which we found frequently in Asian cultivated rice (Fig. 2). Respectively, 26.3% and 33.3% of all *R*-genes in NIP and KAS were pseudogenes (Table 1). This phenomenon therefore represents a birth-and-death model that occurred in a relatively short period of time after rice was domesticated. To have duplicated genes is costly, even if the genes are intact. However, because in the cultivated environment there is little or no competition for survival against neighbouring plants, this disadvantage may not be an issue.

### Features of the balancing model predominate in *R*-genes of wild rice

Variations in functional alleles were conserved intact. Within subregion C, the paired resistance genes *Pikm1* and *Pikm2* were conserved intact across all six accessions, and pseudogenes were observed from their paired homologs (Fig. 2). This finding suggests that the paralogs remain intact, and non-functional alleles are retained over relatively long periods of time. Such a situation is consistent with the balancing model. This model proposes that variation in disease resistance is maintained, even though there is a fitness cost associated with the maintenance of *R*-genes in the absence of their matching pathogens. Examples of the acquisition of resistance after the start of cultivation are known: at least five alleles—*Pik*, *Pikm*, *Pikh*, *Pikp* and *Piks—*have been identified on chromosome 11 as blast resistance genes^31–33^. *Pik* and *Pikm* appear to have emerged after the domestication of rice, whereas *Pikp* emerged before^34^. Many intact but non-functional alleles—especially in wild rice—have the potential to change through mutation to functional alleles conferring disease resistance.

Large deletion carries a risk of irreversible change and the loss of a specific gene family. Deletion of a region and changes to a pseudogene are both irreversible changes that can lead to the loss of an *R*-gene. In KAS, the gene corresponding to *Xa3/Xa26* was lost, probably owing to a deletion within subregion D (Fig. 2). Irreversible loss of an allele of a disease-resistance gene is contrary to the balancing model. KAS is a drought tolerant upland landrace traditionally grown in the short summer in Bangladesh under rainfed conditions. We consider, therefore, that the loss of *R*-genes may not be critical in some cultivated rice plants that are grown under environmental conditions that lack the pressures that might alter their disease resistance to a given pathogen.

### Differences between amplifiable and difficult-to-amplify *R*-genes

Duplication of the NBS-LRR gene was restricted within each subregion (Fig. 2). Some subregions showed greater sequence identity between orthologs than between paralogs and maintained chromosomal synteny. Other subregions showed copy number variation and disruption of chromosomal synteny. Why do contiguous *R*-genes have such different evolutionary patterns?

Within subregion A, the substantial amplification of *R*-gene copies in Asian cultivated rice—particularly in KAS—compared with their wild ancestors could be easily regarded as being due to multiple periods of segmental duplication. A similar case was present within subregion C: each duplication event was clearly associated with two paired genes showing opposite transcription directions, either orthologous or paralogous to the paired genes *Pik1* and *Pik2* of known function^27^. The *RPP2* locus in Arabidopsis is another example in which two NBS-LRR family genes, *RPP2A* and *RPP2B*, have developed to work with disease resistance by pairing, thus cooperating to provide target recognition or signalling functions lacked by either partner protein^35^. Our results thus demonstrate that neighbouring genes that provide disease resistance cooperatively were often duplicated together. Unlike in subregions A and C, no marked changes in *R*-gene copies have occurred within subregions B and D between the species of cultivated and wild rice. The number of *R*-genes present within subregion B in NIP has even dropped to a quarter of that in its wild ancestor RUP.

In conclusion, the *R*-gene-cluster region located close to the terminal end of the long arm of rice chromosome 11 harbours genomic sequences on a megabase scale; this region carries subregions that clearly show differences in *R*-gene-amplification ability. We believe that subregions A and C hold chromosomal sites with high frequency of unequal recombination. These were probably induced by retrotransposon activity that promoted segmental duplications carrying *R*-genes that were selectively retained by humans during the long period of domestication and regional adaptation of rice.

### The functions of most of *R*-genes are unknown

Exploration and use of *R*-genes are among the most important goals of crop breeding. Although there are many genes in the *R*-gene-cluster regions predicted to carry NBS-LRR or LRR-RLK domains, the real functions of most of them are unknown. An important future task will be to elucidate whether these *R*-gene paralogs have no function or whether simply their target fungi have not yet been identified. Genes that already endow resistance to specific pathogens are conserved, but highly mutated genes represent a potential reserve against future pathogens. This concept is consistent with the observation that clustered *RGC2* genes in lettuce can exhibit heterogeneous rates of evolution^36^. It is not known why only specific genes among an abundance of paralogs are responsible for disease resistance. *Pik* and *Pikm* are the same gene^27^. *Xa3* and *Xa26* are also the same gene^29^. In the case of *Cf-9* in tomato, sequence exchange occurs more frequently between orthologs than between paralogs^37^; this is also true of the *Pi2/9* locus in rice^38^ and Arabidopsis^4^. This process would be expected to conserve the sequence identity and function of alleles, and thereby to promote the rapid development of new pathogen-recognition specificities under selection. This might be particularly important for cultivars when they are moved to new planting areas to face different environmental conditions.

It is very important to consider the chromosomal rearrangement events of single- or multiple-gene duplication, indels and sequence mutations that could lead to changes in gene number and to pseudogenisation among different genomes. Comprehensive comparison on a large scale in an effort to understand in detail the contents and evolution of *R*-gene clusters in rice is not possible simply by mapping short reads onto one reference genome. Currently, whole genome reference sequences have been assembled from several rice varieties through map-based sequencing and/or whole-genome sequencing with the NGS technology^39,40^. Comparing the sequence data located within the orthologous region of *R*-gene cluster between the two Chinese elite *indica* rice varieties, Minghui 63 and Shuhui498, and the six cultivated and wild rice species reported here by BLAST analysis confirmed the presence of dramatic genome divergence between or even within a single *Oryza* species, providing furthermore the genomic variations for future understanding of the evolutionary mechanisms on *R*-genes (Supplementary Figs. S4 and S5). The genomic sequences obtained at the present study thereafter can be used as a platform for further comparative studies of *R*-gene clusters within cultivated and wild rice populations and will certainly help to elucidate those nucleotide substitutions that are critical to disease resistance. To our knowledge, this study is the first to systematically obtain referenced genomic sequences from the large *R*-gene cluster in both cultivated and wild rice species. These findings should have a substantial impact on our understanding of the evolution and function of disease-resistance genes in plants.

## Methods

### Construction of BAC-based physical map

Besides the *japonica* rice cultivar Nipponbare (NIP; acc. no. WRC001, Japan), the whole genomic sequence of which is publicly available, we used the *indica* rice cultivar Kasalath (KAS; acc. no. WRC002, Bangladesh), two Asian wild rice accessions, *O*. *rufipogon* (RUF; acc. no. W1943, China; ancestor of *japonica*) and *O*. *nivara* (NIV; accession no. W0106, India; ancestor of *indica*), one accession of African cultivated rice *O*. *glaberrima* (GLA; accession no. IRGC104038, Senegal), and one of African wild rice *O*. *barthii* (BAR; accession no. W1588, Cameroon). The close genetic relationships of the cultivated rices with their wild ancestors have been confirmed both at the gene level^41–43^ and at a population structure level, as analysed by using genome-wide retrotransposon markers (in preparation). BAC libraries of each cultivar or accession were constructed as described before^41,44^. Positive BAC clones respectively covering the targeted *R*-gene cluster region on Chr11L of KAS, GLA, RUF, NIV and BAR orthologous to NIP Chr11L were screened by using a number of genetic or EST markers, or both, with a polymerase-chain reaction (PCR) method similar to that reported previously^45,46^. End sequences of positive BAC clones were also used for chromosomal walking with the same PCR method to select new BAC clones covering any of the physical gaps remaining within the above genomic region.

### BAC sequencing and gene annotation

Minimum-tiling-path BAC clones were chosen from the BAC physical maps and sequenced by using a shotgun sequencing approach described before^23^. Genes were predicted by using an annotation system we developed previously^23,47^. Genes predicted to encode a hypothetical protein or transposable element were ignored. Pseudogenes were defined when coding sequences identical to functional genes were truncated because of the presence of disruptive mutations such as frame shifts or premature stop codons. Total amounts of repetitive sequences were calculated in RepeatMasker v. 1.332 software (http://www.repeatmasker.org) with the *Oryza* Repeat Database (http://rice.plantbiology.msu.edu/annotation_oryza.shtml) as a reference.

### Sequences and structural comparison

Genomic sequences on the *R*-gene cluster region were compared between each cultivated and wild rice pair by BLAST algorithm^48^. BLAST alignments were dot-plotted in Dottup software (http://www.bioinformatics.nl/cgi-bin/emboss/help/dottup) to explore the conservation of chromosomal composition and structure between cultivated and wild rice accessions. To analyse the development and evolution of *R*-genes, we performed a phylogenetic analysis using amino acid sequences spanning the region from the P-loop to the GLPL motif of NBS-LRR genes and the whole kinase domain of LRR-RLK genes, including those present within pseudogenes. Sequences were aligned in MAFFT software^49^ to generate neighbour-joining trees in MEGA7 software^50^.

## Supplementary information

Supplementary information.

Dataset 1.

Dataset 2.

Dataset 3.

Dataset 4.

Dataset 5.

Dataset 6.

Dataset 7.

## Data Availability

Genomic sequences of each BAC clone are available at NCBI (see Supplementary Table S1 for the accession number). Pseudomolecule sequences created from the above BAC sequences for each cultivated rice and wild rice species, and amino acid sequences of each predicted *R*-gene are also included within the supplementary Data on line.
