# Supplementary material for: Evolutionary dynamics and impacts of chromosome regions carrying R-gene clusters in rice
Source: Sci Rep. 2020 Jan 21;10:872. doi: 10.1038/s41598-020-57729-w (PMC6972905; doi:10.1038/s41598-020-57729-w)
Supplement: Supplementary file 1 — Supplementary information. [file 41598_2020_57729_MOESM1_ESM.docx]

**Evolutionary dynamics and impacts of chromosome regions carrying *R*-gene clusters in rice**

Hiroshi Mizuno^1^, Satoshi Katagiri^1^, Hiroyuki Kanamori^1^, Yoshiyuki Mukai^1^, Takuji Sasaki^2^, Takashi Matsumoto^2^ and Jianzhong Wu^1^*

^1^ Institute of Crop Science (NICS), National Agriculture and Food Research Organization, 1-2, Ohwashi, Tsukuba, Ibaraki 305-8634, Japan

^2^ Tokyo University of Agriculture, 1-1-1 Sakuragaoka, Setagaya-ku, Tokyo 156-0054, Japan

*e-mail: [jzwu@affrc.go.jp](mailto:jzwu@affrc.go.jp)
