# Supplementary material for: Evolutionary dynamics and impacts of chromosome regions carrying R-gene clusters in rice
Source: Sci Rep. 2020 Jan 21;10:872. doi: 10.1038/s41598-020-57729-w (PMC6972905; doi:10.1038/s41598-020-57729-w)
Supplement: Supplementary file 8 — Dataset 7. [file 41598_2020_57729_MOESM8_ESM.docx]

**Evolutionary dynamics and impacts of chromosome regions carrying *R*-gene clusters in rice**

Hiroshi Mizuno1, Satoshi Katagiri1, Hiroyuki Kanamori1, Yoshiyuki Mukai1, Takuji Sasaki2, Takashi Matsumoto2 and Jianzhong Wu1*

1 Institute of Crop Science (NICS), National Agriculture and Food Research Organization, 1-2, Ohwashi, Tsukuba, Ibaraki 305-8634, Japan

2 Tokyo University of Agriculture, 1-1-1 Sakuragaoka, Setagaya-ku, Tokyo 156-0054, Japan

*e-mail: jzwu@affrc.go.jp

>NIP_RG001

VQKWLDQLRDVMYDVDDIIDLARFKGSVLLPNYPMSSSRKSTACSGLSLSSCFSNIRIRH

EVAVKIRSLNKKIDNISKDDVFLKLSLTQHNGSGSAWTPIESSSLVEPNLVGKEVVHACR

EVVDLVLAHKAKNVYKLAIVGTGGVGKTTLAQKIFNDKKLEGRFDHRAWVCVSKEYSMVS

LLAQVLSNMKIHYEKNESVGNLQSKLKAGIADKSFFLVLDDVWHYKAWEDLLRTPLNAAA

TGIILVTTRDETIARVIGVDRTHRVDLMSADIGWELLWRSMNIKEEKQVKNLRDTGIEIV

RKCGGLPLAIRAIAKVLASLQDQTENEWRQILGKNAWSMSKLPDELNGALYLSYEVLPHQ

LKQCFLYCALFPEDATIFCGDLTRMWVAEGFIDEQEGQLLEDTAERYYHELIHRNLLQPD

GLYFDHSRCKMHDLLRQLASYLSREECFVGDPESLGTNTMCKVRRISVVTEKDIVVLPSM

DKDQYKVRCFTNFSGKSARIDNSLFKRLVCLRILDLSDSLVHDIPGAIGNLIYLRLLDLD

RTNICSLPEAIGSLQSLQILNLQGCESLRRLPLATTQLCNLRRLGLAGTPINQVPKGIGR

LKFLNDLEGFPIGGGNDNTKIQDGWNLEELGHLSQLRCLDMIKLERATPCSSTDPFLLSE

KKHLKVLNLHCTEQTDEAYSEEGISNVEKIFEKLEPPHNLEDLVIGDFFGRRFPTWLGST

HLSSVKYVLLIDCKSCVHLPPIGQLPNLKYLKINGASAITKIGPEFVGCWEGNLRSTEAV

AFPKLEWLVIKDMPKWEEWSFVEEEEVQEEAAAAAKEGGEDGIAASKQKGEEAPSPTPRS

SWLLPCLTKLDLVGCPKLRALPPQLGQQATNLKKLFIRDTRYLKTVEDLPFLSGGLQVEG

CEGLERVSNLPQVRELFVNECPNLRHVEELGGLEQLWLDEGMQEISSLWVPRLQEQHRQL

HGDEHELEVTEWL*

>NIP_RG002

MATILDSLVGSCANKLKEIITEEVILILGIQEELAELQRKTELIHCCISDGCDAEARRME

ESAVDNWLARHEVAVKIRSLNRKIENISKDRVFLTLKSTVPTGSSSVLRVRKSSHLLEPN

IVGKEIIHACRKMVDLVLEHKGRKLYKLAIVGTGGVGKTTLAQKIYNDRKIKGSFNKKAW

VCVSKVYSKASLLRELLRIMEVHHDQDESIGELQSKLEIAIKETSFFLVLDDMWQSDAWT

NLLRIPLHAAEMGAILITTRNNIVALEIGVDHTYRVDLMSTDVGWELLCKSMNISESIEL

QTLQDVGIEIVRKCGCLPLAIKVIARVLASKEQTENEWKKILSKNAWFMNNLPNDLRGAL

YLSYDELPRHLKQCFLYCSVYPEDANIYHDDLTRMWIAEGFIEDHGGQLLEETADEYYYE

LIHRNLLQPDGLYYDHSSCKMHDLLRQLACYLSREECFVGNPESLVGNTVSKLRRVSVVT

DKNMVMLPSMDEVQYKVRTWKTSYEKTLRVDNSFFKRFPYLRVLDLTDSFVPSIPGCIGN

LIHLRLLDLDGTNVSCLPESIGNLKNLQILNLERSVALHSLPSAITQLCNLRRLGLNYSP

IYQVPKGIGKLEFLNDVEGFPVYGGSSNTKMQDGWNLEELAYLYQLRRLHMIKLERAAYR

TTYPLLTDKGFLKFLYLWCTERTDEPYTEKDFSNIEKIFEQLIPPCNLEDLAIVKFFGRQ

YPFWIDSTHLAYVKSLHLFNCKFCMHLPPVGQLPNLKYLKIEGAAAVTIIGPEFAGHRAS

NLGRTVAFPKLEELLIRDMPNWEEWFFIDEATSTAKERVDDGDSAIPKEKALPPRMQILS

RLRRLELSGCPKLKALPQQLAQINSLKEIELRWASSLKVVENFPLLSEMLLIATCQALEK

VSNLPQVRELRVQDCPNLRLVEDLSTLEQLWLYEDMHEVSTLWVPGLQQQCRQHHGEDLE

VYNWT*

>NIP_RG004

MATIVDTLVGSCINKLQAIITDKTILILGVKDELEELQRRTNVIRSSLQDAEARRMEDSV

VEKWLDQLRDVMYDVDDIIDLARFKGSVLLPDYPMSSSRKATACSGLSLSSCFSNIRIRH

EVAVKIRSLNKKIDNISKDEVFLKLNRRHHNGSGSAWTPIESSSLVEPNLVGKEVIRACR

EVVDLVLAHKKKNVYKLAIVGTGGVGKTTLAQKIFNDKKLEGRFDHHAWACVSKEYSRDS

LLRQVLRNMGIRYEQDESVPELQRKIKSHIANKSFFLVLDDVWNSEAWTDLLSTPLHAAA

TGVILITTRDDTIARVIGVDHTHRVDLMSADVGWELLWRSMNINQEKQVQNLKDIGIEIV

RKCGGLPLAIRVIATVLASQEQTENEWRRILGKNAWSMSKLPRELSGALYLSYEVLPHQL

KQCFLYCALFPEDASILRDDLTRMWVAEGFIDEEKGQLLEDTAERYYYELIHRNLLQPDG

LYFDHSSCKMHDLLRQLASYLSREECFVGDPESLGTNTMCKVRRISVVTEKDIVVLPSMD

KDQYKVRCFTNLSGKSARIDNSLFERLVCLRILDLSDSLVHDIPGAIGNLIYLRLLDLDK

TNICSLPEAIGSLQSLQILNLQGCESLRRLPLATTQLCNLRRLGLAGTPINLVPKGIGRL

KFLNDLEGFPIGGGNDNTKIQDGWNLEELAHLSQLRQLGMIKLERATPCSSRDPFLLTEK

KHLKVLNLHCTEQTDEAYSEEGISNVEKIFEKLAPPHNLEVLAIVNFFGRRFPTWLGTNH

LSSVKYVLLIDCKSCVHLPPIGQLPNLKYLKINGASAITKIGPEFVGCWEGNLRSTEAVA

FPKLEWLVIEDMPNWEEWSFVEEEEVQEEEAAAAAKEGGEDGIAASKQKGEEAPSPTPRS

SWLLPCLTKLDLVGCPKLRALPPQLGQQATNLKDLLIREAECLKTVEDLPFLSGALSIGG

CEGLERVSNLPQVRELFLNVCPNLRHVEELGGLEQLLLDEGMQEISQLWVPRLQEQHRQL

HGDEHELEVIEWL*

>NIP_RG005

MATILDSLIGSCAKKLQEIITEEAILILGVKEDLRELQEKMEQIRCFISDVERRGMEDSS

IHNWISRLKDAMYDADDIIDLVSFEGSKLLNGHSCSPRKTIACNGLSLLSCFSNIRVHHE

IGNKIRSLNRKLEEIAKDKIFVTLENTQSSHKDSTSELRKSSQIAESNLVGKEILHASRK

LVSQVLTHKEKKTYKLAIIGTGGIGKTTLAQKVFNDEKLKQSFDKHAWICVSQDYSPASV

LGQLLRTIDAQCKQEESVGELQSKLESAIKDKSYFLVLDDVWQSDVWTNLLRTPLYAATS

GIVLITTRQDTVAREIGVEEPHHIDQMSPAVGWELLWKSINIEDEKEVQNLRDIGIEIVQ

KCGGLPLAIKVIARVLASKDKAENEWKKILANYVWSMYKLPKEIRGALYLSYDDLPQHLK

QCFLYCIVYPEDWTIHRDYLIRLWVAEGFVEVHKDQLLEDTAEEYYYELISRNLLQPVDT

SFDQSKCKMHDLLRQLACHLSREECYIGDPTSLVDNNMCKLRRILAITEKDMVVIPSMGK

EEIKLRTFRTQPNPLGIEKTFFMRFTYLRVLDLTDLLVEEIPDCVGYLIHLRLLDLSGTN

ISCLPKSIGALKNLQMLHLQRCESLYSLPSMITRLCNLRRLGLDDSPINQVPRGIGRLEF

LNDLEGFPVGGGSDNTKMQDGWNLQELAHLSQLRRLDLNKLERATPRSSTDALLLTDKKH

LKSLHLCCTEPTDEACSEEGISNVEMIFEQLSPPRNLEDLMIVLFFGRRLQILCASSTNW

TATNLKYLRIDGASAITKIGPEFVGCWEGNLISTETVAFPRLELLAIKDMPNWEEWSFVK

EEELQEEKAAAAAQEGGKDGTAASKQKGEEAPSPTPRSSWLLPCLKQLQLVECPKLRALP

PQLGQQATNLKELDIRRARCLKMVEHLPFLSGILFVQSCQGLEIISNLPQVRELLVNHCP

NLRHVEMLGGLEQLWLSKNMQKISSLWVPGLEEQHRQLHGDEHKLERCRRAHEKKVEVKV

DPIGRTAAGRRSKGDGIRNRPRIVAAAVVLASATGVVAAAGVIAAAVGVIIAVTGVVSAP

LQGPWLQWISRTPSAAWSQESQR*

>NIP_RG006

MATILGSLIGSCVNKLQGIITEEAILILGVEEELKKLQKRMKQIQCFLSDAERRGMEDSA

VHNWVSWLKDAMYDADDIIDLASFEGSKLLNGHSSSPRKTTACGGLSPLSCFSNIQVRHE

IGDKIRSLNRKLAEIEKDKIFATLKNAQPADKGSTSELRKTSHIVEPNLVGKEILKVSRN

LVCHVLAHKEKKAYKLAIVGTGGIGKTTLAQKLFNDQKLKGSFNKHAWICVSQDYSPSSV

LRQLLRTMEVQHRQEESVGELQSKLELAIKDKSYFLVLDDVWQHDVWTNLLRTPLHAATS

GIILITTRQDIVAREIGVEKQHRVDQMSPADGWELLWKSISIQDEKEVQNLRDIGIKIIQ

KCGGLPLAIKVIARVLASKDKTENEWKRILDKNVWSMAKLPKEIRGALYLSYDDLPQHLK

QCFLYCIVFPEDWTIHRDYLIRMWVAEGFVEVHKDQLLEDTAEEYYYELISRNLLQPVNT

SFDKSQCKMHDLLRQLACYISREECYIGDPTSCVDNNMCKLRRILVITEKDMVVIPSMGK

EEIKLRTFRTQQHPVGIENTIFMRFMYLRVLDLSDLLVEKIPDCIGHLIHLHLLDLDRTC

ISCLPESIGALKNLQMLHLHRCKSLHSLPTAITQLYNLRRLDIVETPINQVPKGIGRLKF

LNDLEGFPVSGGSDNAKMQDGWNLEELADLSKLRRLIMINLERGTPHSGVDPFLLTEKKY

LKVLNLWCTEQTDEAYSEENASNVENIFEMLTPPHNLRDLVIGYFFGCRFPTWLGTTHLP

SVKSMILANCKSCVHLPPIGQLPNLNYLKIIGASAITKIGPEFVGCREGNLISTEAVAFP

KLEMLIIKDMPNWEEWSFVEQEEEEVQEEEAVAAAKEGGEDGTVASKQKGKVALSPRSSW

LMPCLRRLDLWDCPKLRALPPQLGQTNLKELLIRYTS*

>NIP_RG007

MAAVLDALIVSCIKKLQDIITDNAILVLGVEEELSELVRKTDYIRCSLNDAETTRLDHEA

VNNWLGQLRDVMYDVDDIIDLARFKGSILLADHPSSSSSKPTRCNCPSCHCNIWTRHEVA

VKIRSLNKKIANISNDELLQDLRRRPHPRNGSIWTPIKTSSIVEPNLVGEEVILACRELV

DLVIENKEKKDYKLGIVGTGGVGKTTLAQQIYNDEKITGNFDKHAWVCVSTDSTQTSLLE

EVLRIMKIRYGKAKSVEELQNKLKSAIKEKSFFLVLDDVWESNAWTNSLQKPLHAAAKGI

VIVTTRNEKVAQEIKVDHTHQVHLMSENVGWDLLWKSMGITEEKQVHHLRDIGIEIVHQC

GYLPLAIKIIAKVLISKEKTNDEWKRILSNNSWSMNNLPDELRALYLSYSELSHQLKQCF

LYCAIYPEHSTINRDDLTSMWVAEGFIDEQKDQLLEDTAVEYYNELIHWNLLQLDLSYFD

LGGCKMHGLLRQLVCYLSREECFVGDPESQNGNTMSKMRRVSVVTEKDTVVLPSMDKKQY

KVRTYRTSYSKSLRVYNPLFKRLTYLRVLDLTGTLVQSIPSHIGNLIHMRLINLDGTNIS

CLPESVGNLQNLQVLNLQRCKSLYRLPLATTKLCNLRRLGLLDTPINKVPKGIGRLQFLN

DLEGFPIGSVTYNKKMQDGWNLEDLADLSQLRRLVMLPNLERLKIVKATAITKIGPEFIG

QFPRSREAVAFPKLEWLIINDMPNWEEWAFIEEDEISLAAMNEGGGDGTAVAEEEEISLA

AMNEGGEDGTTVTEKGEASFPRLCVLPSLKRLDIDNCPKLRALPQQLGHEATSLKELSLV

AASCLKSVKDLPSLSGFLSVCRCEGLERVTELPKVRKLFVSVCPNLSCVEELGSLEQLWL

DVNMEDSSPWVHGLREQYRHLDVYTWPRE*

>NIP_RG008

MATILDSLVGSCANKLKEIITEEVILILGIQEELAELQRKTELIHCCISDAEERRMEESA

VDNWLGQLREVLYDVDDIIDLARFKGSILLTDHPSSSSRKSIACTGLSISTCFSNVQARH

EVAVKIRSLNRKIENISKDRVFLTLKSTVPTGSSSVLRVRKSSHLLEPNIVGKEIIHACR

KMVDLVLEHKGRKLYKLAIVGTGGVGKTTLAQKIYNDRKIKGSFNKKAWVCVSKVYSEAS

LLRELLRIMEVHHDQDESIGELQSKLEIAIKETSFFLELDDMWQSDAWTNLLRIPLHAAE

MGAILITTRNNIVALEIGVDHTYRVDLMSTDVGWELLWKSMNISESIELQTLQDVGIEIV

RKCGCLPLAIKVIARVLASKEQTENEWKKILSKNAWFMNNLPNDLRGALYLSYDELPHHL

KQCFLYCSVYPEDANIYRDDLTRMWIAEGFIEDHGGQLLEETADEYYYELIHRNLLQPDG

LYYDHSSCKMHDLLRQLACYLSREECFVGNPESLVGNTVSKLRRVSVVTDKNMVMLPSMD

EVQYKVRTWKTSYEKTLRVDNSFFKRFPYLRVVDWTDSFVPSIPGCIGNLIHLRLSTIGH

SDNVWMPKLFGRFGCLMQAHYQHINLLIEQESMNGSMGRWGMLISAPFGYFNLPNVPRLD

EYQSLYLNTYKVNSMVSVFQIRHSEGKGRHDTYLRVLQPQLQSQMEGVTCNRALHYAMNW

YMFTSQIEG*

>NIP_RG009

MATIVDTLVGSCINKLQAIITDKTILILGVKDELEELQRRTNVIRSSLQDAEARRMEDLV

VEKWLDQLRDVMYDVDDIIDLARFKGSVLLPDYPMSSSRKSTACSGLSLSSCFSNIRIRH

EVAVKIRSLNKKIDNISKDEVFLKLNRRHHNESGSAWTPIESSSLVEPNLVGKEVIRACR

EVVDLVLARKKKNVYKLAIVGTGGVGKTTLAQKIFNDKKLEGRFDHHAWACVSKEYSRDS

LLRQVLRNMGIRYEQDESVPELQRKIKSHIANKSFFLVLDDVWNSEAWTDLLSTPLHAAA

TGVILITTRDDTIARVIGVEHTHRVDLMSADVGWELLWRSMNINQEKQVQNLKDIGIEIV

RKCGGLPLAIRVIATVLASQEQTENEWRRILGKNAWSMSKLPRELSGALYLSYEVLPHQL

KQCFLYCALFPEDETILRDILTRMWVAEGFIDEEKGQLLEDTAERYYYELIHRNLLQPDG

LYFDHWSCKMHDLLRQLACYLSREECFVGDVESLGTNTMCKVRRISVVTEKDMMVLPSIN

KDQYKVRTYRTSYQKALQVDSSLFEKLTYLRVLDLTNSHVQRIPNYIENMIHLRLLDLDG

TDISHLPESIGSLQNLQILNLQRCKSLHRLPLATTQLCNLRRLGLAGTPINQVPKGIGRL

KFLNDLEGFPIGGGNDNTKIQDGWNLEELAYLPQLRQLGMIKLERGTPRSSTDPFLLTEK

KHLKVLNLDCTEQTDEAYSEENARNIEKIFEKLTPPHNLEDLFVGNFFGCRFPTWLGCTH

LSSVKSVILVDCKSCVHLPPIGQLPNLKYLRINGASAITKIGPEFVGCWEGNLRSTEAVA

FPKLEMLIFKEMPNWEEWSFVEEEEVQEEEAAAAAKEGGEDGIAASKQKGEEAPSPTPRS

SWLLPCLKQLQLVECPKLRALPPQLGQQATNLKKLFIRDTRYLKTVEDLPFLSGCLLVER

CEGLERISNLPQVRELRAGGCPNLRHVEELGGLEQLWLSKNMQKISSLWVPGLEEQHRQL

HGDEHKLEVNE*

>NIP_RG010

MATILDSLIGSCAKKLQEIITEEAILILGVKEDLRELQEKMEQIRCFISDVERRGMEDSS

IHNWISRLKDAMYDADDIIDLVSFEGSKLLNGHSCSPRKTIACNGLSLLSCFSNIRVHHE

IGNKIRSLNRNLEEIAKDKIFVTLENTQSSHKDSTSELRKSSQIAESNLVGKEILHASRK

LVSQVLTHKEKKTYKLAIIGTGGIGKTTLAQKVFNDEKLKQSFDKHAWICVSQDYSPASV

LGQLLRTIDAQCKQEESVGELQSKLESAIKDKSYFLVLDDVWQSDVWTNLLRTPLYAATS

GIVLITTRQDTVAREIGVEEPHHIDQMSPAVGWELLWKSINIEDEKEVQNLRDIVIEIVQ

KCGGLPLAIKVIARVLASKDKTENEWKKILANYVWSMDKLPKEIRGALYLSYDDLPQHLK

QCFLYCIVYPEDWTIHRYYLIRLWVAEGFVEVHKDQLLEDTAEEYYYELISRNLLQPVDT

SFDQSKCKMHDLLRQLACHLSREECYIGDPTSLVDNNMCKLRRILAITEKDMVVIPSMGK

EEIKLRTFRTQPNPLGIEKTFFMRFTYLRVLDLTDLLVEEIPDCVGYLIHLRLLDLSGTN

ISCLPKSIGALKNLQMLHLQRCESLYSLPSMITRLCNLRRLGLDDSPINQVPRGIGRLEF

LNDLEGFPVGGGSDNTKMQDGWNLQELAHLSQLRRLDLNKLERATPRSSTDALLLTYKKH

LKSLHLCCTEPTDEAYSEEGISNVEMIFEQLSPPRNLEDLMIVLFFGRRFPTWLSTSLLS

SLTYLKLKDCKSCVHLPPHNRTATNLKYLRIDGASAITKIGPEFVGCWEGNLISTETVAF

PRLELLAIKDMPNWEEWSFVKEEELQEEKAAAAAQEGGKDGTAASKQKGEEAPSPTPRSS

WLLPCLKQLQLVECPKLRALPPQLGQQATNLKELDIRRARCLKMVEHLPFLSGILFVQSC

QGLEIISNLPQVRELLVNHCPNLRHVEMLGGLEQLWLSKNMQKISSLWVPGLEEQHRQLH

GDEHKLEVNEWF*

>NIP_RG013

MEGSMFNLPGRLDRLLRRHGSMLPKGAEEEIPLIKQDLEEIISVLHGHCSKPKLEDHAMV

VRCWMKEVRELSYDIEDCIDQYEHAATATRSHTGPNICRRKFNQRHGKMIPWVPWKLKQR

LWMANKIREFSLRTQEALQRHTMYNNLGGITIASTTGGDVCSATPWHPTQFREHTDNVCS

VGIDADGMEAALNDLNKLKNLLASIPTASLVQFREHANKVRHIHPDVEAILNKLKNIPTG

ITTTSTTTRGDVSSTSSRQPTRFMESAGLVGINAAVNKLENLLDVCGEEKLKVVSIVGVG

GVGKTTLANKLYCKLQRQFECWAFVQTSQKTDMRRLLINILSQVQPHQSPDNWKVHSLIS

SIRTHLQDKRYLIIIDGLWATSTWDVIKCALPDGNSSSRILTTTEIEDLALQSCSYDLKF

IFKMKPFGEGDSRKLFFSIVFGSHSKCPPEVSETLYDIVRKCGGLPLAIVTVASLLASQL

EKQEQLDYINKSLGYGLMANPTLEGMKQLLNICYNNLPQHLKVCMLYLSMYQEDHIIWKD

DLVSQWIAEGFICATEGHDKEEISRAYFDELVGRKIIQPVHIDDSGEVLSCVVHHMVLNF

VTYKSIEENFIIAIDHSQATIRFADKVRRLSIHFSNVEDATPPTSMRLSQVRTVAFFGVL

KYMPFVMEFRLIKVLVLHILGDEDSIGIFDLTKISELVRLRYLKVTSNVTVKLPTQMQGL

PYLETLKIDGTISEVPTDIYLPRLLHLTLPAKTNLPSGIVHMTSLRTIGYFDLSCNSAEN

LWSLGELSNLRDLQLTYSEIHSDNLKDNMKYLGSILGKLRNLTSITLSPPGSSCPDTLHI

DRTRINVDGWSSVSSPPALLQRFELLPCVCIFSNLPNWIGQLGNLCILKIGIREVTSNSI

DVLGVLPKLTVLSLYVHTKPAERIVFDNAGFSILKYFEFICSVAWMKFEMGAMPSLRKLK

LGFDVHIADQHDIIPVGIEHLSGLEEISAKIRVACTAHDHCRRFAESALTNAFMMHPGRP

SVNIRCVDWTFHDKDNDCVGTREEECRTPMKQEHFVKEDLSEKSAVLQNEHDEEAHKFVD

RRYYPIMDAAEIRRCPWSVNEEQEQPVLIYDARTKISQSSSMHSEFWAAVQRLTGPAATP

AKTKRHLHLTTSPELEDGFLPVRSLVFPSAPDPRCNMKKKKMRAGPGGGRAVRSNWAPKS*

>NIP_RG014

MGKLFMVLENQYNKHKVLEQEISSLQQEFRMIAAVMDDQLLSMGRSEARTSVAWLHNEEM

LDLEHDIEDCVDRFMHLLTCKHHRGGVRQMAHEVKIRSSFSEEIQKLRRRLSEVRHLVGI

RQPMEELLSLLDEVEGEPKQLRVTSIVGFSGLGKTTLAKAVYDSPHAKDKFCLRAWITAD

GSPETSNWMKEILRGVLQQVRPGDAMDVDGQHLEASLKEYLKDKRYLIIIDDIRMDQWRI

ISSAFENNGTGSRILLTTTIQSVTNRCSHGNGYVYQMNTLGKEDSEELAFSVLRSPELEN

HSESLLGKCDGLPLALVSVSDYLKSSTEATGELCAKLCRDLGSHLTGNHGHDNFSELRKV

LLGNYDSFSGSALSCLLYLGIFPNNHPLRKKVLIRRWLAEGNARSDDPWRSEEDTADDNF

SNLIDQNIIQPVDTRNNSEVKTCKTHGIMHEFLLNRSLAKRFITTSPHDPRVGINTTNSR

HLSVDAAKQTKCMASDEELSRVRSLTIFGDAGDAISYLHKCNLIRVLDLQECSDLNDNHL

KRICVLSPWHLKYLNLGGNISELPRSIEGLHCLETLDLRETEIKFLPIEGIMLPHLAHLF

GKFMLHKDDLNNVKKISKLLKLFSSNKSNLQTLAGFITDGRKGFLQLIGHMKKLRKVKIW

CRHVEGSSNYIADLSKAIQEFTKAPIDMDRVRSLSIDSEEYCEKFLSSLDLEPCSEYSKY

HLSSLKIHGKLLRLPPFFSSLSGLIDLCITPATLTQDHLSALINLNRLLYLKLIADKLEN

LEIKHEALLSLRRLCFVVKSVALAQPKIEQGALPNLVSLQLLCQGLVGLSGIEIRHLKNL

KEVTIDSGVTAQTRQDWEQAAKNHPNRPRVLLLGKVDPVESEEPGRPCAIRGRGKSSIGQ

ESSEDGSDSSLKRMRLAEPSSSSQLQVTGHPHPVVVAATEAASQPSMANL*

>NIP_RG016

MASAAASAFLEAVMGKLFMVLDKEYNKHKALEQEVASLQQEFRMVAAAMDDQLLSMGRSD

AHARTAVARLHAEEMLDLEHDIEDCVDRFTHLLTCSHSNPSGRTSLVCRVKHEVKKVQSR

SSFSEEIQKLRRRLSEAQQRVISNIINPNPPPSGFEPRSSSSTPARAACGPPVGIGEPME

ELLSLLDEVEGEPEQVRVISIVGFGGLGKTTLAKAVYDDPRTKEKFYHRAWIAAVGSPET

SDWMRGILRDVLRQVRPGDAMDVDGQHLEASLREYLKDKRYLIVIDDIDVDQLRIIESIF

PDNGTGSRIIVTTDNQQVANTCSHGNGYVYQMKTLGKEDSKKLAFSGLRSVEPGQGPASL

LAKCDGLPLALVSVSDYLKSSSEPTGELCAELCLNLGSDLKEDGHYSFAQLRKVLLDNYD

SFSGYTLSCLLYLGIFPNNRPLKKKVVIRRWLAEGYARSDDPRRSEEYTADKNFRKLIDR

NIIQPVDTRNNSEVKTCKTHGIMHEFLLNKSLAQRFIGTSLHDHPRVGINTSNARHLSVD

AAKQTECVASDEELSRVRSLTIFGDAGDTISCLRKCKLLRVLDLQECNGLNDDHLKHMYE

LWHLKYLSLGGYINELPRSIQGLHCLETLDLRRTEIKFLPIEAIMLPHLAHLFGKFMLHK

DDLKNAKKMSKLQKFFSSNKSNLKTLAGFITEEGKEFLQLIGHMKKLRKVKIWCKHVEGS

SNYIADLSKAIQEFTKTPIDMDRVRFLSLDSEECSENFLSSIHLEPCSEDYKYTLKSLKL

HGNLLQLPLFVTLLSGLIELCISSATLTQEHLSALTNLNSLLYLKLVADKLENFEIKLGA

FLSLRRLCFVVKNAASALPKFEQGAMPNLVSLQLLCQGLVGLSGIEIRHLKHLKEVTIDS

RVTAQTRQDWEQAAKNHPNRPRVLLLGEVHSVESEEPGRPMEKRRICVGQASSEDERDSS

LKRMRLSDPSSSRLQVIGHPHPVVVTATEAASQPSMAN*

>NIP_RG018

MSSSSSLGSCSASLASSKSSAKSTAQYMRGYPQLSAKSDKRLEELLCHHGSMLPKGADEE

IPLIKQDLEEIISILHGHSEPKLEDHSMVVRCWMKEVRELSYDIEDSIDQYEHAARSQNR

PNIHHRKFNRWRGNKIPCIPQKLKQRLWMANKIREFSLRIQDALQRHAMYNNLGGVAGTA

STTRGDVCSATPWHPTKTQFREHVDNVRSVSIDVDGMEAALNDLNKLKNLLAGIPTASLV

QFREHADKAFMESTCHVGIGAAMNKLENLLDLCGEEKLKVVSIVGVGGVGKTTLANKLYR

KLRWQFECRAFVRTSQKTDMRRLLINILLQIRSHQSPDNWKVHSLISSIRTYLQDKRFLI

VIDDLWATSTWDIIKCALPEGNKSSRILTTTEIEDLALQSCSYDLKFIFKMIPFGEDDSR

KLLFSIVFGSHSKCPPEVSETLYDIVRKCGGLPLAIVTVASLLASQLDKLEQWDYINKSL

GYSLMANPTLEGMKQLLNLCYNNLPQHLKACMLYLSMYQGDHIIWKDDLVNQWIAEGFIC

ATEEHDKEEISRGYFDELVGRKIIQPVHIDDSGEVLSCVVHHIVLNFVTYKSIEENFIIA

IDHSQATIRFADKVRRLSIHFNNVEDAPPPTNMRLFQVRTIAFFGVLKYMPFIMEFRLIK

VLFLHFLGDEDSTGIVDLTKISELVRLRYLKVTSNATVKLPTRLQGLPYLETLKIDGKIS

EVPTDIYLPGLLHLTLPAKTNLPSGIVHMTSLRTIEYFDLSCNSAENLWSLGELSNLRDL

QLTYSEIHSDNLKDNMKYLGSILGKLRNLTSITLSPPGSSCPDTLHIDRDTKTRINVDGW

SSVSSPPALLQRFELLPCVCIFSNLPNWIGQLGNLCILKIGIREVTSNNIDVLGVLPELT

VLSLYVHTKPAERIVFDNAGFSILRYFKFICSVAWMKFEVGAMPSLRKLKLGFDVHRADQ

HDIIPVGIEHLSGLEEISAKIRVASTAHDHCRRFAESALTNAIRMHPGRPSVNIRCVDWT

FDGMDVSNAGTREGECRILKKQQNIVKESSTEKSAVLEKDRGDGANKSDESRYG*

>NIP_RG019

MSMESAAATAFLKTVMGRLFMALEKEYNKHRGLAQESHSLQQDLRMIAAAMDDQQLSMGR

SDAAARTAVARLYTEEMLDLAHDIEDCVDRFLHRLTCNHHKRGGAGAGASLLRRVTHELS

KVKSRSSFGDEIQKLKKRLREAHQRVLTINPPPILIAGGSSSSAAVAPPCRAARSPVGIG

EDVEELLSMLDEVEGEPVQMRVISVVGFGGLGKTTLAKAVYDEPRAKDKFRHRAWVAAGG

SPEIRGILRDVLQQVRPDDAMDVDGQRLEASFKDYLKDKRYLIVIDDIGMDQWSIIRSAF

EDNGTSSRIILTTTIQSVANMWADPEMGQGPGRSMEISAQRKVYMVKGGPIDFFLVKKHT

SLMGLPSQAPGRGPACPGSGSAPCSHGSGYVYQMNTLGEDDSKKLAFPGFRSPELEQGSA

SLLGKCDGLPLALVSVSDYLKSSSEPTGELCAKLCRTLGSHLKEKHGHDNFSELRKVLLD

NYDSLSGYALSCLLYLGIFPSNRPLKKKVVIRRWLAEGYARSDSLRSEEDIADENFSKLI

DRHIIQPIDTRNNSEVKTCKTHGIMHEFLLNKSLTQRFIATSSHDHPRLGINTTNARHLS

VHAGELRECVTSDEELSRVRSLTIFGDASDAISYFRKCKLIRILDLQEWNNLDDDHLKHI

CKLWHLKYLSFGGNISELPRSIEGLHCLETLDLRRTEIKFLPIEAIMLPHLAHLFGKFML

QCLETLDLRRTEIKFLPIEAIMLPHLAHLFGKFMLHKDDLKNVNKSKLQKFFSSNKSNLR

TLAGFITDEGKGFLQLVGHMKKLRKVKVWCKHVAGSSNYIADLSRAIQEFTRTPIDRDSD

RSLSLDCEECSENFLSSIDLEPCSEDSKYHLRSLKLHGKLLRLPPFVTSLSGLTELCISS

AILTQDHLSALIKLNRLLYLKLIADKLENIEIKIGAFPSLRRLCFVMKIMTSALTTIEQG

ALSNLVSLQLLCQGLVGLSGIEIRHLKHLKEITIDSAVTVQTRQDWEQAAKNHPNRPRVL

LFRKVDPMESEEPEKPCAIGEKRKLSVAQPTGSDGGLDSSLKKMRLSEPSSSRLQVIVHP

VVVTATEAAPQHSFANL*

>NIP_RG020

MEAPTCGLWGAVLNLPGRLDGVLLRHGSILPKGAEEEIPLIKRDLHLMISILNGYYSESP

ELEDATATTVARRRCWTKEVRELSYDIEDCIDHYEHAATAGSAGGRTASGGIPPRRKITR

RRWQRTTPLWIPERLKQRLWMANKIREFSLRAQDALKRHAMFCSSVGGNGIATSTASSST

AATGDASSSSSTICWHTTRFRERDFCVPHVGINVAMNKLEDWLTACDDEDQKRLRVVSIV

GVGGIGKTTLANELYRKLRRQFECWAFVRSSQKPDVRRILISILSQLRLQQPPESWKVHS

LISSIRAHLQDKRYLIIVDDICFISTWDIIKCALPDGTSSSRVLTTTQYDDLAVQSCGYD

TKYVFKMKSLSQHDSRYLFFNTVSGSRFIYSPGSTEVSDDIIRKCGSLPLAIVSITSILE

KSRKMEQWGYVNKSLGYNLMKNPTLEGIKQVLDLSYNNLSEHLKPCVLYLSIYQEDYLIC

KDDLVNQWLAEGLICATKDHTKEEISEACFGELVSSKMIQPVHIDGNGDVMSFVIQHMVL

NFISYKSIEENFVTVIHHSQTATKLSDKVRRLSLHFGNVKDAKLPINMRLSQVRTLAFFG

AYKYWWRSIKDQFPLLQVLILHFWHDEDIISFDLTIISQLFRLKYLKITSDVTLELQTKT

RGLQCLETLKIDARISTAPLDTTHLSGLLHLSLPADTNLANGIGHMTSLHTFGYFDLSYN

SVENVLSLGKLTNLRNLQLTCSTIRPNSLEIKLQCLGFILQKLSNLKSVTMSTAGSSCVN

STDASSANVSVRISGDGLSSMSSPPALVERLELLPRICIFSYLPKWISLLSKLRILKIGV

RELVRNDIDVLMGLRALTDLSLHVHTKPTEIIFFGGIGFKALKYLKINCCVAWLKFDMGA

MHSLYKLKLGYNVDGVDQESTIPVGIQYLYGLKEISVKIGGADPEKYDRRAEELAFMIDS

GLHDRCMSITLQCVRQMFDFNEDKSSLTHEEQRKLKQQEILEDDSDEEYDEIIQDSGEQE

VKQ*

>NIP_RG021

MESAVASALLKSVMGRLFTVLEKEYSKHRELAQETNSLQQDLRIVAAAMDDQLLSIRRSD

ARTAVARLHSKEMLDLAHDIEDYVDRFIHHLTCRQQCASAGRNSLLDRVAHELKKVQSRS

SFANEIQKLKRPLRQVHQDVIKNNPLAGGQSSSPSPQDRRIADNPVGIEEPVEELLSLLD

EVEGEPERMRVISIVGFGGLGKTTLAKAVYDSPRVKEKFHLRAWVPAGASPETSSGMRGI

LRAVIQKILPNVAMDVDGQLETSLKEYLKDKRWVLIVIDDIGMDQWSIISSTFKDNGTSS

RIILTTTIQSIANSCSHGNGYVHQMNTLGEEDCKEIALPTGIRSPELETGSVPLLGKCDG

LPLALVSVSDYLKSSCEPTGELCANLCRNLGAHLKEQDGHPSFSELRKVLLDNYDSLSGY

ALSCLLYLGIFPSNRPLKKKVVIRRWLAEGYARSDSLRNEEDIAVENFNKLIDRNIILPV

DTRNNSDVKTCKTHGIMHEFLLNMSLAQRFIMTLSRDHPRLISNARHLSVHDGELTGYVT

SDEEFSRVRSLTVFGDTSDTVSYVRKCKLIRVLDLQECNDFADDHLKHICKLWHLKYLSF

GYNINVLPRSIEGLHCLETLDLRRTKIKFLPIEAVMLPHLAHLFGKFMLHKDDLKNVNKM

SKLNPCKKQKKGMNILPKFFTSKKSNLQTLAGFITGENEGFLQLMGHMKKLRKVKIWCKH

VAGSSNYIADLSQAIQEFTKVPIDSDSNRSLSLDSEECSENFLSALHLEPCSEDFKYHLR

SLKLQGRFLRLPPFVTSLSGLTELFISSATLTQDHLSALITLNRLLYLKLISDKLENFEM

KHGAFPSLRRLCFVVKSVTSDLPTIKQGALPNLVSLHLLCRGLVGLSGIEIRHLKHLKEV

VIDSDVTPQTKQDWAHAAKNHPNRPKFSWPRKVDLVESEEPAKHLKTEKRKYCSNYELDY

NLQEMRLSESRDHKRQKIGEGDTSKSSVGLVYPMYGDVETDRTQVHLFNQETRRYDRTEV

DQKCPEMLQEYKDKHSMVVDVDLRSDEQVNPPHPKLKNLMPGKEYDRQELIPTEGAKVGQ

CQSGGDEDQIVHNTNGKKVVVQANHFFEQEDQGSQVTMSYESSSLLSHMDTKSL*

>NIP_RG025

GEEVDALCKDELMAEVRELSYDMDDAIDEFFLEEPMAGGDGGPFDELKTRVEDVSKRFSD

SRRWRPPVEQHQPSLTAATVDCPPPHARFVHNMMDVSELVEMDKQHEKELIKLLEQGADT

SIYASRWRIATPWHDKEQSTVVKVPEREWGFPDNRNSPFIWASDSFERLRSGSLCGDTLR

LDGEGANIRKLLSTLRNKVGRAQLVQVEDKRKRVEEATKPCEFHEVKTICILGLPGAGKT

TLAKLLYSHHSTTEQQFQHRAFVSLSPGANLTDTLTDILLQVGAYNDDATPYCGTGTPHQ

QYLIDNISAYLIGKKYLIIIDDVWHWEEWEVIRKSIPKNDLGSRIIMTTRLNSIAEKCRN

DDMDAFVYETEALDYVDAWLLCDKVARKSVTCMNINPCYDIVDMCYGMPLALIRVSSALA

EEIQALDSDERQIWRALRRVEDGILDIPSLKPLAESLCLGYDHLPLYLRTLLLCCSVYHW

LDGGIVQRGRLVTRWIAEGFVSEEKAAEGYFDELVGRGWMKHRGLNEYEIHPMMLAILRY

KSKEYNFVTCLGTGSDTCTSASLSYSSPTMAIRRLCLQRGYPMKCFSSMDVSHTRSLVIL

GDVIGVPLDMFKRLRVLDLEDNIGIEDSHLKKICEQLESLRLLKYLGLKGTRITKLPQEI

QKLKQLEILYVRSTGIEELPWEIGELKQLRTLDVRNTRISELPSQIGELKHLRTLDVSNM

WNISELPSQIGELKHLQTLDVRNTSVRELPSQIGELKHLRTLDVRNTGVRELPWQAGQIS

GSLHVHTDDSDEGMRLPEGVCEDLIKGIPKAELAKCSEVLSINIVDRLGSPPIGIFKVIG

LHKSIPKLIKDHFNVLSSLDIRRYNKLEEDDHEFLANNMPNLQMLVLRFEAPQREPIIIN

RTGFQMLERFLVESRVPRITFQEGAMPKLKHLEFKFYAGPPSKDPIGITHLKSLQKVVFR

CSKWYKSDNPGIKAAIDVVKKEARQHPNRPISLLITEGDKEVPNIEAHGSSENIVVVHAA

PDDAISCSSCGRTSTSIQEGTVRDRIPAMDLFWPEFNSYEKAKRN*

>NIP_RG026

MELAVGASEATMRSLLGKLGNLLAQEYSLVSGVRGDIQYINDELASMQAFLRDLSVVTEG

HNHDNRRKDWMKQIRDVAYDVEDCIDDFAHRLPQDSISDAKCSFILTKMYELLTWWPRRD

IASRIAELKVRAQQIADRRNRYGVNNPEHCDSSNSPRPRAHAAAQDIAEYQDTKPQIVSI

KEPVGMKTVMENLEKWLTEPQPDKGRAVLSIVGFGGVGKTTIAMALYRKVSGKFDCQASV

AVSQNYDEDEVLRSILNQVSKQEEAGGSTESSSRDENTREPQGSSSTSSREENTAESGTK

RMLNKLKKALPLSLLGGNDDKTSVRQQETMGSLQLREELKRRLAEKRYILLIDDIWSAKT

WNSIIIPFLPSENDKDSRIIVTTRFHAVGSTCSPRHKNDEATSSPGHGKDLLHKVDFLTG

DKPLDLFNASIPDPMKRTDRDKKLSKICGGLPLAIVTMAGLVACNPNKANSDWSKLCESL

FPYPVTTLNLDGVTRILDCCYNDLPADLKTCLLYLSIFPKGWKISRKRLARRWIAEGFAT

EKQGLTEEEVAEAYFNQLARRNLIRPVEHGSNGKVKAFQVHDMVLEYIMSKSIEENFITV

VGGHWQMTAPSNKVRRLSLQSSGSKHGNSTKGLNLAQVRSLTVFGNLNHVPFHSFNYGII

QVLDLEGWKGLKERHVTEICQMLVLKYLSIRRTEIAKIPSKIEKLEYLETLDIRETYVEE

LPKSVGQLKRISSILGGNKNTRKGLRLPQEKRNKAMKNPSPQGKTKEPAEKGFLSQEKAK

GTMKSLRVLSGIEIVDESAAVAASLHQLTGLRKLAIYKLKISEENDTFKELLSSIEYLGS

CGLQTLAINDENSKFINSLYNMSAPPRYLVSLELSGKLKWLPEWITSITTLNKLTISITV

LTTETLEILRNLPSLFSLTFAFSLSAAKQDQDTVKGILEDNKLATDGEIVIPAKEFKSLK

LLRFFAPFVPKLSFPDKSAMPALEIIEMRFQEFEGLFGIEILENLREVHLKVSDGAEAIT

KFLVSDLKDNTEKPKVFVDGIVTA

>NIP_RLK001

MALGSPVCIIMSALLLITLSPVVAAAAASPPGTSKSNGSDSDLAALLAFKGELSDPYNIL

ATNWTAGTPFCRWMGITCSRRQWQRVTGVELPGVPLQGKLSPHIGNLSFLSVLNLTITNL

TGSIPDDIGRLHRLELLDLGNNALSGVIPASIGNLTRLGVLRLAVNQLSGQIPADLQGLH

SLRSINIQNNGLTGSIPNSLFNNTPLLSYLNIANNSLSGSIPACIGSLPMLQFLDLQVNQ

LAGPVPPGVFNMSMLGVIALALNGLTGPIPGNESFRLPSLWFFSIDANNFTGPIPQGFAA

CQQLQVFSLIQNLFEGALPSWLGKLTNLVKLNLGENHFDGGSIPDALSNITMLASLELST

CNLTGTIPADIGKLGKLSDLLIARNQLRGPIPASLGNLSALSRLDLSTNLLDGSVPSTVG

SMNSLTYFVIFENSLQGDLKFLSALSNCRKLSVLEIDSNYFTGNLPDYVGNLSSTLQAFI

ARRNNISGVLPSTVWNLTSLKYLDLSDNQLHSTISESIMDLEILQWLDLSENSLFGPIPS

NIGVLKNVQRLFLGTNQFSSSISMGISNMTKLVKLDLSHNFLSGALPADIGYLKQMNIMD

LSSNHFTGILPDSIAQLQMIAYLNLSVNSFQNSIPDSFRVLTSLETLDLSHNNISGTIPE

YLANFTVLSSLNLSFNNLHGQIPETGVFSNITLESLVGNSGLCGAVRLGFSPCQTTSPKK

NHRIIKYLVPPIIITVGAVACCLHVILKKKVKHQKMSVGMVDMASHQLLSYHELARATND

FSDDNMLGSGSFGEVFKGQLSSGLVVAIKVIHQHMEHAIRSFDTECQVLRTARHRNLIKI

LNTCSNLDFRALVLEYMPNGSLEALLHSDQRIQLSFLERLDIMLDVSMAMEYLHHEHCEV

VLHCDRLKSNVLFDDDMTAHVSDFGIARLLLGDDSSMISASMPGTVRYMAPEYGALGKAS

RKSDVFSYGIMLLEVFTAKRPTDAMFVGELNIRQWVLQAFPANLVHVIDGQLVQDSSSST

SSIDGFLMPVFELGLLCSSDSPEQRMVMSDVVVTLKKIRKEYVKSIATMGRDENRTAVFH*

>NIP_RLK002

MALGLLVWIYIVLLIALSTVSAASPPGPSKSNGSETDLAALLAFKAQLSDPLSILGSNWT

VGTPFCRWVGVSCSHHRQCVTALDLRDTPLLGELSPQLGNLSFLSILNLTNTGLTGSLPD

DIGRLHRLEILELGYNTLSGRIPATIGNLTRLQVLDLQFNSLSGPIPADLQNLQNLSSIN

LRRNYLIGLIPNNLFNNTHLLTYLNIGNNSLSGPIPGCIGSLPILQTLVLQVNNLTGPVP

PAIFNMSTLRALALGLNGLTGPLPGNASFNLPALQWFSITRNDFTGPIPVGLAACQYLQV

LGLPNNLFQGAFPPWLGKLTNLNIVSLGGNKLDAGPIPAALGNLTMLSVLDLASCNLTGP

IPLDIRHLGQLSELHLSMNQLTGPIPASIGNLSALSYLLLMGNMLDGLVPATVGNMNSLR

GLNIAENHLQGDLEFLSTVSNCRKLSFLRVDSNYFTGNLPDYVGNLSSTLQSFVVAGNKL

GGEIPSTISNLTGLMVLALSDNQFHSTIPESIMEMVNLRWLDLSGNSLAGSVPSNAGMLK

NAEKLFLQSNKLSGSIPKDMGNLTKLEHLVLSNNQLSSTVPPSIFHLSSLIQLDLSHNFF

SDVLPVDIGNMKQINNIDLSTNRFTGSIPNSIGQLQMISYLNLSVNSFDDSIPDSFGELT

SLQTLDLFHNNISGTIPKYLANFTILISLNLSFNNLHGQIPKGGVFSNITLQSLVGNSGL

CGVARLGLPSCQTTSSKRNGRMLKYLLPAITIVVGAFAFSLYVVIRMKVKKHQKISSSMV

DMISNRLLSYQELVRATDNFSYDNMLGAGSFGKVYKGQLSSGLVVAIKVIHQHLEHAMRS

FDTECHVLRMARHRNLIKILNTCSNLDFRALVLEYMPNGSLEALLHSEGRMQLGFLERVD

IMLDVSMAMEYLHHEHHEVALHCDRLKSNVLLDDDDCTCDDSSMISASMPGTVGYMAPEY

GALGKASRKSDVFSYGIMLLEVFTGKRPTDAMFVGELNIRQWVYQAFLVELVHVLDTRLL

QDCSSPSSLHGFLVPVFDLGLLCSADSPEQRMAMNDVVVTLKKIRKDYVKSISTTGSVAL

PAYTKE*

>NIP_RLK003

MALGSLVCLSALLLIPLSTVSAASSPGLTESSNNDTDLTALLAFKAQFHDPDNILAGNWT

PGTPFCQWVGVSCSRHQQRVVALELPNVPLQGELSSHLGNLSFLSVLNLTNTGLTGLLPD

DIGRLHRLELLDLGHNAMLGGIPATIGNLSRLQLLNLQFNQLSGRIPTELQGLRSLININ

IQTNYLTGLVPNDLFNHTPSLRRLIMGNNSLSGPIPGCIGSLHMLEWLVLQHNNLTGPVP

PSIFNMSRLTVIALASNGLTGPIPGNTSFSLPALQRIYISINNFTGQIPMGLAACPYLQT

ISMHDNLFEGVLPSWLSKLRNLTGLTLSWNNFDAGPIPAGLSNLTMLTALDLNGCNLTGA

IPVDIGQLDQLWELQLLGNQLTGPIPASLGNLSSLARLVLNENQLDGSVPASIGNINYLT

DFIVSENRLHGDLNFLSTFSNCRNLSWIYIGMNYFTGSIPDYIGNLSGTLQEFRSHRNKL

TGQLPPSFSNLTGLRVIELSDNQLQGAIPESIMEMENLLELDLSGNSLVGSIPSNAGMLK

NAEHLFLQGNKFSGSIPKGIGNLTKLEILRLSNNQLSSTLPPSLFRLESLIQLNLSQNFL

SGALPIDIGQLKRINSMDLSRNRFLGSLPDSIGELQMITILNLSTNSIDGSIPNSFGNLT

GLQTLDLSHNRISGTIPEYLANFTILTSLNLSFNNLHGQIPEGGVFTNITLQSLVGNPGL

CGVARLGFSLCQTSHKRNGQMLKYLLLAIFISVGVVACCLYVMIRKKVKHQENPADMVDT

INHQLLSYNELAHATNDFSDDNMLGSGSFGKVFKGQLSSGLVVAIKVIHQHLEHALRSFD

TECRVLRMARHRNLIKILNTCSNLDFRALVLQYMPNGSLEALLHSDQRMQLGFLERLDIM

LDVSLAMEYLHHEHCEVVLHCDRLKSNVLFDDDMTAHVSDFGIARLLLGDDNSIISASMP

GTVGYMAPEYGALGKASRKSDVFSYGIMLLEVFTAKRPTDAMFVGELNIRQWVLQAFPAN

LVHVVDGQLLQDSSSSTSSIDAFLMPVFELGLLCSSDSPEQRMVMSDVVVTLKKIRKEYV

KSIATMGRDENQTAVFH*

>NIP_RLK004

MALVRLPVWIFVAALLIASSSTVPCAPSLAATAAGGSASPLQGELSSHLGNISFLFILNL

TNTGLAGSVPNEIGRLHRLELLDLGHNAMSGGIPIAIGNLTRLQLLNLQFNQLYGPIPAE

LQGLHSLGSMNLRHNYLTGSIPDDLFNNTPLLTYLNVGNNSLSGLIPGCIGSLPILQHLN

FQANNLTGAVPPAIFNMSKLSTISLISNGLTGPIPGNTSFSLPVLRWFAISKNNFFGQIP

LGLAACPYLQVIAMPYNLFEGVLPPWLGRLTNLDAISLGGNNFDAGPIPTELSNLTMLTV

LDLTTCNLTGNIPADIGHLGQLSWLHLAMNQLTGPIPASLGNLSSLAILLLKGNLLDGSL

PSTVDSMNSLTAVDVTENNLHGDLNFLSTVSNCRKLSTLQMDLNYITGILPDYVGNLSSQ

LKWFTLSNNKLTGTLPATISNLTALEVIDLSHNQLRNAIPESIMTIENLQWLDLSGNSLS

GFIPSNTALLRNIVKLFLESNEISGSIPKDMRNLTNLEHLLLSDNKLTSTIPPSLFHLDK

IVRLDLSRNFLSGALPVDVGYLKQITIMDLSDNHFSGRIPYSIGQLQMLTHLNLSANGFY

DSVPDSFGNLTGLQTLDISHNSISGTIPNYLANFTTLVSLNLSFNKLHGQIPEGGVFANI

TLQYLEGNSGLCGAARLGFPPCQTTSPNRNNGHMLKYLLPTIIIVVGIVACCLLQELLRA

TDDFSDDSMLGFGSFGKVFRGRLSNGMVVAIKVIHQHLEHAMRSFDTECRVLRMARHRNL

IKILNTCSNLDFKALVLQYMPKGSLEALLHSEQGKQLGFLERLDIMLDVSMAMEYLHHEH

YEVVLHCDRLKSNVLFDDDMTAHVADFGIARLLLGDDNSMISASMPGTVGYMAPVFTAKR

PTDAMFVGELNIRQWVQQAFPAELVHVVDCKLLQDGSSSSSSNMHDFLVPVFELGLLCSA

DSPEQRMAMSDVVVTLNKIRKDYVKLMATTVLQQFIVGVKM*

>NIP_RLK005

MAWLLLPRNSIRMLMLVLQLTVLLVDSTSRLHGVGSSSNGTGDDLSALLAFKARLSDPLG

VLASNWTTKVSMCRWVGVSCSRRRPRVVVGLRLRDVPLEGELTPHLGNLSFLHVLRLTGL

NLTGSIPAHLGRLQRLKFLDLANNALSDTIPSTLGNLTRLEILSLGYNHISGHIPVELQN

LHSLRQTVLTSNYLGGPIPEYLFNATPSLTHIYLGYNSLSGSIPDCVGSLPMLRFLWLSD

NQLSGPVPPAIFNMSSLEAMFIWNNNLTGPLPTNRSFNLPMLQDIELDMNKFTGLIPSGL

ASCQNLETISLQENLFSGVVPPWLANMSRLTILFLGGNELVGTIPSLLGNLSMLRGLDLS

YNHLSGHIPVELGTLTKLTYLYLSLNQLIGTFPAFIGNLSELSYLGLGYNQLTGPVPSTF

GNIRPLVEIKIGGNHLQGDLSFLSSLCNCRQLQYLLISHNSFTGSLPNYVGNLSTELLGF

EGDDNHLTGGLPATLSNLTNLRALNLSYNQLSDSIPASLMKLENLQGLDLTSNGISGPIP

EEIGTARFVWLYLTDNKLSGSIPDSIGNLTMLQYISLSDNKLSSTIPTSLFYLGIVQLFL

SNNNLNGTLPSDLSHIQDMFALDTSDNLLVGQLPNSFGYHQMLAYLNLSHNSFTDSIPNS

ISHLTSLEVLDLSYNNLSGTIPKYLANFTYLTTLNLSSNKLKGEIPNGGVFSNITLISLM

GNAALCGLPRLGFLPCLDKSHSTNGSHYLKFILPAITIAVGALALCLYQMTRKKIKRKLD

ITTPTSYRLVSYQEIVRATESFNEDNMLGAGSFGKVYKGHLDDGMVVAIKDLNMQEEQAM

RSFDVECQVLRMVRHRNLIRILSICSNLDFKALLLQYMPNGSLETYLHKEGHPPLGFLKR

LDIMLDVSMAMEHLHYHHSEVVLHCDRLKSNVLFDEEMTAHVADFGIAKLLLGDDNSAVS

ASMPGTIGYMAPEYVFMGKASRKSDVFSYGIMLLEVFTGKRPTDAMFVGDMSLRKWVSEA

FPARPADIVDGRLLQAETLIEQGVHQNNATSLPRSATWPNEGLLLPVFELGLMCCSSSPA

ERMEINDVVVKLKSIRKDYFAFTGAI*

>NIP_RLK007

MALLLPQNCIHLVILVLLLPLMVLDGFVLGGGGNGTGGDDLSALLAFKAQLSDPLGVLAT

SWTRNASLCRWVGVSCSRRRPRVVVGLRLRSVPLQGELTPHLGNLSFLRVLDLAAANLTG

PIPANLGRLRRVKILDLAHNTLSDAIPSALGNLTKLETLNLYDNHISGHVPMELQNLYSL

RVMALDQNYLTGPIPKHLFDAKHSLTHIYLGDNSLSGPIPDSVASLSMLRVLSLPSNQLS

GPVPPAIFNMSRLETISIRKNNLTGAIPTNESFNLPMLRKIDLYMNKFTGPIPSGLASCK

HLEMISLGGNLFEDVVPAWLATLSQLKSLSLGGNELVGPIPGQLGNLSMLNMLDLSFSNL

SGPIPVELGTLSQLTFMSLSNNQLNGTFPAFIGNLSELSHLELAYNQLTGHVPSTIGNNI

RPLKHFEIRGNHLHGDLSFLSSLSNSQRLEVLIISENLFTGCIPNSVGNLSTGILEFRAN

NNRLIGGLPAILSNLTNLRWINFADNQLSKPILPASLMTLENLLGFDLSKNSIAGPIPKE

ISMLTRLVCLFLSDNKLSGSIPDGIGNLTMLEHIHLSNNKLSSIVPTSIFHLNNLILLLL

FNNALTGALPSDLSHFQNIDHIDVSDNMLDGQLPNSYAYHPMLTYLNLSHNSFRDSIPDS

FSHLTNLATLDLSYNNLSGTIPKYLANFTYLTTLNLSFNKLEGEIPTRGVFSNITLKSLR

GNAGLCGSPRLGLLPCPDKSLYSTSAHHFLKFVLPAIIVAVAAVAICLCRMTRKKIERKP

DIAGATHYRLVSYHEIVRATENFNDDNKLGAGSFGKVFKGRLRDGMVVAIKVLNMQVEQA

MRSFDVECEVLRMVRHRNLIRILSICSNLDFKALLLQYMPNGSLETYLHKEGHPPLGFLK

RLDIMLDVSMAMEHLHYHHSEVVLHCDRLKSNVLFDEEMTAHLADFGIAKLLLGDDNSAV

SASMQGTLGYMAPEYASMGKASRKSDIFSYGIMLLEVLTRKRPTDPMFVGDMSLRKWVSD

AFPARLLDVLDDRLLQGEILIQQGVLQNNDTSLPCSATWANEDLLVAVFELGLMCCSNSP

AERMEINDVVVKLKRIRKDYLTCTKAI*

>NIP_RLK008

MGVGPHCTTSLLIILAVVITSSLLTTTIKADEPSNDTDIAALLAFKAQFSDPLGFLRDGW

REDNASCFCQWIGVSCSRRRQRVTALELPGIPLQGSITPHLGNLSFLYVLNLANTSLTGT

LPGVIGRLHRLELLDLGYNALSGNIPATIGNLTKLELLNLEFNQLSGPIPAELQGLRSLG

SMNLRRNYLSGLIPNSLFNNTPLLGYLSIGNNSLSGPIPHVIFSLHVLQVLVLEHNQLSG

SLPPAIFNMSRLEKLYATRNNLTGPIPYPAENQTLMNIPMIRVMCLSFNGFIGRIPPGLA

ACRKLQMLELGGNLLTDHVPEWLAGLSLLSTLVIGQNELVGSIPVVLSNLTKLTVLDLSS

CKLSGIIPLELGKMTQLNILHLSFNRLTGPFPTSLGNLTKLSFLGLESNLLTGQVPETLG

NLRSLYSLGIGKNHLQGKLHFFALLSNCRELQFLDIGMNSFSGSISASLLANLSNNLQYF

YANDNNLTGSIPATISNLSNLNVIGLFDNQISGTIPDSIMLMDNLQALDLSINNLFGPIP

GQIGTPKGMVALSLSGNNLSSYIPNDSFKGLINLETLDLSHNNLSGGIPKYFSNLTYLTS

LNLSFNNLQGQIPSGGIFSNITMQSLMGNAGLCGAPRLGFPACLEKSDSTRTKHLLKIVL

PTVIVAFGAIVVFLYLMIAKKMKNPDITASFGIADAICHRLVSYQEIVRATENFNEDNLL

GVGSFGKVFKGRLDDGLVVAIKILNMQVERAIRSFDAECHVLRMARHRNLIKILNTCSNL

DFRALFLQFMPNGNLESYLHSESRPCVGSFLKRMEIMLDVSMAMEYLHHEHHEVVLHCDL

KPSNVLFDEEMTAHVADFGIAKMLLGDDNSAVSASMLGTIGYMAPAAAGNMHGFLVPVFE

LGLLCSADSPEQRTAMSDVVVTLKKIRKDYVKLMATTRPGKKLMATTVSVVQQ*

>NIP_RLK009

MAWLLLPPFNSIRLLMLVLPLTIPYASGSIPRDGGSSSNGTGDDLSALLAFKARLSDPLG

VLAGNWTTKVSMCRWVGVSCSRRRPRVVGLKLWDVPLQGELTPHLGNLSFLRVLNLGGIN

LTGPIPADLGRLHRLRILRLAHNTMSDTIPSALGNLTKLEILNLYGNHISGHIPAELQNL

HSLRQMVLTSNYLSDNQLSGPVPPAIFNMSSLEAILIWKNNLTGPIPTNRSFNLPMLQDI

ELDTNKFTGLIPSGLASCQNLETISLSENLFSGVVPPWLAKMSRLTLLFLDGNELVGTIP

SLLGNLPMLSELDLSDSNLSGHIPVELGTLTKLTYLDLSFNQLNGAFPAFVGNFSELTFL

GLGYNQLTGPVPSTFGNIRPLVEIKIGGNHLQGDLSFLSSLCNCRQLQYLLISHNSFTGS

LPNYVGNLSTELLGFEGDDNHLTGGLPATLSNLTNLRALNLSYNQLSDSIPASLMKLENL

QGLDLTSNGISGPITEEIGTARFVWLYLTDNKLSGSIPDSIGNLTMLQYISLSDNKLSST

IPTSLFYLGIVQLFLSNNNLNGTLPSDLSHIQDMFALDTSDNLLVGQLPNSFGYHQMLAY

LNLSHNSFTDSIPNSISHLTSLEVLDLSYNNLSGTIPKYLANFTYLTTLNLSSNNLKGEI

PNGGVFSNITLISLMGNAALCGLPRLGFLPCLDKSHSTNGSHYLKFILPAITIAVGALAL

CLYQMTRKKIKRKLDTTTPTSYRLVSYQEIVRATESFNEDNMLGAGSFGKVYKGHLDDGM

VVAVKVLNMQVEQAMRSFDVECQVLRMVQHRNLIRILNICSNTDFRALLLQYMPNGSLET

YLHKQGHPPLGFLKRLDIMLDVSMAMEHLHYHHSEVVLHCDRLKSNVLFDEEITAHVADF

GIAKLLLGDDNSAVSASMPGTIGYMAPEYAFMGKASRKSDVFSYGIMLLEVFTGKRPTDA

MFVGDMSLRKWVSEAFPARLADIVDGRLLQAETLIEQGVRQNNATSLPRSATWPNEGLLL

PIFELGLMCCSSSPAERMGISDVVVKLKSIRKDYFSFTGAI*

>NIP_RLK010

MALVRLPVWIFVAALLIASSSTVPCASSPGPIASKSNGSETDLAALLAFKAQLSDSNNIL

AGNWTTGTPFCRWVRVSCSSHRRRRQRVTALELPNVPLQGELSSHLGNISFLFILNLTNT

SLTGSVPNEIGRLRRLELLDLGHNAMSAIFNMSKLSTISLISNGLTGPIPGNTSFSLPVL

RWFAISKNNFFGQIPLGLTACPYLQVIAMPYNLFEGVLPPWLGRLTNLDAISLGGNNFDA

GPIPTKLSNLTMLTVLDLTTCNLTGNIPTDIGHLGQLSWLHLAMNQLTGPIPASLGNLSS

LAILLLKGNLLDGSLLSTVDSMNSLTAVDVTKNNLHGDLNFLSTVSNCRKLSTLQMDLNY

ITGILPDYVGNLSSQLKWFTLSNNKLTGTLPATISNLTALEVIDLSHNQLRNAIPESIMT

IENLQWLDLSGNSLSGFIPSSTALLRNIVKLFLESNEISGSIPKDMRNLTNLEHLLLSDN

KLTSTIPPSLFHLDKIVRLDLSRNFLSGALPVDVGYLKQITIMDLSDNHFSGRIPYSTGQ

LQMLTHLNLSANGFYDSVPDSFGNLTGLQTLDISHNSISGTIPNYLANFTTLVSLNLSFN

KLHGQIPEGGVFANITLQYLVGNSGLCGAARLGFPPCQTTSPNRNNGHMLKYLLPTIIIV

VGVVACCLYVMIRKKANHQNTSAGKPDLISHQLLSYHELRATDDFSDDNMLGFGSFGKVF

RGQLSNGMVVAIKVIHQHLEHAMRSFDTKCHVLRMARHRNLIKILNTCSNLDFKALVLQY

MPKGSLEALLHSEQGKQLGFLERLDIMLDVSMAMEYLHHEHYEVVLHCDRLKSNVLFDDD

MTAHVADFGIARLLLGDDNSMISASMPGTVGYMAPEYGTLGKASRKSDVFSYGIMLLEVF

TAKRPTDAMFVGELNIRQWVQQAFPAELVHVVDCQLLQNGSSSSSSNMHGFLVPVFELGL

LCSAHSPEQRMAMSDVVVTLKKIRKDYVKLMATTVSVVQQ*

>NIP_RLK011

MAFRMPVRISVVLLIIALSAVTCASAVPSKSNGSDTDYAALLAFKAQLADPLGILASNWT

VNTPFCRWVGIRCGRRHQRVTGLVLPGIPLQGELSSHLGNLSFLSVLNLTNASLTGSVPE

DIGRLHRLEILELGYNSLSGGIPATIGNLTRLRVLYLEFNQLSGSIPAELQGLGSIGLMS

LRRNYLTGSIPNNLFNNTPLLAYFNIGNNSLSGSIPASIGSLSMLEHLNMQVNLLAGPVP

PGIFNMSTLRVIALGLNTFLTGPIAGNTSFNLPALQWLSIDGNNFTGQIPLGLASCQYLQ

VLSLSENYFEGVVTASAAWLSKLTNLTILVLGMNHFDAGPIPASLSNLTMLSVLDLSWSN

LTGAIPPEYGQLGKLEKLHLSQNQLTGTIPASLGNMSELAMLVLEGNLLNGSLPTTVGSI

RSLSVLDIGANRLQGGLEFLSALSNCRELYFLSIYSNYLTGNLPNYVGNLSSTLRLFSLH

GNKLAGELPTTISNLTGLLVLDLSNNQLHGTIPESIMEMENLLQLDLSGNSLAGSVPSNA

GMLKSVEKIFLQSNKFSGSLPEDMGNLSKLEYLVLSDNQLSSNVPPSLSRLNSLMKLDLS

QNFLSGVLPVGIGDLKQINILDLSTNHFTGSLSDSIGQLQMITYLNLSVNLFNGSLPDSF

ANLTGLQTLDLSHNNISGTIPKYLANFTILISLNLSFNNLHGQIPKGGVFSNITLQSLVG

NSGLCGVAHLGLPPCQTTSPKRNGHKLKYLLPAITIVVGAFAFSLYVVIRMKVKKHQMIS

SGMVDMISNRLLSYHELVRATDNFSYDNMLGAGSFGKVYKGQLSSSLVVAIKVIHQHLEH

AMRSFDAECHVLRMARHRNLIKILNTCTNLDFRALILEYMPNGSLEALLHSEGRMQLGFL

ERVDIMLDVSMAMEYLHHEHHEVVLHCDRLKSNVLLDDDMTAHVSDFGIARLLLGDDSSM

ISASMPGTVGYMAPEYGALGKASRKSDVFSYGIMLLEVFTGKRPTDAMFVGELNIRQWVY

QAFPVELVHVLDTRLLQDCSSPSSLHGFLVPVFELGLLCSADSPEQRMAMSDVVVTLKKI

RKDYVKSISTTGSVALPAYTKE*

>KAS_RG001

MATILDSLIGSCVNKLQGIITEEAILILGVEEELRKLQERMKQIQCFISDAERRGMEDSA

VHNWVSWLKDAMYDADDIIDLASFEGSKLLNGHSSSPRKSFACSGLSFLSCFSNIRVRHK

IGDKIRSLNQKLEEIAKDKIFATLENTQSSHKVSTSELRKSSQIVEPNLVGKEILHACRK

LVSQVLTHKEKKAYKLAIIGTGGIGKTTLAQKVFNDQKLKRSFDKHSWICVSQDYSPASI

LGQLLRTIDVQYKQEESVGELQSKIESAIKDKSYFLVLDDVWQSDVWTNLLRTPLYAATS

GIILITTRQDTVAREIGVEEPHHVDLMSPAVGWELLWKSINIEDDKEVQNLRDIGIEIVQ

KCGGLPLAIKVIARVLASKDKTENEWKKILANNVWSMAKLPKEITGALYLSYDDLPQHLK

QCFLYCIVYPEDWTIDRDYLIRMWVAEGFVEVHKDQLLEDTGEEYYYELISRNLLQPVVG

SFDQSECKMHDLLRQLACYISREECYIGDPTSMVDNNMRKLRRILVITEKDMVVIPSMGK

EEIKLRTFRTQQNPLGIEKTFFMRFVYLRVLDLSDLLVEKIPDCLGNLIHLRLLDLDGTL

ISSVPESIGALKNLQMLHLQRCKSLHSLPSAITRLCNLRRLDIDFTPINKFPRGIGRLQF

LNDLEGFPVGGGSDNTKMQDGWNLQELAHLSQLRQLDLNKLERATPRSSTDALLLTDKKH

LKKLNLCCTKPTDEEYSEKGISNVEMIFEQLSPPRNLEDLMIVLFFGRKFPTWLSTSQLS

SLTYLRLIDCKSCVHLPPTGQLPILKYLKIKGARAITKIGPEFVGCWEGNLRSTEAVAFP

KLEVLVIKDMPNWEEWSFVEEEEELQEEETTAAAKEGGEDGTAASKQKGEEAPSPTPRSS

WLLPCLTRLQLVGCPKLRALPPQLGQQATNLKELFIRDTRYLKTVEDLPFLSGWLLVERC

EGLERISNLPQVRELRVNVCPNLRHVEELGGLEQLWLDEGMQDISQLWVPGLQEQHRQLH

GDEHELEVIEWL*

>KAS_RG002

MEESAVDNWLGQLREVLYDVDDIIDLARFKGSILLTDHPSSSSRKSIACTGLSISTCFSN

VQARHEVAVKIRSLNRKIENISKDRVFLTLKSTVPTGSSSVLRVRKSSHLLEPNIVGKEI

IHACRKMVDLVLEHKGRKLYKLAIVGTGGVGKTTLAQKIYNDRKIKGSFNKKAWVCVSKV

YSEASLLRELLRIMEVHHDQDESIGELQSKLEIAIKETSFFLVLDDMWQSDAWTNLLRIP

LHAAEMGAILITTRNNIVALEIGVDHTYRVDLMSTDVGWELLWKSMNISESIELQTLQDV

GIEIVRKCGCLPLAIKVIARVLASKEQTENEWKKILSKNAWFMNNLPNDLRGALYLSYDE

LPRHLKQCFLYCSVYPEDANIYRDDLTRMWIAEGFIEDHGGQLLEETADEYYYELIHRNL

LQPDGLYYDHSSCKMHDLLRQLACYLSREECFVGNPESLVGNTVSKLRRVSVVTDKNMVM

LPSMDEVQYKVRTWKTSYEKTLRVDNSFFKRFPYLRVLDLTDSFVPSIPGCIGNLIHLRL

LDLDGTNVSCLPESIGNLKNLQILNLERCVALHSLPSAITQLCNLRRLGLNYSPIYQVPK

GIGKLEFLNDVEGFPVYGGSSNTKMQDGWNLEELAYLYQLRRLHMIKLERAAYRTTYPLL

TDKGFLKFLYLWCTERTDEPYTEKDFSNIEKIFEQLIPPCNLEDLAIVKFFGRQYPFWID

STHLAYVKSLHLFNCKFCMHLPPVGQLPNLKYLKIEGAAAVTIIGPEFAGLRASNLGRTV

AFPKLEELLIRDMPNWEEWFFIDETTSTAKERVDDGDSAMPKEKALPPRMQILSRLRRLE

LSGCPKLKALPRQLAQINSLKEIELRWVSSLKVVENFPLLSETLLIATCQALEKVSNLPQ

VRELRVQDCPNLRLVEELGTLEQLWLYEDMHEVSTLWVPGLQQQCRQCHGEDFDVYNWT*

>KAS_RG003

MATIVDTLVGSCINKLQAIITDKTILILGVKDELEELQRRTNVIRSSLQDAEARRMEDSV

VEKWLDQLRDVMFDVDDIIDLARFKGSVLLPDYPMSSSRKSTACSGLSLSSCFSNIRIRH

EVAVKIRSLNKKIDNISKDEVFLKLNRRHHNGSGSAWTPIESSSLVEPNLVGKEVIRACR

EVVDLVLAHKKKNVYKLAIVGTGGVGKTTLAQKIFNDKKLEGRFDHHAWACVSKEYSRDS

LLRQVLRNMGIRYEQDESVPELQRKIKSHIANKSFFLVLDDVWNSEAWTDLLSTPLHAAA

TGVILITTRDDTIARVIGVDHTHRVDLMSADVGWELLWRSMNINQEKQVQNLKDIGIEIV

RKCGGLPLAIRVIATVLASQEQTENEWRRILGQNAWSMSKLPRELSGALYLSYEVLPHQL

KQCFLYCALFREDATIWRDDLTRMWVAEGFIDEEKGQLLEDTAERYYYELIHRNLLQPDG

LYFDRFSCKMHDLLRQLACYLSREECFVGDVESLGTNTMCKVRRISVVTEKDMMVLPSIN

KDQYKVRTYRTSYQKALQVDSSLFEKLTYLRVLDLTNSHVQRIPNCIENMIHLRLLDLDG

TDISHLPESIGSLQNLQILNLQRCKSLHRLPLATTQLCNLRRLGLDGTPINQVPKGIGRL

KFLNDLEGFPIGGGNDNTKIQDGWNLEELAHLSQLRCLDMIKLERATPCSSTDPFLLTEK

KHLKVLNLCCTEPTDEAYSEEGIGNVEMIFEQLSPPRNLEDLMIVLFFGRKFPTWLSTSQ

LSSLTYLRLIDCKSCVHLPPIGQLPNLKYLKINGASAITKIGPEFVGCWEGNLRSTEAVA

FPKLEGLVIKDMPNWEEWSFVEEEEELQKEEAAAAAKEGGEDGTAASKQKGEEAPSLTPK

SSWLLPCLTRLQLVGCPKLRALPPQLGQQATNLNDLFIRDTRYLKTVEDLPFLSGLLLVE

RCEGLERISNLPQVRELFVNRCLNLRHVEELGGLEQLLLDEGMQEISQLWVPGLEEQHRQ

LHGDEHELEVIEWL*

>KAS_RG004

MATILDSLIGSCAKKLQEIITEEAILILGVKEDLRELQEKMEQIRCFISDVERRGMEDSS

IHNWISRLKDAMYDADDIIDLASFEGSKLLNGHSCSPRKTIACSGLSLLSCFSNIRVHHE

IGNKIRSLNRKLEEIAKDKIFVTLENTQSSHKDSTSELRKSSQIAESNLVGKEILHASRK

LVSQVLTHKEKKTYKLAIIGTGGIGKTTLAQKVFNDGKLKQSFDKHAWICVSQDYSPASV

LGQLLRTIDAQCKQEESVGELQSKLESAIKDKSYFLVLDDVWQSDVWTNLLRTPLYAATS

GIVLITTRQDTVAREIGVEEPHHIDLMSPAVGWELLWKSINIEDEKEVQNLRDIGIEIVQ

KCGGLPLAIKVIARVLASKDKTENEWKKILVDKLPKEIRGAQYLSYDDLPQHLKQCFLYC

IVYPEDCTIRRDDLIRLWVAECFVEVHKDQLLEDTAEEYYYELISRNLLQPVDTSFDPSK

CKMHDLLRQLACHLSREECYIGDPTSLVDNNMCKLRRILAITEKGMVVIPSMGKEEIKLR

TFRTQPNPLGIEKTFFMRFTYLRVLDLTDLLVEEIPDCVGYLIHLRLLDLGGTNISCLPN

SIGALKNLQMCNIPWCKSLYGLPSTITRLSNLRRLGLDFTPINQVPRGIGRLEFLNDLEG

FPVGGGSDNTKMQDGWNLQELAHLSQLRQLDLNKLERATPRSSTDALLLTDKKHLKKLNL

CCTKPTDEEYSEKGISNVEMIFEQLSPPRNLEDLMIVSFFGRKFPTWLSTSQLSSLTYLK

LIDCKSCLQLPPIGQIPNLKYLKIKGASAITKIGPEFVGSWEGNLTSTETIAFPKLELLI

IEDMPNWEEWSFVEEEEEKEVQEEEAAAAAKEGREDGTAASKQKGEEAPSPTPRSLWLLP

CLNELELVNCPKLRALPPQLGQQATNLKELDIRRARCLKTVEHLPFLSAMAVKEYAEDLF

TVGPRA*

>KAS_RG005

MATIVDTLVGSCINKLQALITDKAILIFRVKNKLEELLRRTDFIRSSLNDAEERRMEDST

VEKWLDQLRDVMYDVDDIIDLARFKGSVLLPDHPMSLSRKLTACSGLSLSSCFSNIRIRH

EVAVKIRSLNKKIENISKDEKAPAWLNPTLWDACREVVDLVLAHKAKNVYKLAIVGTGGV

GKTTLAQKIFNDKKLEGRFDHRAWVCVSKEYSMVSLLTQFLSNMQIHYEQNEPVGNLQSK

LKAGIADKSFFLVLDDVWHYKAWEDLLRTPLNAAATGIILVTTRDETIARVIGVDRTHRV

DLMSADVGWELLWRSMNVKEEKQVKNLRDTGIEIVRKCGGLPLAIRAIAKVLASLQDQTE

NEWRQILGKNAWSMSKLPDELNGALYLSYEVLPHQLKQCFLYCALFPEDATIFRDDLTRM

WVAEGFIDEEKGQLLEDTAERYYYELIYRNLLQPVGLYFDHSRCKIHDLLRHLACHLSRE

ECFVGDPESLGSNSMCKIRRILVVTEKDIVVLPSMDKERYKVRTYRISYEKPLQVDSSHF

KKLKYLRVLDLTNSHVQRIPNYIENMIHLRLLDLDGTDISHLPESIGSLQNLQILNLQRC

KSLHSLPLATTQLCNLRWLGLAGTPINQVPKGIGRLKFLNDLEGFPIGGESDNTKIQDGW

NLEELAHLSQLRCLDMIKLERATPCSSTDSFLLAEKKLLKLLALWCTEQTDESYSEENAS

SVENIFKKLTPPHNLEKLVIVNFSGCRFPTWLGTTHLPSVKSVILTNCKSCVHLPPIGQL

PNLNYLKIIGASAITKIGPEFIGCREGNLRSTEAVAFPKLEWLIIKDMPNWEEWSFVVEE

DVVEEGASAAAKEGGEDGTAASKQKGEETLSPRSLWLLPCLTKLELQDCPELRALPPQLG

QQATNLKELDIREEKCLKTVEDLPFLSGYLLVEACEGLERISNLPQVRELYVNLCPNLRH

VEELGSLEQLGLTKNMQEISKLWVPRLQEQHRRLHGDEHELEVNEWL*

>KAS_RG006

MATILDSLVGSCANKLKEIITEEVILILGIQEELAELQRKTELIHCCISDAEARRMEESA

VDNWLGQLREVLYDVDDIIDLARFKGSILLTDHPSSSSRKSIACTGLSISTCFSNVQARH

EVAMKIRSLNRKIENISKDRVFLTLKSTVPTGSSSVLRGRKLYKLAIVGTGGVGKTTLAQ

KIYNDRKIKGSFNKKAWVCVSKVYSEASLLRELLRIMEVHHDQDESIGELQSKLEIAIKE

TNFLLVLDDMWQSDAWENLLRIPLHAAETGTILITTRNNIVALEIGVDHTYRVDLMSTDV

GWELLWKSMNISESIELQTLQDVGIEIVRKCGCLPLAIKVIARVLASKEQTENEWKKILS

KNAWFMNNLLNDLRGFIEDHGGQLLEETADEYYYELIHQNLLQPDGLYYDHSSCKMHDLL

RQLACYLSREECFVGNPESLVGNTVSKLRRVSVVTDKNMVMLPRMDEVQYKVRTWKTSYE

KTLRVDNSFFKRFPYLRVLDLTNSFVPSIPGCIGNLIHLRLLDLDGTNSPSAITQLCNLR

RLGLNYSPIYQVPKGIGKLEFLNDVEGFPVYGGCSNTKMQDGWNLEELAYLYQLRRLHMI

KLERAAYRTTYVPIVNRQRSPVWEHAQAAGWATVWLTKPNLVDKKISGSEQVADEQVVTV

ADGDMVLSAQPEKEMTAAMLLKSSGHAGSHYPETGSNEAVAVGLAPHMTPIRRSDRSNSE

TALGIASADDDSLLKAMKRKAAINLHDQFAPYGARPALLASPQGTPNEPFTSSCSASQLS

FCSSLNRIGVSLGNNSAEVDFSIKALKHINVDKLKVIPKANSSFSLNHCRHG*

>KAS_RG007

MATILGSLVGSCVNKLQGIITEEAILILGVKEELRKLQERMKQIQCFINDAERRGMEDSA

VHNWISRLKDVMYDADDIIDLASFEGNKLLNGHSSSPRKTTACSALSPLSCFSNIRVRHE

IGDKIRTLNRKLAEIEKDKIFATLENTQPADKGSTSELRKTSHIVEPNLVGKEIVHASRK

LVSLVVAHKEDKAYKLAIVGTGGIGKTTLAQKVFNDQKLKGTFNKHAWICVSQDYTPVSV

LKQLLRTMEVQHAQEESAGELQSKLELAIKDKSFFLVLDDLWHSDVWTNLLRTPLHAATS

GIILITTRQDIVAREIGVEEAHRVDLMSPAVGWELLWKSMNIQDEKEVQNLRNIGIEIVQ

KCGGLPLAIKVTARVLASKDKTENEWKRILAKNVWSMDKLPKEISGALYLSYDDLPLHLK

QCFLYCIVFPEDWTLKRDELIMMWVAEGFVEVHKDQLLEDTAEEYYYELISRNLLQPVDT

YFDQSRCQMHDLLRQLACYLSREECHIGDLKPLVDNTICKLRRMLVVGEKDTVVIPFTGK

EEIKLRTFTTDHQLQGVDNTFFMRLTYLRVLDLSDSLVQTIPDYIGNLIHLRMFDLDGTN

ISCLPESIGSLQNLLILNLKRCKYLHFLPLATTQLYNLRRLGLADTPINQVPKGIGRLKF

LNDLEGFPIGGGSDNKKMQDGWNLEELAYLPQLRQLGMIKLERGTPRSSTDPFLLTEKKH

LKVLNLHCTEQTDEAYLEENARNIEKIFEKLTPPHNLEKLVIVNFFGCRFPTWLGTNHLP

SVKYVVLIDCKSCVHLPPIGQLPNLKYLKINGASAITKIGPEFVGCWEGNLRSTEAVAFP

KLEELVIVDMPNWEEWSFVEEEEVQEEEAAAAAKEGGEDGTAASKQKGEEAPSSRSSWLL

PCLTRLQLVGCPMLRALPPQLGQQATNLKGLLIRYTSCLKTVEDLPFLSGYLLVDGCEGL

ERVSNLPQVRELLVNVCPNLRHVEELGGLEQLLLDEGMQEISSLWVPRLQEQHRQLHGDE

HELEVTEWL*

>KAS_RG008

MATIVGTLVGSCVNKLQGIITEEAILILGVKDELEELQRRTNVIRYSLQDAEARRMKDSA

VEKWLDQLRDVMYDVDDIIDLARFKGSVLLPNYPMSSSRKSTACSGLSLSSCFSNIRIRH

EVAVKIRSLNKKIDNISKDDVFLKLSRTQHNGSGSAWTPIESSSLVEPNLVGKEVVHACR

EVVDLVLAHKAKNVYKLAIVGTGGVGKTTLAQKIFNDKKLEGRFDHRAWVCVSKEYSMVS

LLTQVLSNMKIHYEQNESVGNLQSKLKAGIADKSFFLVLDDVWHYKAWEDLLRTPLNAAA

TGIILVTTRDETIARVIGVDRTHRVDLMSADVGWELLWRSMNIKEEKQVKNLRDTGIEIV

RKCGGLPLAIRAIAKVLASLQDQTENEWRQILGKNAWSMSKLPDELNGALYLSYEVLPHQ

LKQCFLYCALFPEDATIFCGDLTRMWVAEGFIDEQEGQLLEDTAERYYHELIHRNLLQPD

GLYFDHSSCKMHDLLRQLASYLSREECFVGDPESLGTNTMCKVRRISVVTEKDIVVLPSM

DKDQYKVRCFTNLSGKSARIDNSLFERLVCLRILDLSDSLVHDIPGAIGNLIYLRLLDLD

RTNICSLPEAIGSLQSLQILNLQGCESLRRLPLATTQLCNLRRLGLAGTPINQVPKGIGR

LKFLNDLEGFPIGGGNDNTKIQDGWNLEELAYLPQLRQLGMIKLERGTPRSSTDPFLLTE

KKHLKVLNLHCTEQTDEAYSEENARNIEKIFEKLTPPHNLEDLFVGNFFGCRFPTWLSTS

QLSSLTYLKLKDCKSCVHLPLIGQLPNLKYLRINGASAITKIGPEFVGCWEGNLRSIEAV

AFPKLEVLVINDMPNWEEWSFVEEEEEVQEEETAAAAKEGGEDGTAASKQKGEEAPSPTP

RSSWLLPCLNELGLVNCPKLRALPPQLGQQATNLKKLFIRYTSCLKTVEDLPFLSGFLQV

EGCEGLERISNLPQVRELRVNVCSNLRHVEELGGLEQLLLDEGMQEISQLWVSGLQEQHR

QLHGDEHELEVIEWL*

>KAS_RG010

MATIVDTLVGSCINKLQALIIDKAILIFRVKNKLEELLRRTDFIRSSLNDAEERRMEDST

VEKWLDQLRDVMYDVDDIIDLARFKGSVLLPDHPMSLSRKLTACSGLSLSSCFSNIRIRH

EVAVKIRSLNKKIENISKDEVFLKLSRTQHNGSGSAWTPIESSSLVEPNLVGKEVIHACR

EVVDLVLAHKAKNVYKLAIVGTGGVGKTTLAQKIFNDKKLEGRFDHRAWVCVSKEYSMVS

LLTQFLSNMQIHYEQNEPVGNLQSKLKAGIADKSFFLVLDDVWHYKAWEDLLRTPLNAAA

TGIILVTTRDETIARVIGVDRTHRVDLMSADVGWELLWRSMNVKEEKQVKNLRDTGIKIV

RKCGGLPLAIRAIAKVLASLQDQTENEWRQILGKNAWSMSKLPDELNGALYLSYEVLPHQ

LKQCFLYCALFPEDATIFRDDLTRMWVAEGFIDEEKGQLLEDTAERYYYELIYRNLLQPV

GLYFDHSRCKIHDLLRHLACHLSREECFVGDPESLGSNSMCKIRRILVVTEKDIVVLPSM

DKERYKVRTYRISYEKPLQVDSSHFKKLKYLRVLDLTNSHVQRIPNYIENMIHLRLLDLD

GTDISHLPESIGSLQNLQILNLQRCKSLQSLPLATTQLCNLRWLGLAGTPINQVPKGIGR

LKFLNDLEGFPIGGESDNTKIQDGWNLEELAHLSQLRCLDMIKLERATPCSSTDSFLLAE

KKLLKLLALWCTEQTDESYSEENASSVENIFKKLTPPHNLEKLVIVNFSGCRFPTWLGTT

HLPSVKSVILTNCKSCVHLPPIGQLPNLNYLKIIGASAITKIGPEFIGCREGNLRSTEAV

AFPKLEWLIIKDMPNWEEWSFVVEEEEDVVEEGASAAAKEGGEDGTAASKQKGEETLSPR

SLWLLPCLTKLELQDWPELRALPPQLGQQATNLKELDIREEKCLKTVEDLPFLSGYLLVE

ACEGLERISNLPQVRELYVNLCPNLRHVEELGSLEQLGLTKNMQEISKLWVPRLQEQHRR

LHGDEHELEVNEWL*

>KAS_RG011

MATILDSLVGSCANKLKEIITEEVILILGIQEELAELQRKTELIHCCISDAEARRMEESA

VDNWLGQLREVLYDVDDIIDLARFKGSILLTDHPSSSSRKSIACTGLSISTCFSNVQARH

EVAVKIRSLNRKIENISKDRVFLTLKSTVPTGSSSVLRVRKSSHLLEPNIVGKETIHAYR

KMVDLVLEHKGRKLYKLAIVGTGGVGKTTLAQKIYNDRKIKGSFNKKAWVCVSKVYSEAS

LLRELLRIMEVHHDQDESIGELQSKLEIAIKETNFLLVLDDMWQSDAWTNLLRIPLHAAE

TGTILITTRNNIVALEIGVDHTYRVDLMSTDVGWELLWKSMNISESIELQTLQDVGIEIV

RKCGCLPLAIKVIARVLASKEQTENEWKKILSKNAWFMNNLLNDLRGVLYLSYDELPRHL

KQCFLYCSVYPEDANIYRDDLTRMWIAEGFIEDHGGQLLEETADEYYYELIHQNLLQPDG

LYYDHSSCKMHDLLRQLACYLSREECFVGNPESLVGNTVSKLRRVSVITDKNMVMLPRMD

EVQYKVRTWKTSYEKTLRVDNSFFKRFPYLRVLDLTNSFVPSIPGCIGNLIHLRLLDLDG

TNVSCLPESIGNLKNLQILNLERCVALHSLPSAITQLCNLRRLGLNYSPIYQVPKGIGKL

EFLNDVEGFPVYGGCSNTKMQDGWNLEELAYLYQLRRLHMIKLERAAYRTTYPLLVRQRD

EPYTEKDFSNIEKIFEQLIPPCNLEDLAIVKFFGRQYPFWIDSTHLAYVKSLHLFNCKFC

MHLPPVGQLPNLKYLKIEGAAAVTIIGPEFAGLRASNLGRTVAFPKLEELLIRDMPNWEE

WFFIDEATSTAKERVDDGDTVTLKALPRQLAQINSLKEIELRWASSLKVVENFPLLSEML

LIATCQALEKVSNLPQVRELRLQDCPNLRLVEDLSTLEQLWLYEDMHEVSTLWVPGLQQQ

CRQHHGEDLDVYNWT*

>KAS_RG012

MATILGSLVGSCVNKLQGIITEEAILILGVKEELRKLQERMKQIQCFINDAERRGMEDSA

VHNWISRLKDVMYDADDIIDLASFEGNKLLNGHSSSPRKTTACSALSPLSCFSNIRVRHE

IGDKIRTLNRKLAEIEKDKIFATLENTQPADKGSTSELRKTSHIVEPNLVGKEIVHACRK

LVSLVVAHKEDKAYKLAIVGTGGIGKTTLAQKVFNDQKLKGTFNKHAWICVSQDYTPVSV

LKQLLRTMEVQHAQEESAGELQSKLELAIKDKSFFLVLDDLWHSDVWTNLLRTPLHAATS

GIILITTRQDIVAREIGVEEAHRVDLMSPAVGWELLWKSMNIQDEKEVQNLRDIGIEIVQ

KCGGLPLAIKVTARVLASKDKTENEWKRILAKNVWSMAKLPKEISGALYLSYDDLPLHLK

QCFLYCIVFPEDWTIYHDELIMMWVAEGFVEVHKDQLLEDTAEEYYYELISRNLLQPVYQ

YFDQSRCKMHDLLRQLACYLSREECHIGDLKPLVDNTICKLRRMLVVGEKDTVVIPFTGK

EEIKLRTFTTDHQLQGVDNTFFMRLTHLRVLDLSDSLVQTIPDYIGNLIHLRLFDLDGTN

ISCLPESIGSLQNLLILNLKRCKYLHFLPLATTQLYNLRRLGLADTPINQVPKGIGRLKF

LNDLEGFPIGGGSDNTKMQDGWNLEELAYLPQLRQLDMIKLERGTPRSSTDPFLLTEKKH

LKVLNLHCTKQTDEAYSEENARNIEKIFEKLTPPDNLEGLFVRNFFGCRFPTWLGTNHLP

SVKYVVLIDCKSCVHLPPIGQLPNLKYLRINGASAITKIGPEFVGCWEGNLRSTEAVAFP

KLEALVIEDMPNWEEWSFVEEEEVQEEEAAAAAKEGGEDGTAASKQKGEEAPSSRSSWLL

PCLTRLDLVGCPKLRALPPQLGQQATNLKEFLIRYTSCLKTVEDLPFLSGCLLVEGCVGL

ERVSNLPQVRELFVNECPNLRHVEELGGLEQLWLDEGMQEISSLWVPRLQEQHRQLHGDE

HELEVTEWL*

>KAS_RG015

MATILDSLVGSCANKLKEIITEEVILILGIQEELAELQRKTELIHCCISDAEARRMEESA

VDNWLGQLREVLYDVDDIIDLARFKGSILLTDHPSSSSRKSIACTGLSISTCFSNVQARH

EVAVKIRSLNRKIENISKDRVFLTLKSTVPTGSSSVLRVRKSSHLLEPNIVGKEIIHACR

KMVDLVLEHKGRKLYKLAIVGTGGVGKTTLAQKIYNDRKIKGSFNKKAWVCVSKVYSEAS

LLRELLRIMEVHHDQDESIGELQSKLEIAIKETSFFLVLDDMWQSDAWTNLLRIPLHAAE

MGAILITTRNNIVALEIGVDHTYRVDLMSTDVGWELLWKSMNISESIELQTLQDVGIEIV

RKCGCLPLAIKVIARVLASKEQTENEWKKILSKNAWFMNNLPNDLRGALYLSYDELPRHL

KQCFLYCSVYPEDANIYRDDLTRMWIAEGFIEDHGGQLLEETADEYYYELIHRNLLQPDG

LYYDHSSCKMHDLLRQLACYLSREECFVGNPESLVGNTVSKLRRVSVVTDKNMVMLPSMD

EVQYKVRTWKTSYEKTLRVDNSFFKRFPYLRVLDLTDSFVPSIPGCIGNLIHLCLLDLDG

TNVSCLPESIGNLKNLQILNLERCVALHSLPSAITQLCNLRRLGLNYSPIYQVPKGIGKL

EFLNDVEGFPVYGGSNEPYTEKDFSNIEKIFEQLIPPCNLEDLAIVKFFGRQYPFWIDST

HLAYVKSLHLFNCKFCMHLPPVGQLPNLKYLKIEGAAAVTIIGPEFAGHRASNLGRTVAF

PKLEELLIRDMPNWEKWFFIDEATSTAKERVDDGDSAMPKEKALPPRMQILSRLRRLELS

GCPKLKALPRQLAQINSLKEIELRWASSLKVVENFPLLSEMLLIATCQALEKVSNLPQVR

ELRMQDCPNLRLVEDLSTLEQLWLYEDMHEVSTLWVPGLQQQCRQHHGEDLDVYNWT*

>KAS_RG016

MATILGSLVGSCVNKLQGIITEEAILILGVKDELEELQRRTDLIRYSLQDAEARRMKDSA

VQKWLDQLRDVMYDVDDIIDLARFKGSVLLPNYPMSSSRKSTACSGLSLSSCFSNIRIRH

EVAVKIRSLNKKIDNISKDDVFLKLSRTQHNGSGSAWTPTESSSLVEPNLVGKEVVHACR

EVVDLVLAHKAKNVYKLAIVGTGGVGKTTLAQKIFNDKKLEGRFDHHAWVCVSKEYSMVS

LLTQVLSNMKIHYEQNESVGNLQSKLKAGIADKSFFLVLDDVWHYKAWEDLLRTPLNAAA

MGIILVTTRDETIARVIGVDRTHRVDLMSADVGWELLWRSMNIKEEKQVKNLRDTGIEIV

RKCGGLPLAIRAIAKVLASLQDQTENEWRQILGKNAWSMSKLPDELNGALYLSYEVLPHQ

LKQCFLYCALFPEDANIFCGDLTRMWVAEGFIDEQEGQLLEDTAERYYHELIHRNLLQPD

GLYFDHSWCKMHDLLRQLASYLSREECFVGDPESLGTNTMCKVRRISVVTEKDIVVLPSM

DKDQYKVRCFRNLSGKSARIDNSLFKRLVCLRILDLSDSLVHDIPGAIGNLIYLRLLDLD

RTNICSLPEAIGSLQSLQILNLESCKSLHRLPLATTQLCNLRRLGLAGTPINQVPKGIGR

LKFLNDLEGFPIGGGNDNTKIQDGWDLEELGHLSQLRCLDMIKLERATPCSSTDPFLLSE

KKHLKVLNLHCTEQTDEAYSEEGISNVEKIFEKLEPPHNLEDLVIGDFFGRRFPTWLGCT

HLSSVKYVLLIDCKSCVHLPPIGQLPNLKYLKINGASAITKIGPEFVGCWEGNLRSTEAV

AFPKLEALVIEDMPNWEEWSFVEEEEVQEEEAAAAAKEGGEDGIAASKQKGEEAPSPTPR

SSWLLPYLTRLDLVGCPKLRALPPQLGQQATNLKDLLIRDTRYLKTVEDLPFLSSGLQVE

GCEGLERVSNLPQVRKLFVNVCPNLRHVEELGGLEQLLLEKGMQEISQLWVPGLQEQHRQ

LHGDEHELEVTEWL*

>KAS_RG017

MATILDSLVGSCANKLKEIITEEVIVILGIQEELAELQRKTELIHCCISDAEARRMEESA

VDNWLGQLREVLYDVDNIIDLARFKGSILLTDHPSSSSRKSIACTGLSISTCFSNVQARH

EVAVKIRSLNRKIENISKDRVFLTLKSTVPTGSSSVLRVRKSSHLLEPNIVGKEIIHACR

KMVDLVLEHKGRKLYKLAIVGTGGVGKTTLAQKIYNDRKIKGSFNKKAWVCVSKVYSEAS

LSRELLRIMEVHHDQDESIGELQSKLEIAIKETSFFLVLDDMWQSDAWTNLLRIPLHAAE

MGAILITTRNNIVALEIRVDHTYRVDLMSTDVGWELLWKSMNISESIELQALQDVGIEIV

RKCGCLPLAIKVIARVLASKEQTENEWKKILSKNAWFMNKLPNDLRGALYLSYDELPRHL

KQCFLYCSVYPEDANIYRDDRTRMWIAEGFIEDHGGQLLEETADEYYYELIHRNLLQPDG

LYYDHSSCKMHDLLRQLACYLSREECFIGNPESLVGNTVSKLRRVSVVTDKNMVMLPSMD

EVQCKVRTWKTSYEKTLRVDNSFFKRFPYLHVLDLTDSFVPSIPRCIGNLIHLRLLDLDG

TNVSCLPESIGNLKNLQILNLERCVALHSLPSAITQLCNLRRLGLNYSPIYQVPKGIGKL

EFLNDVEGFPVYGGSSNTKMQDGWNLEELAYLYQLRRLHMIKLERAAYRTIYPLLTDKGF

LKFLYLWCTERTDEPYTEKDFSNIEKIFEQLIPPCNLEDLAIVKFFGRQYPFWIDSTHLA

YVKSLHLFNCKFCMHLPPVGQLPNLKYLKIEGAAAVTIIGPEFAGHRASNLGRTVAFPKL

EELLIRDMPNWEEWFFIDEATSTAKERVDDGDSAMPKEKALPPRMQILSRLRRLELSGCP

KLKALPRQLAQINSLKEIELRWASSLKVVENFPLLSEMLLIATFQALEKVSNLPQVRELR

LQDCPNLRLVEDLSTLEQLWLYEDMHEVSTLWVPGLQQQCRQHHGEDLDVYNWT*

>KAS_RG018

MATILGSLVGSCVNKLQGIITEEAILILGVKEELRKLQERMKQIQCFINDAERRGMEDSA

VHNWISRLKDVMYDADDIIDLASFEGNKLLNGHSSSPRKTTACSALSPLSCFSNIRVRHE

IGDKIRMLNRKLAEIEKDKIFATLENTQPADKGSTSELRKTSHIVEPNLVGKEIVHACRK

LVSLVVAHKEDKAYKLAIVGTGGVGKTTLAQKIFNDKKLEGRFDHRAWVCVSKEYSMVSL

LTQVLSNMKIHYEQNESVGNLQSKLKAGIADKSFFLVLDDVWHYKAWEDLLRTPLNAAAT

GIILVTTRDETIARVIGVDRTHRVDLMSADVGWELLWRSMNIKEEKQVKNLRDTGIEIVR

KCGGLPLAIRAIAKVLASLRDQTENEWRQILGKNAWSMSKLPDELNGALYLSYEVLPHQL

KQCFLYCALFPEDANIFCGDLTRMWVAEGFIDEQEGQLLEDTAERYYHELIHRNLLQPDG

LYFDHSWCKMHDLLRQLASYLSREECFVGDPESLGTNTMCKVRRISVVTEKDIVVLPSMD

KDQYKVRCFRNLSGKSARIDNSLFKRLVCLRILDLSDSLVHDILGAIGNLIYLRLLDLDR

TNICSLPEAIGSLQSLQILNLQECESLRRLPLATTQLCNLRRLGLAGTPINQVPKGIGRL

KFLNDLEGFPIGGGSDNTKMQDGWNLEELAHLSQLRCLDMIKLERATPCSSTDPFLLTEK

KHLKVLNLHCTEQTDEAYSEENARNIEKIFEKLTPPHNLEDLFVGNFFGCRFPTWLGTNH

LPSVKSVILVYCKSCVHLPPIGQLPNLKYLKINGASAITKIGPEFVGCWEGNLRSTEAVA

FPKLEVLVINDMPNWEEWSFVEEEEELQEEETTAAAKEGGEDGTAASKPKGEEAPSPTPR

SSWLLPCLNKLILVNCPKLRALPPQLGQQATNLKEFSIRRARCLKTVEDLPFLSGFLQVE

GCEGLERISNLPQVRELRVNVCPNLRHVEELGGLEQLLLDEGMQEISQLWVPGLQEQHRQ

LHGDEHELEVIEWL*

>KAS_RG019

MATILGSLVGSCVNKLQGIITEEAILILGVKEELRKLQERMKQIQCFINDAERRGMEDSA

VHNWISRLKDVMYDADDIIDLASFEGNKLLNGHSSSPRKTTACSALSPLSCFSNIRVRHE

IGDKIRTLNRKLAEIEKDKIFATLENTQPADKGSTSELRKTSHIVEPNLVGKEIVHACRK

LVSLVVAHKEDKAYKLAIVGTGGIGKTTLAQKVFNDQKLKGTFNKHAWICVSQDYTPVSV

LKQLLRTMEVQHAQEESAGELQSKLELAIKDKSFFLVLDDLWHSDVWTNLLRTPLHAATS

GIILITTRQDIVAREIGVEEAHRVDLMSPAVGWELLWKSMNIQDEKEVQNLRNIGIEIVQ

KCGGLPLAIKVTARVLASKDKTENEWKRILAKNVWSMDKLPKEISGALYLSYDDLPLHLK

QCFLYCIVFPEDWTLKRDELIMMWVAEGFVEVHKDQLLEDTAEEYYYELISRNLLQPVDT

YLDQSRCQMHDLLRQLACYLSREECHIGDLKPLVDNTICKLRRMLVVGEKDTVVIPFTGK

EEIKLRTFTTDHQLQGVDNTFFMRLTHLRVLDLSDSLVQTIPDYIGNLIHLRMFDLDGTD

ISHLPESIGSLQNLQILNLQRCKSLHRLPLATTQLCNLRRLGLDGTPINQVPKGIGIMKF

LNDLEGFPIGGGSDNTKMQDGWNLEELAYLPQLRQLGMIKLERGTPRSSTDPFLLTEKKH

LKVLNLHCTKQTDEAYSEENARNIEKIFEKLTPPHNLEKLVIVNFFGCRFPTWLGTNHLP

SVKYVVLIDCKSCVHLPPIGQLPNLKYLKINGASAITKIGPEFFGCWEGNLRSTEAVAFP

KLEELVIVDMPNWEEWSFVEEEEVQEEEAAAAAKEGGEDGTAASKQKGEEAPSSRSSWLL

PCLTRLQLVGCPMLRALPPQLGQQATNLKEFFIRYTSCLKTVEDLPFLSGYLLVDGCEGL

ERVSNLPQVKELLVNVCPNLRHVEELGGLEQLWLDEGMQEISSLWVPRLQEQHRQLHGDE

HELEVTEWL*

>KAS_RG021

MATILDSLVGSCANKLKEIITEEVILILGIQEELAELQRKTELIHCCISDAEARRMEESA

VDNWLARHEVAMKIRSLNRKIENISKDRVFLTLKSTVPTGSSSVLRVRKSSHLLEPNIVG

KEIIHACRKMVDLVLEHKGRKLYKLAIVGTGGVGKTTLAQKIYNDRKIKGSFNKKAWVCV

SKVYSEASLLRELLRIMEVHHDQDESIGELQSKLEIAIKETSFFLVLDDMWQSDAWTNLL

RIPLHAAEMGAILITTRNNIVALEIGVDHTYRVDLMSTDVGWELLWKSMNISESIELQTL

QDVGIEIVRKCGCLPLAIKVIARVLESKEQTENEWKKILSKNAWFMNNLPNDLRGALYLS

YDELPRHLKQCFLYCSVYPEDANIYRDDLTRMWITEGFIEDHGGQLLEETADEYYYELIH

RNLLQPDGLYYDHSSCKMHDLLRQLACYLSREECFVGNPESLVGNTVSKLRRVSVVTDKN

MVMLPSMDEVQYKVRTWKTSYEKTLRVDNSFFKRFPYLRVLDLTDSFVPSIPGCIGNLIH

LRLLDLDGTNVSCLPESIGNLKNLQILNLERCVALHSLPSAITQLCNLRRLGLNYSPIYQ

VPKGIGKLEFLNDVEGFPVYGGSSNTKMQDGWNLEELAYLYQLRRLHMIKLERAAYRTTY

PLLTEKEKDFSNIEKIFEQLIPPCNLEDLAIVKFFGRQYPFWIDSTHLAYVKSLHLFNCK

FCMHLPPVGQLPNLKYLKIEGAAAVTIIGPEFAGLRASNLGRTVAFPKLEELLIRDMPNW

EEWFFIDEATSTAKERVDDGDSAMPKEKALPPRMQILSRLRRLELSGCPKLKALPRQLAQ

INSLKEIELRWVSSLKVVENFPLLSETLLIATCQALEKVSNLPQVRELRVQDCPNLRLVE

ELGTLEQLWLYEDMHEVSTLWVPGLQQQCRQCHGEDLDVYNWT*

>KAS_RG022

MATIVDTLVGSCINKLQAIITDKTILILGVKDELEELQRRTNVIRSSLQDAEARRMEDLV

VEKWLDQLRDVMYDVDDIIDLARFKGSVLLPDYPMSSSRKSTACSGLSLSSCFSNIRIRH

EVAVKIRSLNKKIDNISKDEVFLKLNRRHHNGSGSAWTPIESSSLVEPNLVGKEVIRACR

EVVDLVLAHKKKNVYKLAIVGTGGVGKTTLAQKIFNDKKLEGRFDHHAWACVSKEYSRDS

LLRQVLRNMGIRYEQDESVPELQRKIKSHISNKSFFLVLDDVWNSEAWTDLLSTPLHAAA

TGVILITTRDDTIARVIGVDYTHRVDLMSADVGWELLWRSMNINQEKQVQNLKDIGIEIV

RKCGGLPLAIRVIATVLASQEQTENEWRRILGKNAWSMSKLPRELSGALYLSYEVLPHQL

KQCFLYCALFPEDETILRDDLTRMWVAEGFIDEEKGQLLEDTAERYYYELIHRNLLQPDG

LYFDHFSCKMHDLLRQLACYLSREECFVGDVESLGTNTMCKVRRISVVTEKDMMVLPSIN

KDQYKVRTYRTSYQKAPQVDSSLFEKLTYLRVLNLTNSHVQRIPNCIENMIHLRLLDLDG

TDISHLPESIGSLQNLQILNLESCKSLHRLPLAITQLCNLRRLGLYGTPINQVPKGIGIM

KFLNDLEGFPIGGGSDNTKMQDGWNLEELAHLSQLRCLDMIKLERATPCSSTDPFLLTEK

KHLKVLNLHCTEQTDEAYSEENARNIEKIFEKLTPPHNLEDLFVGNFFGCRFPTWLGTNH

LSSVKYIALIDCKSCVHLPPIGQLPNLKYLRIKGASAITKIGPEFVGCWEGNLRSTEAVA

FPKLEALVIKDMPNWEEWSFVEEEEVQEEEASAAGKEGGEDGTAASKQKGKEAPSPTPRS

SWLLPCLKKLYLEECPKLRGLPRQLRQQATNLKDLQLREADCLKKVEDLRFLCFLLVEGC

EGLERLSNLPQVRDLRVTRCPNLRHVEELGGLEWLWLDDGMLEISQLWVPGLQEQHRQLH

GDELEINDWPRT*

>KAS_RG023

MATIVDTLVGSCINKLQAIITDKAILILGVKDELEELQRRTDLIRSSFNDAEARRIEESA

VEKWIGQIRDVMYDVDDIIDLARFKGSVLLPDYPTSSSRKSTACSGHSLKSCFSNIPRTP

IESSALIQPDLVGKEAIYACREVVDLVLAHKENKDFKIAIVGTGGVGKTTLAQKIFNDKK

LERRFDKHAWVCVSKEYSRDSLLRQVLRNMGIPHDKDELVGELQSNLASNIQGKSFFLVL

DDVWHSEAWEDLLRTPLHAAATGVVLVTTRDDTIARVIGVDHTHRVNLMSADVGWELLWR

SMNIKEEKHVQNLKDIGIEIVCKCGGLPLGIRVVAKVLATHDQTENEWRKMLENSAWSMS

KFPHELRGALYLSYDALPHHLKQCFLYCALYPEDVILHRDYLIWGWVAEGFIEKQEGQLL

EDTVEKYYYELINRNLLQPDGSYIDHITCKMHDLLRQLACYLSREECYVGDPESLGSNSM

CKVRRISVVTEKDMVVLPSMDKDQYKVRSFTYLLYGTSQRIDNSLFKRLVCLRILDLSDS

LLHNIPGEIGHLIHLRLLDLNRTDISSLPESIGSLQNLQILNLLDCESLHSLPLATTQLR

NLRRLGLGGTPINNVPKGLGRLKFLNDLVGLPVSHGSGNTKMQDGWNLKELAHLSQLRRL

LLINLEKATPSSSTDSLLLIDKVHLKYLYLCCTVPADEEYSEEDVSNVEKIFDQLEPPHD

VQEEEWVFIEEEFEEEEEAAEEGEEDGAASKQKGKEALSPRSSWLLPCLKKFDIVNCPKL

RALPPQLGQQATSLKQLIIEYAGCLKMVEDLWFLSDALQIQACEGLERVSNIPCVRKLYV

NFCPNLRCVEELGSLEQLWLDEDMQEISLLWVPGHQEQHRQHHEDELEVIEWFASPIE*

>KAS_RG026

MATIVGTLVGSCVNKLQGIITEEAILILGVKDELEELQRRTDLIRYSLQDAEARRMKDSA

VQKWLDQLRDVMYDVDDIIDLARFKGSVLLPDYPMSSSRKSTACSGLSLSSCFSNIRIRH

EVAVKIRSLNKKIDNISKDDVFLKLSRTQHNGSGSAWTPIESSSLVEPNLVGKEVIRACR

EVVDLVLAHKAKNVYKLAIVGTGGVGKTTLAQKIFNDKKLEGRFDHRAWVCVSKEYSMVS

LLTQVLSNMKIHYEQNESVGNLQSKLKAGIADKSFFLVLDDVWHYKAWEDLLRTPLNAAA

TGIILVTTRDETIARVIGVDRTHRVDLMSADVGWELLWRSMNIKEEKQVKNLRDTGIEIV

RKCGGLPLAIRAIAKVLASLQDQTENEWRQILGKNAWSMSKLPDELNGALYLSYEVLPHQ

LKQCFLYCALFPEDATIFCGDLTRMWVAEGFIDEQEGQLLEDTAERYYYELIHRNLLQPD

GLYFDHFSCKMHDLLRQLACYLSREECFVGDVESLGTNTMCKVRRISVVTEKDLMVLPSI

NKDQYKVRTYRTSYQKALQVDSSLFEKLTYLRVLDLTNSHVQRIPNYIENMIHLRLLDLD

GTDISHLPESIGSLQNLQILNLQRCKSLHRLPLATTQLCNLRRLGLYGTPINQVPKGIGI

MKFLNDLEGFPIGGGSDNTKMQDGWNLEELAHLSQLRCLDMIKLERATPCSSRDPFLLTE

KKHLKVLNLYCTKQTDEAYSEEGISNVEKIFEKLTPPHNLEKLVIVNFFGCRFPTWLGTA

HLPSVKSVILTNCKSCVHLPPIGQLPNLKYLRIEGASAITKIGPEFVGCWEGNLRSIEAV

AFPKLEVLVINDMPNWEEWSFVEEEEEVQEEETAAAAKEGGEDGTAASKQKGEEAPSPTP

RSSWLLPCLNELVLVNCPKLRALPPQLGQQATNLKKLFIREAECLKTVEDLPFLSGCLQV

EGCEGLERISNLPQVRELRVNVCPNLRHVEELGGLEQLLLDEGMQEISQLWVPGLQEQHR

QLHGDEHELEVIEWL*

>KAS_RG027

MATIVGTLVGSCVNKLQGIITEEAILILGVKDELEELQRRTNVIRYSLQDAEARRMKDSA

VEKWLDQLRDVMYDVDDIIDLARFKGSVLLPNYPMSSSRKYTACSGLSLSSCFSNSRIRH

EVAVKIRSLNKKIDNISKDDVFLKLSRTQHNGSGSAWTPIESSNLVEPNLVGKEVVHACR

EVVDLVLAHKAKNVYKLAIVGTGGVGKTTLAQKIFNDKKLEGRFDHRAWVCVSKEYSMVS

LLAQVLSNMKIHYEQNESVGNLQSKLKAGIADKSFFLVLDDVWHYKAWEDLLRTPLNAAA

TGIILVTTRDETIARVIGVDRTHRVDLMSADVGWELLWRSMNIKEEKQVKNLRDTGIEIV

RKCGGLPLAIRAIAKVLASLQDQTENEWRQILGKNAWSMSKLPDELNGALYLSYEVLPHQ

LKQCFLYCALFPEDATIFCGDLTRMWVAEGFIDEQEGQLLEDTAERYYHELIHRNLLQPD

GLYVDHSRCKMHDLLRQLASYLSREECFVGDPESLGTNTMCKVRRISVVTEKDIVVLPSM

DKDQYKVRCFTNLSGKSARIDNSLFKRLVCLRILDLSDSLVHDIPGAIGNLIYLRLLDLD

RTNICSLPEAIGSLQSLQILNLQGCESLRRLPLATTQLCNLRRLGLAGTPINQVPKGIGR

LKFLNDLEGFPIGGGNDNTKIQDGWNLEELAHLSQLRCLDMIKLERATPCSSRDPFLLTE

KKRLKDLNLHCTEQTDEAYSEEGISNVEKIFEKLAPPHNLEDLVIGNFFGRRFPTWLGTN

HLSSVRSVILIDCKSCVHLPPIGQLPNLKYLRINGASAITKIGPEFVGCWEGNLRSIEAV

AFPKLEVLVINDMPNWEEWSFVEEEEEVQEEETAAAAKEGGEDGTAASKQKGEEAPSPTP

RSSWLLPCLNELGLVNCPKLRALPPQLGQQATNLKKLFIRYTSCLKTVEDLPFLSGFLQV

EGCEGLERISNLPQVRELRVNVCSNLRHVEELGGLEQLLLDEGMQEISQLWVSGLQEQHR

QLQVEGCVGLERVSNLPQVRKLFVNVCLNLRHVEELGGLEQLLLAKGMQEISQLWIPGLQ

EQHRQLHGDEHELEVIEWL*

>KAS_RG028

MATILDSLIGSCAKKLQEIITEEAILILGVKEDLRELQEKMEQIRCFISDVERRGMEDSS

IHNWISRLKDAMYDADDIIDLASFEGSKLLNGHSCSLRKTIACSGLSLLSCFSNIRVHHE

IGNKIRSLNRKLEEIAKDKIFVTLENTQSSHKDSTSELRKSSQIAESNLVGKEILHASRK

LVSQVLTHKEKKTYKLAIIGTGGIGKTTLAQKVFNDEKLKQSFDKHAWICVSQDYSPASV

LGQLLRTIDAQCKQEESVGELQSKLESAIKDKSYFLVLDDVWQSDVWTNLLRTPLYAATS

GIVLITTRQDTVAREIGVEEPHHIDLMSPAVGWELLWKSINIEDEKEVQNLRDIGIEIVQ

KCGGLPLAIKVIARVLASKDKTENEWKKILANYVWSMDKLPKEIRDCTIRRDDLIRLWVA

EGFVEVHKDQLLEDTAEENYYELISRNLLQPVDTSFDQSKCKMHDLLRQLACHLSREEYY

IGDPTSLVDNNMCKLRRILAITEKDMVVIPSMGKEEIKLRTFRTQPNPLGIEKTFFMRFT

YLRVLDLTDLLVEEIPDCVGYLIHLRLLDLGGTNISCLPNSIGALKNLQMLNLQWCKSLY

SLPSTFTRLSNLKRLGLDFTPINQVPRGIGRLEFLNDLEGFPVGGGSDNTKMQDGWNLQE

LSHLSQLRRFDLNKLERATPRSSTDALLLTDKKNLKSLHLCCTERTDEAYSEEAISNVEM

IFEQLSPPRNLEDLMIVLFVGRRFPTWLSTSLLSSLAYLKLKDCKSCVHLPPIGQLPNLK

YLRIKGASAITKIGPEFVGCWEGNLRSTEAVAFPKLKLLAIEDMPNWEEWSFAPRR*

>KAS_RG029

MATIVDTLVGSCINKLQAIITDKAILILGVKDELEELQRRTDLIRYSLQDAEARRMKDSA

VQKWLDQLRDVMYDVDDIIDLARFKGSVLLPNYPMSSSRKSTACSGLSLSSCFSNIRIRH

EVAVKIRSLNKKIDNISKDDVFLKLSRTQHNGSGSAWTPIESSSLVEPNLVGKEVVHACR

EVVDLVLAHKAKNVYKLAIVGTGGVGKTTLAQKIFNDKKLEGRFDHRAWVCVSKEYSMVS

LLTQVLSNMKINYEQNESVGNLQSKLKAGIADKSFFLVLDDVWHYKAWEDLLRTPLNAAA

TGIILVTTRDETIARVIGVDRTHRVDLMSADVGWELLWRSMNIKEEKQVKNLRDIGIEFV

RKCGGLPLAIRAIAKVLASLQDQTENEWRQILGKNAWSMSKLPDELNGALYLSYEVLPHQ

LKQCFLYCALFPEDATIFCGDLTRMWVAEGFIDEQEGQLLEDTAERYYHELIHRNLLQPD

GLYFDHSMCKMHDLLRQLASYLSREECFVGDPESLGTNTMCKVRRISVVTEKDIVVLPSM

DKDQYKVRCFTNLSGKSARIDNSLFKRLVCLRILDLSDSLVHDIPGAIGNLIYLRLLDLD

RTNICSLPEAIGSLQGLQILNLQGCESLRRLPLATTQLCNLRRLGLAGTPINQVPKGIGR

LKFLNDLEGFPIGGGNDNTKIQDGWNLEELAYLPQLRQLGMIKLERGTPRSSTDPFLLTE

KKHLKVLNLHCTEQTDEAYSEENARNIEKIFEKLTPPHNLEDLFVGNFFGCRFPTWLSTS

QLSSLTYLKLKDCKSCVHLPLIGQLPNLKYLRINGASAITKIGPEFVGCWEGNLRSTEAV

AFPKLEALVIEDMPNWEEWSFVEEEEVQEEEAAAAAKEGGEDGTAASKPRSSWLLPCLTR

LELDDCPKLRALPPQLGQQATNLKELDIRRARCLKTVEDLPFLSGCLLVERYEGLERISN

LPQVRELRVNVCPNLRHVEELGGLEQLLLDEGMQEISQLWVPGLQEQHRQLHGDEHELEV

IEWL*

>KAS_RG030

MATILDSLIGSCAKKLQEIITEEAILILGVKEDLRELQEKMEQIRCFISDVERRGMEDSS

IHNWISRLKDAMYDADDIIDLASFEGSKLLNGHSCSPRKTIACSGLSLLSCFSNIRVHHE

IGNKIRSLNRKLEEIAKDKIFVTLENTQSSHKDSTSELRKSSQIAESNLVGKEILHASRK

LVSQVLTHKEKKTYKLAIIGTGGIGKTTLAQKVFNDEKLKQSFDKHAWICVSQDYSPASV

LGQLLRTIDAQCKQEESVGELQSKLESAIKDKSYFLVLDDVWQSDVWTNLLRTPLYAATS

GIVLITTRQDTVAREIGVEEPHHIDLMSPAVGWELLWKSINIEDEKEVQNLRDIGIEIVQ

KCGGLPLAIKVIARVLASKDKTENEWKKILANYVWSMDKLPKEIRGALYLSYDDLPQHLK

QCFLYCIVYPEDCTIRRDDLIRLWVAEGFVEVHKDQLLEDTAEEYYYELISRNLLQPVDT

SFDQSKCKMHDLLRQLACHLSREECYIGDPTSLVDNNMCKLRRILAITEKGMVVIPSMGK

EEIKLRTFRTQPNPLGIEKTFFMRFTYLRVLDLTDLLVEEIPDCVGYLIHLRLLDLGGTN

ISCLPNSIGALKYLQMLNLQWCKSLYGLPSTITRLSNLRRLGLDFTPINQVPRGIGRLEF

LNDLEGFPVGGGSDNTKMQDRWNLQELSHLSQLRRLDLNKLERATPRSSTDALLLTDKKH

LKSLHLCCTEPTDEAYSEEAISNVEMIFEQLSPPRNLEDLMIVLFFGRRFPTWLSTSLLS

SLAYLKLKDCKSCVHLPPIGQLPNLKYLRIKGASAITKIGPEFVGCWEGNLRSTEAVAFP

KLKLLAIEDMPNWEEWSFVEEEEEKEVQEEEASAAAKEGGEDGTAASKQKGEEAPSPTPR

SLWLLPCLTKLQLVECPKLWLSKNMQKISSLWVPGLEEQHRQLHGDEHKLEVNEWF*

>KAS_RG031

MEAALGPLISALITVNLPRFLDWLGEEDGQEFEALKNNVDTIRRWFDAIDLLIQQHRLIV

SKDSRNFRKEWILPLRHLANKIEDCVDQLQATKTKKARDFFEKRIDELSKESKEKHVELT

KYFSNPPQKKNDAPDPPAAHEAAPKAHHEMVGIKGVVKELRELVVRQSDCQSERKLRVIC

IVGFGGIGKTSLVSEVIDEFPHTWVSASGKSAQQVVNELRDKLRNGLSGANRKILCHLIS

SSILEEQPPDEDEPVGRVIRYYDKGKGIDGASLSSNLAQVMNKGVHNKDIHMLADNDSGK

VLEIKNACPGTPQFVDTNSALNKKKDASLEEAREFYKGKGAADIASTSNHNSEVMNDIGV

LGASTKHVGRYLIVIDDVQERAVLEDIISSNVLRHMEVDSTIIATTTIQSVAYCDSGSGE

HQVYKLKKLTSPDDQKSLFLQVAQMQQTDLSSTVEENTLKKVIGRCDGLPLALVSIAEDV

RGDPSSNRCEKALRCNVCDDHHISNGPLGRMQRVLFYSYHNLDSDIIKDFLLYFGMFPRG

HPVKRDSLIRRWMAEGLNITSDDGSSNYLNCILEALIDRNIIQPIPESNINVVSSNIKEN

VKRCQLPGMILEYISYQSNSEQFMHKWLYHEDPPKEYVRRLTVHNYKDDRNITRAFPNLR

TLAVFPTKDEKDEGAAVVGLKANFADYKFLRVLDLEECDGLNNKHLKEICDQLLLLKYLS

LGGSITEVPREIARLKCLQTLDLRRSCPNIMVPIEVFYLLPELKHLLGMFQILAQDIFLV

RKLEKKLSDGCQLQTLAGFVIGKRRRISWLMIHMTSPLRKLKIWCKSNADKANLTHVSET

IQKFIREGISMIGVDRSLSIHWKEGCSTDFLDFSNEALGILTSLKLKGQLQNFPQFVSRL

EGIKKLCISSTGLRGNTIITGVRGLKYLEYLKLVDDNLVELVIKSKGESGQYYFHSLKQI

CLVSYPNLPRIEIQDGALKFVISLHLHSSQLPNPSFVIGVKHLKKLEDITLISQGDGDHN

KENMDLWKEEARLHPNRPNVWEIKKPRHGERKW*

>KAS_RG035

MGSVITVLGWLLSPIISLLVNRFISYLFDASPKIQELEIQTVPKLEQMLRKIEEERMHRK

AKKERSAVQNLDTLAKLVKSALYEAEDVLDLIAYHQIEKDVIGDDEPQGRSSKWHPHIDD

AIHACKTSWTGRCITTLLEWAQSLYRSLRSRSAALLPISCSRCCGSASDSLLERLSCLSR

QFDFIRCCQSLFIWSVNWFEVARSYRDWFYDATGITATGYQLEDGTAVYSFMPAIARWKL

RKRIEKLENTVTNVEKSPYLTQTSSGAWNDIVNMNRRSITSSSTRKVFGRDRERDMIRSM

LREDDSLPSSSSRKCYSVICIYGIPGSGKTTLAQYVCEYEKEDKDRYFDTVMLIYVSKTY

RLEDIFRDMLEEITQSRHSEISDCRGLEAKLVENLRGKRFLLVLDDLWVNDENHEKLLSP

LNVGKSGSRILVTAQSKEAALGSNRLIPISDLEEEQYFSMFMHYALDSTIFDDREYIPIG

RKIAKKLNRSPIAAVTVAGQLWRNPDIRFWQTTANLDVLNKTKGALWWSYNQLVVDVRRC

FQYCSIFPRRYELERDNLVRMWIAQGFVKDNDGNNEDVEDVGQDYFHDLHSCSFLQLKRK

APSDISTGEYFTVHDMFHELAKTIAGSDCVKIEKSITEHLPKHVRHLCIESYSEILFPEK

ILELKNLRTLIMCYSVEGMNQDDFERVLKKLTKLRVVHLDLRHLSRVPPCIGGLKHLRYL

GIMSPPPHSLILPAEFSKLYHLQELSVNPNTRLHCPSQLKIANLINLRYMLTWYGLNIPD

VGKLTSLRALYHFYVRKEKGYEIQQLEHLNNLRGKLFIDCIENVQSKEEAVRARLSDKVY

LTELTLRWGGTDERCSKKALESYKKLFFPPVTEIKQHQPPELQEEVLEGLRPPSGITVLC

IRDYGGVIYPSWLTGDGCDKEQEQDRPALQNLMFWSCKGSSDPPKIGEFFTCLHTLSVTD

CSWNYLPVKLCRLKTLRELIVQECPNMMTLPKLPQSLKSIVISGCHPSLADTCLTPGHPN

WRRIKHIDQQIIR*

>KAS_RG036

MEGAMFNLPGRLDGLLLRHDSILPKGAEEEIPLIMQDLEKIISILHGHCSEPKLEDHAMV

MRCWMKEVRELSYDIEDCIDQYEHYSTATRSRPGPNIRHRKFNRRRGNKIPWVPQKLKQR

LWMANKIREFSLRVQEALQRHAMYNNLGGIAGTASTTSGDVCSATPLHPTQTQFREHVDN

VRSVSVDADGMEGALNDLNKLKNLLAGIPTASLVQFKEHRNKLRGIHTDIEAILTKLKNI

TTTSTASRGDASSTSSRQPTWFMESTCHVGIDAAMNKLENLLDVCGEEKLKVVSIVGVGG

VGKTTLANKLYRKLRWQFECRAFVRTSQKTDMTRLLINILSQVRPHQSPDNWKVHSLISS

IRSYLQDKRFLIVIDDLWATSTWDIIKCALPEGNKSSRILTTTEIEDLALQSCSYDLKFI

FKMKAFCEDDSRKLLFNIVFGSHSKCPPEVSETLYDIVRKCGGLPLAIVTVASLLASQLD

KQEQWDYINKSLGFSLMANPTLEGMKQLLNLCYNNLPQHLKACMLYLSMYQEDHIIWKDD

LVSQWIAEGFICATEGHDKEEISRAYFDELIGRKIIQPVHINDSGEVLSCVVHHMVLNFV

TYKSIEENFIIAIDHSQATIRFADKVRRLSIHFGNVEDAPPPTNMRLSQVRTVAFFGVLK

YMPFILEFRLIKVLVLHFLGDEDSIGIFDLTKISELIRLRYLKVTSNVTVKLPTQMQGLQ

YLETLKIDGKISEVPSDIIHLPGLLHLTLPAKTSLPNGIAHVTSLRTIGYFDLSCNSVEN

LWRVGELTNLRDLQLTYSEIHSDNLKDNMKYLGSILGKLRNLISITLSPPGSSCPDTLYI

DRDTKTRINVDGWSSVSSPPALLQRFELLPCVCIFSNLPNWIGQLGNLCILKIGIREVTS

NNIDVLGVLPELTVLSLYVHTKPAERIVFDNAGFSILKYFKFICSVACMKFEMGAMPSLR

KLKLGFDVHRADQHDIIPVGIEHLSGLEEISAKIRVACSAHDHCKRFAESALSIAFMMHP

GRPSVNIRCVDWTFDGKDDDNVRTQEEEHRTLQKQHHTAKEGSNEKSPVKQRDPREGAHK

SVDGRETLRRIRVKVSSTVDDGFSWVKYGQKDILGTMYPRSYFRCIHRQTKGCLATKQVQ

PTDDDHQILDVIYYGEHTCDQSARSDDRQLKSSRPAASSNLQAPQQPGLEQSRPAAKRRR

KTVRWKTQVRVSSVQDVGPLDDGYSWRRYGLKDILGAKYPRSYFRCTHRNTQGCVATKQI

QRRDGDPLLFDVVYHGDHTCSERASLNEQVTWPRSSASSTEQSSTITYTAAAGSVEDDEE

GVTSATNFLSMDDMLDLGGGDVIDMDFPSFDFDAIDALLLG*

>KAS_RG037

MESAAASAFWEAIMGKLFMVLESQYNKHKALEKEISSLQQEFRMIAAVMDDQLHSMGRSE

ARTAVARLHSEEMLDLEHDIEDCVDRFMHLLTCKHHRGGVRQMAHEVKKVKIRSSFSEEI

QKLRRRLSEVRQRVVNINSPIACQSAGSSSSTPYHAAHSLVGIREPMEELLSLLDEVEGE

PKQLRVISIVGFSGLGKTTLAKAVYDSPHAKDKFCLRAWITADGSPETSNWMKEILRGVL

QQVRPGDAMDVDGQHLEASLKEYLKDKRYLIIIDDIRMDQWRIISSAFENNGTGSRILLT

TTIQSVANRCSHGNGYVYQMNTLGKEDSEELAFSVLRSPELENHSESLLGKCDGLPLALV

SVSDYLKSSTEATGELCAKLCRDLGSHLTGNHGHDNFSELRRKKVLIRRWLAEGYARSDD

PWRSEEDTADDNFSNLIDQNIIQPVDTRNNSEVKTCKTHGIMHEFLLNRSLAKRFITTSP

HDPRVGINTTNSRHLSVDAAKQTKCMASDEELSRVRSLTIFGDAGDAISYLHKCNLIRVL

DLQECSDLNDDHLKRICVLSPWHLKYLNLGGNISELPRSIEGLHCLETLDLRETEIKFLP

IEGIMLPHLAHLFGKFMLHKDDLNNVKKISKLLKLFSSNKSNLQTLAGFITDGRKGFLQL

IGHMKKLRKVKIWCRHVEGSSNYIADLSKAIQEFTKAPIDMDRVRSLSIDSEEYCEKFLS

SLDLEPCSEYSKYHLSSLKIHGKLLRLPPFFTSLSGLIDLCITPATLTQDHLSALINLNR

LLYLKLIADKLENLEIKHGALLSLRRLCFVVKSVALAQPKIEQGALPNLVSLQLLCQGLV

GLSGIEIRHLKNLKEVTIDSGVTAQTRQDWEQAAKNHPNRPRVLLLRKVDPVESEEPGRP

CAIRGRGKSSIGQESSEDGSDSSLKRMRLAEPSSSSQLQVTGHPHPVVVAATEAASQPSM

ANL*

>KAS_RG039

ARTAVARLHAEEMLDLEHDIEDCVDRFMHLLTCKHNSLSGRTSLVCRVKHEVKKVQSRSS

FSEEIQKLRRRLSEAQQRSFGLHAYGGFQDKSKSPRNPSKGIYTVPLRFGPSLRRRASAP

LRQKPGQYLEVNLEQLQQPSGCKPAESRSSTSSSTPCRAACSPVGIGEPMEELLSLLDEV

EGEPEQVRVISIVGFGGLGKTTLAKAVYDSPRAKDRFSHRAWVTAGGSPETTDWMREILR

DVLQQVRPDNAMDVDAQHLEASLREYLKDKRYLIVIDDIDVDQLRIIESIFPDNGTGSRI

IVTTDNQQVANTCSHGNGYVYQMKTLGKEDSKKLAFSGLRSVEPGQGPASLLAKCDGLPL

ALVSVSDYLKSSSEPTGELCAELCLNLGSDLKEDGHYSFAQLRKVLLDNYDSFSGYTLSC

LLYLGIFPNNRPLKKKVVIRRWLAEGYARSDDPRRSEEYTADKNFRKLIDRNIIQPVDTR

NNSEVKTCKTHGIMHEFLLNKSLAQRFIGTSLHDHPRVGINTSNARHLSVDAAKQTECVA

SDEELSRVRSLTIFGDAGDTISCLRKCKLLRVLDLQECNGLNDDHLKHIYELWHLKYLSL

GGYINEFPRSIQGLHCLETLDLRRTEIKFLPIEAIMLPHLAHLFGKFMLHKDDLKNAKKM

SKLQKFFSSNKSNLKTLAGFITEEGKEFLQLIGHMKKLRKVKIWCKHVEGSSNYIADLSK

AIQEFTKTPIDMDRVRFLSLDSEECSENFLSSIHLEPCSEDYKYTLKSLKLHGNLLQLPL

FVTLLSGLIELCISSATLTQEHLSALTNLNSLLYLKLVADKLENFEIKLGAFLSLRRLCF

VGKNAASALPKFEQGAMPNLVSLQLLCQGLVGLSGIEIRHLKHLKEVTIDSRVTAQTRQD

WEQAAKNHPNRPRVLLLGEVHSVESEEPGRPMEKRRICVGQASSEDERDSSLKRMRLSDP

SSSRLQVIGHPHPVVVTATEAASQPSMAN*

>KAS_RG040

MFNLPGRLDGLQLRHGSILPKGAEEEIPLIKQDLEEIISILHGHCSEPKLEDHAMVVRFW

MKEARELSYDIEDCIDQYEHAATTTLSRTGPNIRHYKFNRRRGNKIPWVPQKLKQRLWMA

NKIREFSLRTQEALQRHAMYNNLGGIARTAATTSGDVFSATPWYPRHTQLREHVDNVRSV

SIDADGMEAALNDLNKLKNLLAGIPTASLVQFREHGDKVLGIHTDMEAVLNKLKNILPGI

TTTTTSRGDVSPTSSRQPTRFMESICHVGIDAAMNKLENLLDVCGEEKLKVVSIVGVGGV

GKTTLANKLYRKLRWQFECRAFVRTSQKTDMRRLLINILSQVRPHQSPDNGKVHSLISSI

RTHLQDKRYLIIIDDLWATSTWDVIKCALPDGNSSSRILTTTEIEDLALQSCSYDLKFIF

KMKPFGEGDSRKLFFSIVFGSHSRCPPEVSETLYDIVRKCGGLPLAIVTVASLLASQLEK

QEQWDYINKSLGYSLMANPTLEGMKQLLKLSYNNLPQHLKACMLYLSMYQGDHIIWKDDL

VSQWIAEGFIRAAEEYDKEEISRAYFGELVGRKIIQPVHIDDSGEVLSCVVHHMVLNFVT

YKSIEENFIVAIDHSQTTVRFSDKIRRLSIHFGNVEDATPPTNMRLSQVRTVAFFGVLKY

MPSIMEFRLIKVLVLNFLGDENIIGIFDLTKISELVRLRYLKVTSNVTLKLPTKIQGLQY

LETLKIDGKIGAIPSDIIHLPGLLNLSLPAKTNLPSGIVHMTSLCTLGYFDLSCNSEENL

QSLGQLTNLRDLQLTYSAVHSDILKNNMQCLGSILGKLGNLKSITLSPAGSSYASNMHID

SGCRIFVDGWSSMSSVPSLLQRLELLPGVCIFSGLPNWIGQLRNLCILKIGTREVTSNDV

VVLRGLPALAALSLYVLTKPTERIVFDNVGFSILKYFKFRCIVAWMKFEVGAMPNLRKLK

LGFDVYRADQYDIIPVGIEHLSVLEEISAKIRVACTTDDLCRRFAESAVTNAIRMHPGHP

SVKIHCVDWTFFYGIDDDNVETLEEELRTLQKQHIMKESSSETSAVLQMDPGEAAQKSAD

RRYGLICHTKSTLALNKTLPIYFEQRHIVSLLDSNTSFLD*

>KAS_RG042

MFNLPRRLEELLCHHGSMLPKGAEEEIPLIKQDLEEIISILHGHSEPKLEDHSMVVRCWM

KEIREFSLRIQDALQRHAMYNNLGGVAGTASTTRGDVCSATPWHPTKTQFREHVDNVRSV

SIDVDGMEAALNDLNKLQNLLAGIPTASLVQFREHADKVRGIHTDIEAILNKLENIPPGI

TTTTTTRGDVSSTSSRQPTRFMESTCHVGIDAAMDQLENLIDVCGEEKLKVVSIVGVGGV

GKTTLANKLYHKLRWQFECWAFVRYLIIIDDLWATSTWDIIKCALPDGNNSCRILTTTEI

EDLALQSCSYDLKFIFKMKPFGEDDSRKLFFSSIFGSHSKCPPEVSETSYDIVRKCGGLP

LAIVIVASLVASHVEKQEQWDYINKSLGYSWMANPTLEGMKQLLNLCYNNLPQHLKACML

YLSMYQEDHIIWKDDLVSQWIAEGFICAIEGHDEEEISRAYFDELLGRKIIQPVHIDDNG

EVLSCVIHHVVLNFVTYKSIEENFTIVIDHSQTTIRFADKIRRLSVHFGNVEDSTPPTNM

RLSQVRSVAYFGVLKYMPFIMEFRLIKVLVLHFLGDEDSTGIFDLTKISELVRLRYLKVT

SNVTLKLPTQMQGLQYLETLKIDGKISAVPSDIIHLPGLLHLTLPAKTNLPNGIVHMTSL

RTIGYFDLSCNSAENLWSLGELTNLRDLQLTCSEIHSDNLKNNMKYLGSILGKLHNLKSM

TLSPVGSSYADTLHIDRATSTGISVIGCSSVSSPPALLQRFDLLPCVCIFSNLPNWIGQL

GNLCILKIGIRKITSDSIDVLGVLPELTVLSLYVHTKPEERIIFKKAGFSILKYFKFRCS

LVWMKFEAGALPNLRKLKLGFDVHREDRYDTVPFGIEHLSRLEEISAKIRIDCTADNLSR

RFAESSFANAIRMHTGCPSINVRCVDWTFDCKDDDNVGTGEEEHRTLEKHHHIVKEGSNE

TTAVLQEDTWERAHKSVNNRYELI*

>KAS_RG043

MQGLQSSCPSCCCCSSLAHVHGVRGGERILEDSHGEAVHDAGERVQQAQRPRARKPLPAA

RPPHDRRRHGRPAAFHGEERRRRTHGRRAAAHRGDARPRARHRGLRRPLLAPPHLQPPQT

RRRRRRVTHELSKVKSRSSFGDEIQKLKKRLREAHQRVLTINPPPILIAGGSSSSAAVAP

PCRAARSPVGIEEDVEELLSMLDEVEGEPVQMRVISVVGFGGLGKTTLAKAVYDEPRAKD

KFRHRAWVAAGGSPEIRGILRDVLQQVRPDDAMDVDGQRLEASLKDYLKDKRYLIVIDDI

GMDQWSIIRGGPRNGPRPGRSMEISAQRKVYMAPGRGPACPGSGSAPCSHGSGYVYQMNT

LGEDDSKRLAFPGFRSPELEQGSASLLGKCDGLPLALVSVSDYLKSSSEPTGELCAKLCR

TLGSHLKEKHGHDNFSELRKVLLDNYDSLSGYALSCLLYLGIFPSNRPLKKKVVIRRWLA

EGYARSDSLRSEEDIADENFSKLIDRHIIQPIDTRNNSEVKTCKTHGIMHEFLLNKSLTQ

RFIATSSHDHPRLGINTTNARHLSVHAGELRECVTSDEELSRVRSLTIFGDASDAISYFR

KCKLIRILDLQEWNNLDDDHLKHICKLWHLKYLSFGGNISELPRSIEGLHCLETLDLRRT

EIKFLPIEAIMLPHLAHLFGKFMLQCLETLDLRRTEIKFLPIEAIMLPHLAHLFGKFMLH

KDDLKNVNKSKLQKFFSSNKSNLRTLAGFITDEGKGFLQLVGHMKKLRKVKVWCKHVAGS

SNYIADLSRAIQEFTRTPIDRDSDRSLSLDCEECSENFLSSIDLEPCSEDSKYHLRSLKL

HGKLLRLPPFVTSLSDKLENIEIKIGAFPSLRRLCFVMKIMTSALTTIEQGALSNLVSLQ

LLCQGLVGLSGIEIRHLKHLKEITIDSAVTVQTRQDWEQAAKNHPNRPRVLLFRKVDPME

SEEPEKPCAIGEKRKLSVARPTGSDGGLDSSLKKMRLSEPSSSRLQVIVHPVVVTATEAA

PQHSFANL*

>KAS_RG044

MEAPTCGLWGAVLNLPGRLDGVLLRHGSILPKGAEEEIPLIKRDLHLMISILNGYYSESP

ELEDATATTMARRRCWTKEVRELSYDIEDCIDHYEHAATAGSAGGRTASGGIPPRRKITR

RRWQRTTPLWIPERLKQRLWMANKIREFSLRTQDALKRHAMFCSSVGGNGIATSTASSST

AATGDASSSSSTICWHTTRFRERDFCVPHVGINVAMNKLEDWLTACDDEDQKRLRVVSIV

GVGGIGKTTLANELYRKLRRQFECWAFVRSSQKPDVRRILISILSQLRLQQPPESWKVHS

LISSIRAHLQDKRYLIIVDDICFISTWDIIKCALPDGTSSSRVLTTTQYDDLAVQSCGYD

TKYVFKMKSLSQHDSRYLFFNTVSGSRFIYSPGSTEVSDDIIRKCGSLPLAIVSITSILE

KSRKMEQWGYVNKSLGYNLMKNPTLEGIKQVLDLRYNNLSEHLKPCVLYLSIYQEDYLIC

KDDLVNQWLAEGLICATKDHTKEEISEACFGELVSSKMIQPVHIDGNGDVMSFVIQHMVL

NFIRYKSIEENFVTVIHHSQTATKLSDKVRRLSLHFGNVKDAKLPINMRLSQVRTLAFFG

AYKYWWRSIKDQFPLLQVLILHFWHDEDIISFDLTIISQLFRLKYLKITSDVTLELQTKT

RGLQCLETLKIDARISTAPLDTTHLSGLLHLSLPADTNLANGIGHMTSLHTFGYFDLSYN

SVENVLSLGKLTNLRNLQLTCSTIRPNSLEIKLQCMGFILQKLSNLKSVTMSTAGSSCVN

STDASSANVSVRISGDGLSSMSSPPALVERLELLPRICIFSYLPKWISLLSKLRILKIGV

RELVRNDIDVLMGLRALTDLSLHVHTKPTEIIFFGGIGFKALNLYKLKLGYNVDGVDQES

TIPVGIQYLYGLKEISVKIGGADPEKYDRRAEELAFMIDSGLHDRCVSVTLQFVRQIFDF

NEDKSSLTQEEQRKLKQQEILEDDSDEEYDEIIQDSGEQEVKQ*

>KAS_RG045

MESAVASAILLKSVMGRLFTVLEKEYSKHRELAQETNSLQQDLRIVAAAMDDQLLSIRRS

DARTAVARLHSKEMLDLAHDIEDYVDRFIHHLTCRQQCASAGRNSLLDRVAHELKKVQSR

SSFANEIQKLKRRLRQVHQDVIKNNPLAGGQSSSPSPQDRRIADNPVGIEEPVEELLSLL

DEVEGEPERMRVISIVGFGGLGKTTLAKAVFDSPRVKEKFHLRAWVPAGASPETSSGMRG

ILRAVIQKILPNVAMDVDGQLETSLKEYLKDKRYLIVIDDIGMDQWSIISSTFKDNGTSS

RIILTTTIQSIANSCSHGNGYVHQMNTLGEEDCKEIALPTGIRSPELETGSVPLLGKCDG

LPLALVSVSDYLKSSCEPTGELCANLCRNLGAHLKEQDGHPSFSELRKVLLDNYDSLSGY

ALSCLLYLGIFPSNRPLKKKVVIRRWLAEGYARSDSLRNEEDIAVENFNKLIDRNIILPV

DTRNNSDVKTCKTHGIMHEFLLNMSLAQRFIMTLSRDHPRLISNARHLSVHDGELTGYVT

SDEEFSRVRSLTVFGDASDTVSYVRKCKLIRVLDLQECNDFADDHLKHICKLWHLKYLSF

GYNINVLPRSIEGLHCLETLDLRRTKIKFLPIEAVMLPHLAHLFGKFMLHKDDLKNVNKM

SKLNPCKKQKKGMNILPKFFTSKKSNLQTLAGFITGENEGFLQLMGHMKKLRKVKIWCKH

VAGSSNYIADLSQAIQEFTKVPIDSDSNRSLSLDSEECSENFLSALHLEPCSEDFKYHLR

SLKLQGRFLRLPPFVTSLSGLTELFISSATLTQDHLSALITLNRLLYLKLISDKLENFEM

KHGAFPSLRRLCFVVKSVTSDLPTIKQGALPNLVSLHLLCRGLVGLSGIEIRHLKHLKEV

VIDSDVTPQTKQDWAHAAKNHPNRPKFSWPRKVDLVESEEPAKHLKTEKRKYCSNYELDY

NLQEMRLSESRDHKRQKIGEGDTSKSSVGIVYPMYGDVETDRTQVHLFNQETRRYDRTEV

DQKCPEMLQEYKDKHSMVVDVDLRSDEQVNPPHPKLKNLMPGKEYDRQELIPTEGAKVGQ

CQSGGDEDQIVHNTNGKKVVVQANHFFEQEDQGSQVTMSYESSSLLSHMDTKSL*

>KAS_RG050

MAVYSVATGALAPVLSKLSALLGDEHLDLAERTRSDAMFIRSQLEAVHSLLLPRISWGMT

GEEVDALCKDELMAEVRELSYDMDDAIDEFFLEEPMAGGDGGPFDELKTRVEDVSKRFSD

SRRWRPPVEQHQPSLTAATVDCPPPHARFVHNMMDVSELVEMDKHEKELIKLLEQGADTS

IYASRWRIATPWHDKEQSIVVKVPEKRRDDMYDDALHWAVSSRLHGVPSGGAYGDYSRLQ

LRGEGANIRKLLSTLRNKVGRAQLVKVEDKRKRVEEATKPCEFHEVKTICILGLPGAGKT

TLAKLLYSHHSTTEQQFQHRAFVSLSPGANLTDTLTDILLQVGTYNDDATPYCGTGTPHQ

QYLIDNISAYLIGKKYLIIIDDVWHWEEWEVIRKSIPKNDLGSRIIMTTRLNSIAEKCRN

DDMDAFVYETEALDYVDAWLLCDKVARKSVTCMNINPCYDIVDMCYGMPLALIRVSSALA

EEIQALDSDERQIWRALRRVEDGILDIPSLKPLAESLCLGYDHLPLYLRTLLLCCSVYHW

LDGGIVQRGRLVTRWIAEGFVSEEKAAEGYFDELVGRGWMKHRELNEYEIHPMMLAILRY

KSKEYNFVTCLGTGSDTCTSASLSYSSPTMAIRRLCLQRGYPMKCFSSMDVSHTRSLVIL

GDVIGVPLDMFKRLRVLDLEDNIGIEDSHLKKICEQLESLRLLKYLGLKGTRITKLPQEI

QKLKHLEILYLRSTGIKELPREIGELKQLRTLDVRDTRISELPSQIGELKHLRTLDVSNN

MWNIIELPSQIGELKHLQTLDVRNTLVRELPWQAGQLSESLRVLIDEDDSEEGMQLPKGI

CEDLIKGIPKAELAKCNEVLSIAIADRLGPPSVGIFKVIGSHKCIPKVLKDHFDGLSRLD

IRLCKLDEKDHEFLANNMPNLQMLVLRLQAPQREPIIINCTGFQMLERFLLDSRVPWITF

QEGAMPKLKHLEFKFYAGPPSNDPAVGITHLLSLQNVVFRCSEWYKSDNPGIKATIDVVK

KDARQHPNRPISLLITEGDKEVPNIEAHGSSENIVIVHAAPDDAISCSSCGRTSTSIQEG

TVRDRIPAMDLFWPEFNSYEKAKRN*

>KAS_RG051

MELAVGASEATMRSLLGKLGNLLAQEYSLVSGVRGDIQYINDELASMQAFLRDLSVVTEG

HNHDNRRKDWMKQIRDVAYDVEDCIDDFAHRLPQDSISDAKCSFILTKMYELLTWWPRRD

IASRIAELKVRAQQIADRRNRYGVNNPEHCDSSNSPRPRAHAAAQDIAEYQDTKPQIVSI

KEPVGMKTVMENLEKWLTEPQPDKGRAVLSIVGFGGVGKTTIAMALYRKVSGKFDCQASV

AVSQNYDEDEVLRSILNQVSKQEEAGGSTESSSRDENTREPQGSSSTSSREENTAESGTK

RMLNKLKKALPLSLLGGNDDKTSVRQQETMGSLQLREELKRRLAEKRYILLIDDIWSAKT

WNSIIIPFLPSENDKDSRIIVTTRFHAVGSTCSPRHKNDEATSSPGHGKDLLHKVDFLTG

DKPLDLFNASIPDPMKRTDRDKKLSKICGGLPLAIVTMAGLVACNPNKANSDWSKLCESL

FPYPVTTLNLDGVTRILDCCYNDLPADLKTCLLYLSIFPKGWKISRKRLARRWIAEGFAT

EKQGLTEEEVAEAYFNQLARRNLIRPVEHGSNGKVKAFQVHDMVLEYIMSKSIEENFITV

VGGHWQMTAPSNKVRRLSLQSSGSKHGNSTKGLNLAQVRSLTVFGNLNHVPFHSFNYGII

QVLDLEGWKGLKERHVTEICQMLVLKYLSIRRTEIAKIPSKIEKLEYLETLDIRETYVEE

LPKSVGQLKRISSILGGNKNTRKGLRLPQEKRNKAMKNPSPQGKTKEPAEKGFLSQEKAK

GTMKSLRVLSGIEIVDESAAVAASLHQLTGLRKLAIYKLKISEENDTFKELLSSIEYLGS

CGLQTLAINDENSKFINSLYNMSAPPRYLVSLELSGKLKWLPKWITSITTLNKLTISITV

LTTETLEILRNLPSLFSLTFAFSLSAAKQDQDTVKGILEDNKLATDGEIVIPAKEFKSLK

LLRFFAPFVPKLSFPDKSAMPALEIIEMRFQEFEGLFGIEILENLREVHLKVSDGAEAIT

KFLVSDLKDNTEKPKVFVDGIVTA*

>KAS_RLK002

MGVGPHCTTSLLIILAVVITSSLLTTTIKADEPSNDTDIAALLAFKAQFSDPLGFLRDGW

REDNASCFCQWIGVSCSRRRQRVTALELPGIPLQGSITPHLGNLSFLYVLNLANTSLTGT

LPGVIGRLHRLELLDLGYNALSGNIPATIGNLTKLELLNLEFNQLSGPIPAELQGLRSLG

SMNLRRNYLSGLIPNSLFNNTPLLGYLSIGNNSLSGPIPHVIFSLHVLQVLVLEHNQLSG

SLPPAIFNMSRLEKLYATRNNLTGPIPYPAENQTLMNIPMIRVMCLSFNGFIGRIPPGLA

ACRKLQMLELGGNLLTDHVPEWLAGLSLLSTLVIGQNELVGSIPVVLSNLTKLTVLDLSS

CKLSGIIPLELGKMTQLNILHLSFNRLTGPFPTSLGNLTKLSFLGLESNLLTGQVPETLG

NLRSLHDLGIGKNHLQGKLHFFAVLSNCRELQFLDIGMNSFSGSIPASLLANLSNNLESF

YANNNNLTGSIPATISNLTNLNVISLFDNQISGTIPDSIVLMENLQALDLSINSLFGPIP

GQIGTLKGMVALYLGANKISSSIPNGVGNLSTLQYLFMSYNRLSSVIPASLVNLSNLLQL

DISNNNLTGSLPSDLSPLKAIGLMDTSANNLVGSLPTSLGQLQLLSYLNLSQNTFNDLIP

DSFKGLINLETLDLSHNSLSGGIPKYFANLTYLTSLNLSFNNLQGQIPSGGVFSNITLQS

LIGNAGLCGAPRLGFPACLEKSHSTSTKHLLKIVLPAVIAAFGAIVVFLYIMIGKKMKNP

DITTSFDIADAICHRLVSYQEIVRAIENFNEDNLLGVGSFGKVFKGRLDDGLCVAIKVLM

QVEQAIRTFDAECHVLRMARHRNLIKILNTCSNLDFRALLLQFMANGSLESYLHTENMPC

IGSFLKRMEIMLDVSMAMEYLHHEHYEVVLHCDLKPSNVLFDEEMTAHVADFGIAKMLLG

DDNSAVSASMPGTVGYMAPEYALMGKASRKSDVFSFGIMLLEVFTGKRPTDPMFIGGLTL

RLWVSQSFPENLIDVADEHLLQDEETRLCFDHQNTSLGSSSTSRNNSFLTSIFELGLLCS

SESPEQMMSMKDVVVKLKDIKKDYFASMLAMERPRRY*

>KAS_RLK003

MVLDGFVLGGGGNGTGGDDLSALLDFKAQLSDPLGVLATSWTTNASLCRWVGVSCSRRRR

RVVELHLRGVPLQGELTPHLGNLSFLRVLDLAAANLTGPIPANLGRLRRLKILDLAHNTL

SDAIPSALGNLTKLETLNLYDNHFSGHVPMELQNLYSLRVMALDQNYLTGPIPKHLFDAK

HSLTHIYLGDNSLSGPIPDSVASLSMLRVLSLPSNQLSGPVPPAIFNMSRLETISIRKNN

LTGAIPTNESFNLPMLRKIDLYMNKFTGPIPSGLASCKHLEMISLGDNLFEDVVPAWLAT

LSQLNSLSLGSNELVGPIPGQFGNLSMLNMLDLSFSKLSGPIPVELGMLSQLTFMSLSNN

QLNGTFPAFIGNLSELSHLELAYNQLTGHVPLTIGNNIRPLKHFEIRGNHLHGDLRFLSS

LSNSQRLEVLIISENLFTGCIPNSVGNLSTGILEFRANNNRLIGGLPAILSNLTNLRWIN

FADNQLSKPILPASLVTLENLLGFDLSKNSIAGPIPKEVSMLTRLVCLFLSDNKLSGSIP

DGIGNLTMLEHIHLSNNKLSSIVPTSIFHLNNLILLLLFNNALTGALPSDLSHFQNIDHI

DVSDNMLDGQLPNSYAYHPMLTYLNLSHNSFRDSIPDSFSHLTNLATLDLSYNNLSGTIP

KYLANFTYLTTLNLSFNKLEGEIPTRGVFSNITLKSLRGNAGLCGSPRLGLLPCPDKSLY

STSAHHFLKFVLPAIIVAVAAVAICLCRMTRKKIERKPDIAGATHYRLVSYHEIVRATEN

FNDDNKLGAGSFGKVFKGRLRDGMVVAIKVLNMQVEQAMRSFDVECEVLRMVRHRNLIRI

LNICSNLDFKALLLQYMPNGSLETYLHKEGHPPLGFLKRLDIMLDVSMAMEHLHYHHSEV

VLHCDLKPSNVLFDEEMTAHLADFGIAKLLLGDDNSAVSASMQGTLGYMAPEYASMGKAS

RKSDIFSYGIMLLEVLTGKRPTYPMFVGDMSLRKWVSDAFPARLLDVLDDRLLQGEILIQ

QGVLQKNATSLPYSATWANEDLLVAVFELGLMCCSNSPAERMEINDVVVKLKRIRKDYLT

CTKAI*

>RUF_RG002

MATIVDTLVGSCINKLQAIITDKTILILGVKDELEELQRRTNVIRSSLQDAEARRMEDSV

VEKWLDQLRDVMYDVDDIIDLARFKGSVLLPDYPMSSSRKSTACSGLSLSSCFSNIRIRH

EIAVKIRSLNKKIDNISKDEVFLKLNRRHHNGSGSAWTPIESSSLVEPNLVGKEVIRACR

EVVDLVLAHKAKNVYKLAIVGTGGVGKTTLAQKIFNDKKLEGRFDHRAWVCVSKEYSMVS

ILTQVLSNMQIHYEQNESVGNLQSKLKAGIADKSFFLVLDDVWHYKAWEDLLRTPLNAAA

TGIILVTTRDETIARVIGVDRTHRVDLMSADVGWELLWRSMNVKEEKQVKNLRDTGIEIV

RKCGGLPLAIRAIAKVLASLQDQTENEWRQILGKNAWSMSKLPDELNGALYLSYEVLLHQ

LKQCFLYCALFPEDATIFRDDLTRMWVAEGFIDEEKGQLLEDTAERYYYELIYRNLLQPV

GLYFDHSRCKIHDLLRQLACRLSREECFVGDPESLGSNSMCKIRRILVVTEKDIVVLPSM

DKERYKVRTYRISYEKPLQVDSSHFKKLKYLRVLDLTNSHVQRIPNYIENMIHLRLLDLD

WTDISHLPESIGSLQNLQILNLQRCKSLHSLPLATTQLCNLRRLGLAGTPINQVPKGIGR

LKFLNDLEGFPIGGESDNTKIQDGWNLEELAHLSQLRCLDMIKLERATPCSSTDSFLLAE

KKRLKLLALWCTEQTDESYSEENASNVENIFEKLTPPHNLEKLVIVNFFGCRFPTWLGTA

HLPSVKSVILTNCKSCVHLPPIGQLPNLKYLRIEGASAITKIGPEFVGCWEGNLRSTEAV

AFPKLEALVINDMPNWEEWSFVEEEEELQEEETTAAAKEGGEDGTAASKQKGEETLSPRS

LWLLPCLTKLELQDCPKLRALPPQLGQQATNLKDLLIREAECLKTVEDLPFLSGCLLVER

CEGLERISNLPQVRELYVNLCPNLRHVEELGSLEQLGLTKNMQEISKLWVPRLQEQHRRL

HGDEHELEVNEWL*

>RUF_RG003

MATILDSLVGSCANKLKEIITEEVILILGIQEELAELQRKTELIHCCISDAEARRMEESA

VDNWLGQLREVLYDVDDIIDLARFKGSILLTDHPSSSSRKSIACTGLSISTCFSNVQARH

EVAMKIRSLNRKIENISKDRVFLTLKSTVPTGSSSVLRVRKSSHLLEPNIVGKEIIHAYR

KMVDLVLEHKGRKLYKLAIVGTGGVGKTTLAQKIYNDRKIKGSFNKKAWVCVSKVYSEAS

LLRELLRIMEVHHDQDESIGELQSKLEIAIKETNFLLVLDDMWQSDAWENLLRIPLHAAE

TGTILITTRNNIVALEIGVDHTYRVDLMSTDVGWELLWKSMNISESIELQTLQDVGIEIV

RKCGCLPLAIKVIARVLASKEQTENEWKKILSKNAWFMNNLLNDLRGVLYLSYDELPRHL

KQCFLYCSVYPEDANIYRDDLTRMWIAEGFIEDHGGQLLEETADEYYYELIHQNLLQPDG

LYYDHSSCKMHDLLRQLACYLSREECFVGNPESLVGNTVSKLRRVSVVTDKNMVMLPRMD

EVQYKVRTWKTSYEKTLRVDNSFFKRFPYLRVLDLTNSFVPSIPGCIGNLIHLRLLDLDG

QVILDEPLLSPSLKSCSYEICPTGKSGSLLMKQRQLLRKGLMMEILSRLRRLELSGCPKL

KALPRQLAQINSLKEIELRWASSLKVVENFPLLSEMLLIATCQALEKVSNLPQVRELRLQ

DCPNLRLVEDLSTLEQLWLYEDMHEVSTLWVPGLQQQCRQHHGEDLDVYNWT*

>RUF_RG004

MATILGSLVGSCVNKLQGIITEEAILILGVKEELRKLQERMKQIQCFINDAERRGMEDSA

VHNWISRLKDVMYDADDIIDLASFEGNKLLNGHSSSPRKTTACSALSPLSCFSNIRVRHE

IGDKIRTLNRKLAEIEKDKIFATLENTQPADKGSTSELRKTSHIVEPNLVGKEIVHACRK

LVSLVVAHKEDKAYKLAIVGTGGIGKTTLAQKVFNDQKLKGTFNKHAWICVSQDYTPVSV

LKQLLRTMEVQHAQEESAGELQSKLELAIKDKSFFLVLDDLWHSDVWTNLLRTPLHAATS

GIILITTRQDIVAREIGVEEAHRVDLMSPAVGWELLWKSMNIQDEKEVQNLRNIGIEIVQ

KCGGLPLAIKVTARVLASKDKTENEWKRILAKNVWSMDKLPKEISGALYLSYDDLPLHLK

QCFLYCIVFPEDWTLKRDELIMMWVAEGFVEVHKDQLLEDTAEEYYYELISRNLLQPVDT

YFDQSRCQMHDLLRQLACYLSREECHIGDLKPLVDNTICKLRRMLVVGEKDTVVIPFTGK

EEIKLRTFTTDHQLQGVDNTFFMRLTYLRVLDLSDSLVQTIPDYIGNLIHLRMFDLDGTN

ISCLPESIGSLQNLLILNLKRCKYLHFLPLATTQLYNLRRLGLADTPINQVPKGIGRLKF

LNDLEGFPIGGGSDNTKMQDGWNLEELAYLPQLRHLGMIKLERGTPRSSTDPFLLTEKKH

LKVLNLHCTEQTDEAYLEENARNIEKIFEKLTPPHNLEKLVIVNFFGCRFPTWLGTNHLP

SVKYVVLIDCKSCVHLPPIGQLPNLKYLKINGASAITKIGPEFVGCWEGNLRSTEAVAFP

KLEELVIVDMPNWEEWSFVEEEEVQEEEAAAAAKEGGEDGTAASKQKGEEAPSSRSSWLL

PCLTRLQLVGCPMLRALPPQLGQQATNLKEFFIRYTSCLKTVEDLPFLSGYLLVDGCEGL

ERVSNLPQVRELLVNVCPNLRHVEELGGLEQLLLDEGMQEISSLWVPRLQEQHRQLHGDE

HELEVTEWL*

>RUF_RG005

MATILDSLIGSCAKKLQEIITEEAILILGVKEDLRELQEKMEQIRCFISDVERRGMEDSS

IHNWISRLKDAMYDADDIIDLASFEGSKLLNGHSCSPRKTIACSGLSLLSCFSNIRVHHE

IGNKIRSLNRKLEEIAKDKIFVTLENTQSSHKDSTSELRKSSQIAESNLVGKEILHASRK

LVSQVLTHKEKKTYKLAIIGTGGIGKTTLAQKVFNDGKLKQSFDKHAWICVSQDYSPASV

LGQLLRTIDAQCKQEESVGELQSKLESAIKDKSYFLVLDDVWQSDVWTNLLRTPLYAATS

GIVLITTRQDTVAREIGVEEPHHIDLMSPAVGWELLWKSINIEDEKEVQNLRDIGIEIVQ

KCGGLPLAIKVIARVLASKDKTENEWKKILVDKLPKEIRGALYLSYDDLPQHLKQCFLYC

IVYPEDCTIRRDDLIRLWVAECFVEVHKDQLLEDTAEEYYYELISRNLLQPVDTSFDPSK

CKMHDLLRQLACHLSREECYIGDPTSLVDNNMCKLRRILAITEKGMVVIPSMGKEEIKLR

TFRTQPNPLGIEKTFFMRFTYLRVLDLTDLLVEEIPDCVGYLIHLRLLDLGGTNISCLPN

SIGALKNLQMLNLQWCKSLYGLPSTITRLSNLRRLGLDFTPINQVPRGIGRLEFLNDLEG

FPVGGGSDNTKMQDGWNLQELSHLSQLRRLDLNKLERATPRSSTDALLLTDKKHLKSLHL

CCTEPTDEAYSEEAISNVDMIFEQLNCKSCVHLPPIGQLPNLKYLRIKGASAITKIGPEF

VGCWEGNLRSTEAVAFPKLKLLAIKDMPNWEEWSFVEEEEEKEVQEEEAAAAAKEGREDG

TAASKQKGEEAPSPTPRSLWLLPCLTKLQLVECPKLRALPPQLGQQATNLKELDIRRARC

LKTVEHLPFLSGILFVQSCQGLEIISNLPQVRELLVNHCPNLRHVEMLGGLEQLWLSKNM

QKISSLWVPGLEEQHRQLHGDEHKLEVNEWF*

>RUF_RG006

MAEAVILLTVKKIGVALGNEAINQATSYFKKSVTQLTELQGSMGRIRRELRLMHEFLSGM

DVRNRNNRKYEIWVEEVRMLVYQIEDIVDDYLHLVGHKHHIGWGTYLKKGFRRPNVLLSL

NKIASLVKDVEASLVHLFQAKERWVFMDVGAATGGESSSYIVVEKSRHLASISRSLDEED

LVGVDENKKKLHEWLSSNELQRDVIVVHGMGGLGKTALAADVYRSEKEKFECHAWVSISQ

TYSIKAVLKCLINELDEKKSIRGNISDMDTGGLQDELKKFLKDQKYLIVLDDVWVPEAVN

DLFGALVSNLSRSRVLVTTRIDGVAHLAFPDKRITLKPLSEQESWELFCRTAFPRDKDNE

CPAELMTLAKQIVSKCQGIPLAIVSVGRVLFVCEKTEEEFKRIHNQLDWELVNNPSLEHV

RNILYLSYIYLPTHLKSCFLYCSLFPEDYLFTRKRLVRWWIAEGFVEKRGISTMEEVAEG

YIKELVYRNMLQLVQKNSFGRMKSFRMHDILHELAVDLCRRECFGHSYNSKNKHEEFLEK

DERRMVIHKLDKDVNQAISSEWSRLRSFVTLERNMSSPNLLTLVEGKCRYMSVLELIGLP

KDNIPNVIGDLFNLKHLSLRDSMVKFLPNSIEKLSNLMTLDLCKSEIQELPGGIVKLKKL

RHLFAEKLNGKFWRDFQWSTVGRYFEDLCESLCQMEYLSLLNIAASDEEEVLQLNGLKWL

HPNVKKLRLIGRLAQTGLLSCAPEAGSHSLCSLCLFWSQLAEDPLPSLSRWSNLTDFRLT

RAYLGEQLVFLPGWFPRLKTLYLVDMPNLKRLKIHQGSITSLEELHLINLRGMTEVPSDI

IFLLPTLKYLYFLEITWDFFTALRRSRIGSIRWRYSLASDARL*

>RUF_RG007

MGGRSGGIQAIPTIISGNSYMDFSSRLVLPLVPSHFSEIPKSSRCLTWPIIVEFSPKPSL

VPLPLTNNWSKASTMADPVTIGAAVGWGMKAAGWIISPIISNLMKEGFSYLGFDTSKKLR

QLEMKVLELELMLGLEAAQIYPHRNRLEPLLKNLRSAYYEAEDILDDVEYHRLKGQIQSH

PCHRNWVHKIRSALPSCSFMKNQENGNMSDEAPSGISRKKLKKSLDKIENIINEAHRILP

LLSLPNQGNVNKRQIVHANSRSPVTTATPPPVVIGREKDCDNIISMLHEDVSNVQPGSSN

SFLCYSIIGIYGIAGSGKSTLAQLVCASEKKDKQEKKDGHFDLIMWVHVSQNFIVDTILT

EMLEAATGKKCDRFNNLDTLEQKLEEALGGKRFLLVLDDIWYHNSENQHEQQKILTPLRV

GNPGSKVLVTSRTEYALLALGALKCIPISDLDDNVFLKLFLHYALPLLSRSPLAARTVGG

QLQIRPNVDFWRDACNRDLLNETMGALWWSYQHLDEQVRRCFSYCSIFPRRHQLKRDELV

NLWVAEGFISTTDTGEEEAAAQQYFGELVSSSFLNKHMGEYSNEKNVYFTVYDLLHDLAE

KVAGSDCFRIQQDWTGVLPTNVRHLFIEAYDETIITERILEMESLHTLVIHCIGRDMMDH

ETVYESIFMKLRKLRVLKVQSDARWRLYTGFSVLSLPASIKKLKHLRYFGYQSGYVYKLV

LPSTISTLRHFQVLDFGNCMELVFSSEEDLSKLNSLRHVIVSPLFPLSIRHLGRLTSLQT

MPPIKVERGEGYELQQLRRLNKLRGRLEIQGPENVESKEAAAEANLGAKERLQQLVLVWE

DDNESCSPDVQEEVLEGLCPPMELESLEIKGYQGSRYPSWLVVQQNCGPNYLHELRLSGC

SRMGSIPALTHLCSLTIFNCSWDSLPDDMERLTSIKNLTLSHCYNILLLPALPKSLELLR

VDGCSTELTSSCRTTGHPNWHKIKHIPHKYIAVGTLSLPDNMEDLTSLKELVLEECDQIK

LLPALPLSLETFLVAFFGDKPVKRTKGSDHAVPARQQERNQGNIKQGGDPRKARSVGAET

VEYVKCHHARRVRREKAKYISVSSARFWQHKTYIRSRPPVRYLSTPSFPLPTLSLSNAGS

CGSSTDGSGRLHRLDEPCDGGCGRFLRLPSSARRHPRLTLPSSRAPRSPAVVSSAGAWRA

PPPLPQALPAAAPSAPSSPPSSSRVPVAASAPSAAPSSARHRPIGPKLPPVIVPSAGGYE

RPLHCPELCPPPSPTPPTPPTVVPTSSHCRPKLRGPLRRPELRPPSLR*

>RUF_RG009

MAKLVTETTKDGHHTTNVTTVERPTTALPHNRAALMVTSELLVKVTGRDKNREQLQTLLR

AEEHNVICIHGISGSGKTTLAQYVCEPEKKAGYFELVMWIHVTQNFTVDSIFREMFEAAS

GNKEPCPNSPDVLQTKLKGVLGEKRFLLVLDDVWYNKDDHKNKEQLQQVLSPLKVGKTGS

KILVTSRRALSHIGLVKCSPFPIPEMESDDFFQLFMHYALGDITLDDMDVRKHIGKQIVK

KLKGSPLAASIVGLRLRENQDISHWMEFIAQEHLDDVTDFLSWSYQHFDKQVRRCFSYCS

IFPRRHRLDRDELISMWVAQGFIRSTGTRKDLETLGQQYFDELVSFSFVNKHTEEHTSRV

YFTVHDLLHDLAEKVSGNDCFAVQKGWAGVLPRDVRHLFIEIYGDESISKKVLELEHLRT

LIIIHRRQKEKSSAEVFESVFAKLPKLRVLILKIETMDNPDVVNYVPESIDKLKKHLRYF

AFEGHVTEWKELPATFSQLYHLEVVHVKPFAFSSSWIYSTRTTNLRWSSYTTYHVEFPNI

GNLESLRTLGRFTVKTKQGLRKLTGYELYQLKRLNKLRGRLKIQFLNEDSSREDALEAEL

HKKVYLTELDLQWSFGWATPQEDVQSQVLEALRPPKQLQKLEIHSYKGSSYPSWMMRDDS

DVPMCLQLLWISDSTKLQAIPEHTVLLSNLRMLRISGDEWDYLPENMENLKLLVELEILD

CKKLRSLPTVWPPSLKKLTITMMSWHRLRSLPTMPESLEHFRLVSLRHKFTKSCETPGHQ

NYENIRHIPNKTISCGGCLNHNSSQGNEDEGLEDDEEFEDSEYDQEDDEDRYPLEGDQED

DEDFEASEDHGEDHEIVEASEDDQQDDEDVPAKKKDEDIEASEDDERK*

>RUF_RG010

MADPLTLAAVGWGISTAGWIISPVVTKLVNYCASSLGGFDASKKLRDLETFILPRLVVAL

EALEKSRRHELENLVRELKSALYEVEDILDEAEYCRLEKQVSRWKKDKKRKFAACYEAGP

SNQDVCKESSHTIPGPSRITLKELLKNIEKLIDEAEKIVGVARIMENDAHIPNVTSNYIQ

KPTTALHQNKKTTTSNPPLKVTGRDMHREQLKSMLRAIEHDDTCYSVIGIHGIAGSGKTT

LAQYVCKAEKNDTYFDLVMWIHVTQNFSVDTIYREMFEAASENKKPCPAYNSLDVLQIEL

MGVLHGKRFLLVLDDVWYNEYDHKDQELQQVLSPLEVGKRGSKILLTSRRALPGIGDVSC

TPYPLPEMDSEDFFQLFMYYALGGTTIDARDARKLIGKQIAKKLKGSPLLATIVGQRLRT

HKDVKYWIEFNAREHLDDVMDFLWWSYQHFDEEVRRCFAYCSIFPRRYKLKRDEIVKLWV

IQGFINAPKGEEKEVAAQRYFDDLLSALFLRPLHDHNPDHYPSKYFAMHDLLHDLAKKVA

GSECFTIKNGGTEVVPQDVRHLFVEISGEENISKVLELEKLRTLIITRTGLARKLTHVEE

FESVFAKLPKLRVLIMEVTCWFQNYEKVIFVPISIGRLKCLRYFSFSVWHMNNVELNLPH

TFSHLYHLKVLDVEFRKLTFSSREDTSNLINLQHVITYRKALDFPYIGRLESLQTLKHFT

IKKEKGYELYQLKLLNKLRGTLNIYGLENVSSKHDAINAELHEKVYLTKLELIWSRHTGT

SAEERYLQLEVLEALHPPMHLQGLVIHNYNGSCYPGWMMRGGSEVPMCLQDLMLVSCTKL

ASIPKHSVIFSHLHTLRIFACTWDSLPDNMENLELLVELQIDYCNYIRSLPEVLPPSLEK

LRVMAGCYRIRSLPILPQSLVYFELSSFHRELIRSCETPGDRNYENIRHIPNKKIKYLHV

SYSSCSEGKAFKPFVKLRLGCFTIFLSVVESIYLDCSSF*

>RUF_RG012

MEVAVSLSTGVLEVVLGKLGNILMDEYELQTGAKDGIRYIREELESMQTALEKVSKVPPD

QLDKQVKIWARKVREMSYSIEDTIDSFMLHVDDSDDGEYSGSSAACCPCVSAKSMLPRSY

KARRDIAAEINRIKEEVDEVSKRRERYKVDSIFVAPASYDPRLLALYEDKEKLVGIDHST

DEIIKLLSMEGEGASEQKLKLVSIVGPGGMGKTTLANAVYKKLEEQFDCTAFVSVSLQPN

VKNILSGLLRQVTASKVKDDLEEGKDKDKKRYLIVIDDIWEEQPWKLIKCALFENKLGSK

VITTTRNTEIAKLCSADEVDGIIHQLQPLSDGDSEQLLYYKIFKNEGCPTELKDVSQKIL

KKCKGWPLAINAIASLLANKPTQTQGQWYSVLNSISTGLENNHGVKDMRLILSLSYRDMP

AQLRDCLLYLSIFPEDHIIGRDDLIQRWIAEDLVHGRQDDYLYELGNKYFNELINRSMIQ

PIDVDAFGRAQACKVHDLVLEYINSLSAEEDFVTIFNGLQSFPQSDSIHRLSLRNSEGEH

GIPKAIKRLPHVRTLVVSSCFFYSTPSLSIFPVLRVLELQRCTESNIKGVENLVHLRYLR

LTQAYYFFYDGDADHCINLPERIGNLQLLQTLDLKDAMIKELPHTVVQFSQLRVLEISLR

KFDKRCETLLLQCLCNVKQLEALCINAPDLSLDFMLQVDWAPTHLRRFTASPREQSKHML

RSGWVELSPFSRLPRWINSSLLLSDLSIMVRTLAQEDLEILEVLPVLRSVDLEVIQATGT

RLEFNGSVGGNGHATAFQCLGNLKFASRAVGLVFKPGAVQELQKLYLCFDVAETKDVHGD

FDFHSLENIASLKTLDVDIDCRCARLWEVEAVEAALSNATKLNPNCPTLGLTRHFPDLIL

HDQEEEIPEHLQAKKKEDALLSRVGPFGGYGGRARDIRVTPHRLEDVTIHSANVVHSLAF

SYADHNGQQHSTGPWGSRDARVSTTPDYVFF*

>RUF_RG014

MEGTVFNLPGRLEELLCHHGSMLPKGADEEIPLIKQDLEEIISILHGHSEPKLEDHGMVV

RCWMKEVRELSYDIEDSIDQYEHAARSQNRPNIHHRKFNRWRGNKIPCIPQKLKQRLWMA

NKIREFSLRAQEALQRHAMYNNLGGVASTASTTRGDVCSATPLHPTQTHVGIDAAMDQLE

NLIDVCGEEKLKVVSIVGVGGVGKTTLANKLYHKLRWQFECWAFVRSSQKTDMRRLLINI

LSQVRPHQSPDNWKVHSLISSIRTYLQDKRFLIVIDDLWATSTWDIIKCALPEGNKSSRI

LTTTEIEDLALQSCSYDLKFIFKMIPFGEDDSRKLLFSIVFGSHSKCPPEVSETLYDIVR

KCGGLPLAIVTVASLLASQLEKQEQWDYINKSLGYSLMANPTLEGMKQLLNICYNNLPQH

LKVCMLYLSMYQENHIIWKDDLVSQWIAEGFICATEGHDKEEISRGYFDELVGRKIIQPV

HIDDSGEVLSCVVHHMVLNFITYKSIEENFIIAIDHSQATIRFADKVRRLSIHFSNVEDA

TPPTNMRLSQVRTVAFFGVLKYMPFVMEFRLVKVLVLHILGDEDSIGIFDLTKISELVRL

IYLKVTSNITVKLPTQMQGLQYLETLKIDGTISEVPTDIYLPGLLHLTLPAKTNLPNGIV

HMTSLRTIGYFDLSCNSAENLWSLGELTNLRDLQLAYSEIHSDNLKDNMKYLGSILGKLR

NLTSITLSPPGSSCPDTLHIDRDTKRRINVDGWSSVSSPPALLQRFELLPCVCVFSNLPN

WIGQLGNLCILKIGIREVTSNNIDVLGVLPELTVLSLYVHTKPAERIVFDNAGFSILKYF

EFICSVAWMKFEMGAMPSLRKLKLGFDVHRADQHDIIPVGIEHLSGLEEISAKIRVACSA

HDHCKRFAESALSNAFMMHPGRPSVNIRCVDWTFDGKDDDNVRTQEEEHRTLQKQHHTAK

EGSNEKSPVKQRDPREGAHKSVDGSPRTQCWYHRKTDYHSNRGLFIIE*

>RUF_RG015

MESAAASAFLKTVMGRLFMALEKEYNKHRGLAQESHSLQQDLRMIAAAMDDHQLSMGKSD

AAARTAVARLHTEEMLDLAHDIEDCVDRFLHRLTCNHHKRGGAGAGASLLRRVTHELSKV

KSRSSFGDEIQKLKKRLREAHQRVLTINPPPILTAGGGQHTASSSSAAVAPPCRAARSPV

GIGEDVEELLSMLDEVEGEPVQMRVVSVVGFGGLGKTTLAKAVYDDPRAKDKFRHRAWVA

AGGSPEIRGILRDVLQQVRPDDAMDVDGQRLEASLKDYLKDKRYLIVIDDIGMDQWSIIS

SAFEDNGTSSRIILTTTIQSVANMCSHGSGYVYQMNTLGEDDSKKLAFPGCRSPELEQGS

ASLLGKCDGLPLALVSVSDYLKSSSEPTGELCAKLCCNLGSHLKEKPGHDNFSELRKVLL

DNYDSLSGYALSCLLYLGIFPSNRPLKKKVVIRRWLAEGYARSDSLHSEEDIADENFSKL

IDRHIIQPIDTRNNSEVKTCKTHGIMHEFLLNKSLTQRFIATSSHDHPRVGIDTTNARHL

SVHAGELTECVASDEELSRVRSLTIFGDAGDAISYFRKCKLIRVLDLQEWNNLDDDHLKH

ICKLWHLKYLSFGGNISELPRSIEGLHCLETLDLRRTKIKFLPIETIMLPHLAHLFGKFM

LHKDDLKNVNKMSKLQKFFSSNKSNLRTLAGFITDQGKGFLQLVRQMKKLRKVKIWCKHV

AGSSNYIADLSQAIQEFTRTPIDRDSDRSLSLDCEECSENFLSSLDLEPCFEDFKYHLRS

LKLHGKLLRLPPFVASLSGLTELCISSAILTQDHLSALIKLNRLLYLKLIADKLENIDIK

IGAFPSLRRLCFVMKSVTSALPTIEQGALPNLVSLQLLCQGLVGLSGIEIRHLKHLKEIT

IDSTVTVQTRQDWEQAAKNHPNRPRVLLFGKVDPMESEEPEKPCAIGEKRKLSVAQPTGS

DGGLDSSLKKMRLSEPSSSRLQVIVHPVVVTATEAAPQHSFANL*

>RUF_RG017

MDNQLLSIRRSDARTAVARLHSKEMLDLAHDIEDYVDRFIHHLTCRQQCASAGRNSLLDR

VAHELKKVQSRSSFANEIQKLKRRLRQVHQDVIKNNPLAGGQSSSPSPQDRRIADNPVGI

EEPVEELLSLLDEVEGEPERMRVISIVGFGGLGKTTLAKAVYDSPRVKEKFHLRAWVPAG

ASPETSSGMRGILRAVIQKILPNVAMDVDGQLETSLKEYLKDKRYLIVIDDIGMDQWSII

SSTFKDNGTSSRIILTTTIQSIANSCSHGNGYVHQMNTLGEEDCKEIALPTGIRSPELET

GSVPLLGKCDGLPLALVSVSDYLKSSCEPTGELCANLCRNLGAHLKEQDGHPSFSELRKV

LLDNYDSLSGYALSCLLYLGIFPSNRPLKKKVVIRRWLAEGYARSDSLRNEEDIAVENFN

KLIDRNIILPVDTRNNSDVKTCKTHGIMHEFLLNMSLAQRFIMTLSRDHPRLISNARHLS

VHDGELTGYVTSDEEFSRVRSLTVFGDTSDTVSYVRKCKLIRVLDLQECNDFADDHLKHI

CKLWHLKYLSFGYNINVLPRSIEGLHCLETLDLRRTKIKFLPIEAVMLPHLAHLFGKFML

HKDDLKNVNKMSKLNPCKKQKKGMNILPKFFTSKKSNLQTLAGFITGENEGFLQLMGHMK

KLRKVKIWCKHVAGSSNYIADLSQAIQEFTKVPIDSDSNRSLSLDSEECSENFLSALHLE

PCSEDFKYHLRSLKLQGRFLRLPPFVTSLSGLTELFISSATLTQDHLSALITLNRLLYLK

LISDKLENFEMKHGAFPSLRRLCSVVKSVTSDLPTIKQGALPNLVSLHLLCRGLVGLSGI

EIRHLKHLKEVVIDSDVTPQTKQDWAHAAKNHPNRPKFSWPRKVDLVESEEPAKHLKTEK

RKYCSNYELDYNLQEMRLSESRDHKRQKIGEGDTSKSSVGLVYPMYGDVETDRTQVHLFN

QETRRYDRTEVDQKCPEMLQEYKDKHSMVVDVDLRSDEQVNPPHPKLKNLMPGKEYDRQE

LIPTEGAKVGQCQSGGDEDQIVHNTNGKKVVVQANHFFEQEDQGSQVTMSYESSSLLSHM

DTKSL*

>RUF_RG018

MEAPTCALWGAMFNLPRRIEVLLRRHGNILPKGAEEEIPLIKRDLEEIISILHNHDDEEM

EGHASGAIMVRRCWLKEVRELGYDIEDCIDQYEHGAAGCSSSRSILHPRRKITRRRRRTI

KMPPRLPDKLKQRLWMANMIREFSLRVQEALQRHGTYNLGGSSSSTNDDASSDHRLSVGE

YAHDCRHFGIHSTAMDKLREWLDVDGGEEKLKAVFLVGAGGVGKTTVASELYGELRRRFE

CGAFVRTSQNPNITRLLISVLSQVRPQQSPENWKVHTLISSIRTHLQDKRYLIIIDDLWA

TSTWDIIKCALPDGNNCSRILTTTEIEDLAFQSYDYDYKYVFKMKPLGEDDSRDLFFSTV

FGPNSTCPTNLREVSCDIIRKCGGLPLAIVTIASLLAKLRKWEQWGYVNKNLGYSLMTNP

TMEGIEQVLNLSYNNLPQHLKPCMLYLSIYQEDYIIWKDDLVNQWMVEGLICGIQGHDNE

EISGTYFEELVGRKMIQPVHINENGKVLSCVIHPMVLNFIKCKSIEENFTTAIDHSQINT

VIADKVRRLSIHFGNTKDVSIPTNMRLSQVRTLAIFGFFKCMPFIVDFRLLKVLILHFWD

DEDSTSFDLTKISELFRLRYLKIISNVTLKLQKQIQGLQHLETLKIDARVSAVPSDITHL

TGLLHLNLPADTVLPDGIGQMTSLRTLSFYLNGNSIENVISLGELTNIRDLQFTCSSIQP

DNLKKKMQCLRSIIQKLKNLKSITLLPTRSSYANSLEDAGATNMRIHVDDLSCVSSPPAH

LERLELLPRICILSYFPMWIGNLSKLCILKIGVRELVKNDIDVLGGLPALIVLSLYVHTK

PEEIIVFDKTGFPVLKYLKFNCCVPWLRFKEDSVHNLRKLKLGYNAHRADEESTIPDGME

YLSLHLNEVSVKIGVADPEKYDKLSAELEYKLAFGFDMIHPTVTIRCVKHIFDCKVSKSR

LAQEDYGKVEQHEILEEDTDVPDEVDEIKQDYGQEASKRADSK*

>RUF_RG020

MELAVGASEATMRSLLGKLGNLLAQEYSLVSGVRGDIQYINDELASMQAFLRDLSVVTEG

HNHDNRRKDWMKQIRDVAYDVEDCIDDFAHRLPQDSISDAKCSFILTKMYELLTWWPRRD

IASRIAELKVRAQQIADRRNRYGVNNPEHCDSSNSPRPRAHAAAQDIAEYQDTKPQIVSI

KEPVGMKTVMENLEKWLTEPQPDKGRAVLSIVGFGGVGKTTIAMALYRKVSGKFDCQASV

AVSQNYDEDEVLRSILNQVSKQEEAGGSTESSSRDENTREPQGSSSTSSREENTAESGTK

RMLNKLKKALPLSLLGGNDDKTSVRQQETMGSLQLREELKRRLAEKRYILLIDDIWSAKT

WNSIIIPFLPSENDKDSRIIVTTRFHAVGSTCSPRHKNDEATSSPGHGKDLLHKVDFLTG

DKPLDLFNASIPDPMKRTDRDKKLSKICGGLPLAIVTMAGLVACNPNKANSDWSKLCESL

FPYPVTTLNLDGVTRILDCCYNDLPADLKTCLLYLSIFPKGWKISRKRLARRWIAEGFAT

EKQGLTEEEVAEAYFNQLARRNLIRPVEHGSNGKVKAFQVHDMVLEYIMSKSIEENFITV

VGGHWQMTAPSNKVRRLSLQSSGSKHGNSTKGLNLAQVRSLTVFGNLNHVPFHSFNYGII

QVLDLEGWKGLKERHVTEICQMLVLKYLSIRRTEIAKIPSKIEKLEYLETLDIRETYVEE

LPKSVGQLKRISSILGGNKNTRKGLRLPQEKRNKAMKNPSPQGKTKEPAEKGFLSQEKAK

GTMKSLRVLSGIEIVDESAAVAASLHQLTGLRKLAIYKLKISEENDTFKELLSSIEYLGS

CGLQTLAINDENSKFINSLYNMSAPPRYLVSLELSGKLKWLPEWITSITTLNKLTISITV

LTTETLEILRNLPSLFSLTFAFSLSAAKQDQDTVKGILEDNKLATDGEIVIPAKEFKSLK

LLRFFAPFVPKLSFPDKSAMPALEIIEMRFQEFEGLFGIEILENLREVHLKVSDGAEAIT

KFLVSDLKDNTEKPKVFVDGIVTA*

>RUP_RLK002

MALGLLVWIYIVLLIALSTVSAASPPGPSKSNGSETNLAALLAFKAQLSDPLGILGGNWT

VGTPFCRWVGVSCSHHRQRVTALDLRDTPLLGELSPQLGNLSFLSILNLTNTGFTGSVPN

DIGRLHRLEILELGYNTLSGSIPATIGNLTRLQVLDLQFNSLSGPIPADLQNLQNLSSIN

LRRNYLIGLIPNNLFNNTHLLTYLNIGNNSLSGPIPGCIGSLPILQTLVLQVNNLTGPVP

PAIFNMSTLRALALGLNGLTGPLPGNASFNLPALQWFSITRNDFTGPIPVGLAACQYLQV

LGLPDNLFQGAFPPWLGKLTNLNIISLGGNQLDAGPIPAALGNLTMLSVLDLASCNLTGP

IPADIRHLGQLSELHLSMNQLTGSIPASIGNLSALSYLLLMGNMLDGLVPATVGNINSLR

GLNIAENHLQGDLEFLSTVSNCRKLSFLRVDSNYFTGNLPDYVGNLSSTLQSFVVAGNKL

GGEIPSTISNLTGLMVLALSDNQFHSTIPESIMEMVNLRWLDLSGNSLAGSVPSNAGMLK

NAEKLFLQSNKLSGSIPKDMGNLTKLEHLVLSNNQLSSTVPPSIFHLSSLIQLDLSHNFF

SDVLPVDIGNMKQINNIDLSTNRFTGSIPNSIGQLQMISYLNLSVNSFDDSIPDSFGELT

SLQTLDLSHNNISGTIPKYLANFTILISLNLSFNNLHGQIPKGGVFSNITLQSLVGNSGL

CGVARLGLPSCQTTSPKRNGRMLKYLLPAITIVVGAFAFSLYVVIRMKVKKHQKISSSMV

DMISNRLLSYHELVRATDNFSYDNMLGAGSFGKVYKGQLSSGLVVAIKVIHQHLEHAMRS

FDTECHVLRMARHRNLIKILNTCSNLDFRALVLEYMPNGSLEALLHSEGRMQLGFLERVD

IMLDVSMAMEYLHHEHHEVVLHCDRLKSNVLLDDDMTAHVSDFGIARLLLGDDSSMISAS

MPGTVGYMAPEYGALGKASRKSDVFSYGIMLLEVFTGKRPTDAMFVGELNIRQWVYQAFP

VELVHVLDTRLLQDCSSPSSLHGFLVPVFELGLLCSADSPEQRMVMSDVVVTLKKIRKDY

VKSISTTGSVALPAYTKE*

>RUP_RLK004

MAWLLLPPFNSIRLLMLVLPLTIPYASGSIPRDGGSSSNGTGDDLSALLAFKARLSDPLG

VLAGNWTTKVSMCRWVGVSCSRRRPRVVGLKLWDVRLQGELTPHLGNLSFLRVLNLGGIN

LTGPIPADLGRLHRLRILRLAHNTMSDTIPSALGNLTKLEILNLYGNHISGHIPAELQNL

HSLRQMVLTSNYLSGPVPEYLFSATPWVTEIHLGLNSLSGSIPDCVGSLPMLRVLALPDN

QLSGPVPPAIFNMSSLEAILIWKNNLTGPIPTNRSFNLPMLQDIELDTNNFTGLIPSGLA

SCQKLEIISLSENLFSGVVPQWLAKMSRLTLLFLDGNELVGTIPSLLGNLPMLSELDLSD

SNLSGHIPVELGTLTKLTYLDLSFNQLNGAFPAFVGNFSELTFLGLGYNQLTGPVPSTFG

NIRPLVEIKIGGNHLQGDLSFLSSLCNCRQLQYLLISHNSFTGSLPNYVGNLSTELLGFE

GDDNHLTGGLPATLSNLTNLRALNLSYNQLSDSIPASLMKLENLQGLDLTSNGISGPIPE

EIGTARFVWLYLTDNKLSGSIPDSIGNLTMLQYISLSDNKLSSTIPTSLFYLGIVQLFLS

NNNLNGTLPSDLSHIQDMFALDTSDNLLVGQLPNSFGYHQMLAYLNLSHNSFTDSIPNSI

SHLTSLEVLDLSYNNLSGTIPKYLANFTYLTTLNLSSNKLKGEIPNGGVFSNITLISLMG

NAALCGLPRLGFLPCLDKSHSTNGSHYLKFILPAITIAVGALALCLYQMTRKKIKRKLDI

TTPTSYRLVSYQEIVRATESFNEDNMLGAGSFGKVYKGHLDDGMVVAIKVLNMQEEQAMR

SFDVECQVLRMVRHRNLIRILNICSNLDFKALLLQYMPNGSLETYLHKEGHPPLGFLKRL

DIMLDVSMAMEHLHYHHSEVVLHCDRLKSNVLFDEEMTAHVADFGIAKLLLGDDNSAVSA

SMPGTIGYMAPEYVFMGKASRKSDVFSYGIMLLEVFTGKRPTDAMFVGDMSLRKWVSEAF

PARPADIVDGRLLQAETLIEQGVHQNNATSLPRSATWPNEGLLLPVFELGLMCCSSSPAE

RMEINDVVVKLKSIRKDYFAFTGAI*

>RUP_RLK010

MAFRMPVRISVVLLIIALSAVTCASAVPSKSNGSDTDYAALLAFKAQLADPLGILASNWT

VNTPFCRWVGIRCGRRHQRVTGLVLPGIPLQGELSSHLGNLSFLSVLNLTNASLTGSVPE

DIGRLHRLEILELGYSSLSGGIPATIGNLTRLRVLYLEFNQLSGSIPAELQGLGSIGLMS

LRRNYLTGSIPNNLFNNTPLLAYLNIGNNSLSGSIPASIGSLSMLEHLNMQVNLLAGPVP

PGIFNMSTLRVIALGLNTFLTGPIAGNTSFNLPALQWLSIDGNNFTGQIPLGLASCQYLQ

VLSLSENYFEGVVTASAAWLSKLTNLTILVLGMNHFDAGPIPASLSNLTMLSVLDLSWSN

LTGAIPPEYGQLGKLEKLHLSQNQLTGTIPASLGNMSELAMLVLEGNLLNGSLPTTVGSI

RSLSVLDIGANRLQGGLEFLSALSNCRELYFLSIYSNYLTGNLPNYVGNLSSTLRLFSLH

GNKLAGELPTTISNLTGLLVLDLSNNQLHGTIPESIMEMENLLQLDLSGNSLAGSVPSNA

GMLKSVEKIFLQSNKFSGSLPEDMGNLSKLEYLVLSDNQLSSNVPPSLSRLNSLMKLDLS

QNFLSGVLPVGIGDLKQINILDLSTNHFTGSLSDSIGQLQMITYLNLSVNLFNGSLPDSF

ANLTGLQTLDLSHNNISGTIPKYLANFTILISLNLSFNNLHGQIPKGGVFSNITLQSLVG

NSGLCGVAHLGLPPCQTTSPKRNGHKLKYLLPAITIVVGAFAFSLYVVIRMKVKKHQMIS

SGMVDMISNRLLSYHELVRATDNFSYDNMLGAGSFGKVYKGQLSSGLVVAIKVIHQHLEH

AMRSFDAECHVLRMARHRNLIKILNTCTNLDFRALILEYMPNGSLEALLHSEGRMQLGFL

ERVDIMLDVSMAMEYLHHEHHEVVLHCDRLKSNVLLDDDMTAHVSDFGIARLLLGDDSSM

ISASMPGTVGYMAPEYGALGKASRKSDVFSYGIMLLEVFTGKRPTDAMFVGELNIRQWVY

QAFPVELVHVLDTRLLQDCSSPSSLHGFLVPVFELGLLCSADSPEQRMAMSDVVVTLKKI

RKDYVKSISTTGSVALPAYTKE*

>NIV_RG001

MATIVDTLVGSCINKLQAIITDKAILILGVKDELEELQRRTDLIRSSLQDAEARRMKDSA

VQKWLDQLRDVMYDVDDIIDLARFKGSVLLPNYPMSSSRKSTACSGLSLSSCFSNIRIRH

EVAVKIRSLNKKIDNISKDDVFLKLSRTQHNGSGSAWTPIESSSLVEPNLVGKEVVHACR

EVVDLVLAHKAKNVYKLAIVGTGGVGKTTLAQKIFNDKKLEGRFDHRAWVCVSKEYSMVS

LLTQVLSNMKINYEQNESVGNLQSKLKAGIADKSFFLVLDDVWHYKAWEDLLRTPLNAAA

TGIILVTTRDETIARVIGVDRTHRVDLMSADVGWELLWRSMNIKEEKQVKNLRDTGIEIV

RKCGGLPLAIRAIAKVLASLRDQTENEWRQILGKNAWSMSKLPDELNGALYLSYEVLPHQ

LKQCFLYCALFPEDANIFCGDLTRMWVAEGFIDEQEGQLLEDTAERYYHELIHRNLLQPD

GLYFDHSWCKMHDLLRQLASYLSREECFVGDPESLGTNTMCKVRRISVVTEKDIVVLPSM

DKDQYKVRCFTNLSGKSARIDNSLFERLVCLRILDLSDSLVHDIPGAIGNLIYLRLLDLD

RTNICSLPEAIGSLQSLQILNLQGCESLRRLPLATTQLCNLRRLGLAGTPINQVPKGIGR

LKFLNDLEGFPIGGGNDNTKIQDGWNLEELAHLSQLRCLDMIKLERATPCSTTDPFLLSE

KKHLKVLELQCTEPTDEAYSEEGISNVEMIFEELSPPRNLEDLMIVLFFGRRFPTWLSTS

LLSSLTYLKLKDCKSCVHLPPIGQLPNLKYLRINGASAITKIGPEFVGCWEGNLRSTEAV

AFPKLEWLVIEEMPNWEEWSFVEEEEVQEEEAAAAAKEGGEDGTAASKQKGEEAPSPTPR

SSWLLPCLTRLELVGCPKLRALPPQLGQQATNLKELDIRRARCLKTVEDLPFLSGMQEIS

QLWVPGLQEQHRQLHGDEHELEVIEWL*

>NIV_RG002

MSFSMATILDSLIGSCAKKLQEIITKEAILILGVKEDLRELQEKMEQIRCFISDVERRGM

EDSSIRNWISRLKGAMYDADDIIDLASFEGSKLLNGHSCSPRKTIACSGLSLLSCFSNIR

VHHEIGNKIRSLNRKLEEIAKDKIFVTLENTQSSHKDSTSELRKSSQIAESNLVGKEILH

ASRKLVSQVLTHKEKKTYKLAIIGTGGIGKTTLAQKVFNDEKLKQSFDKHAWICVSQDYS

PASVLGQLLRTIDAQCKQEESVGELQSKLESAIKDKSYFLVLDDVWQSDVWTNLLRTPLY

AATSGIVLITTRQDTVAREIGVDEPHHIDLMSPAVGWELLWKSINIEDEKEVQNLRDIGI

EIVQKCGGLPLAIKVIARVLASKDKTENEWKKILANYVWSMDKLPKEIRDCTIRRDDLIR

LWVAEGFVEVHKDQLLEDTAEEYYYELISRNLLQPVDTSFDQSKCKMHDLLRQLACHPSR

EECYIGDPTSLVDNNMCKLRRILAITEKGMVVIPSMGKEEIKLRTFRTQPNPLGIEKTFF

MRFTYLRVLDLTDLLVEEIPDCVGYLIHLRLLDLGGTNISCLPNLLVPRGIGRLEFLNDL

EGFPVGGGSDNTKMQDGWNLQELSHVSQLRRLDLNKLERATPRSSTDALLLTDKKHLKSL

HLCCTEPTDEAYSEEAISNVEMIFEQLSTPRNLEDLMIVLFFGRRFPTWLSTSLLSSLAY

LKLKDCKSCVHLPPIGQLPNLKYLRIKGASAITKIGPEFVGCWEGNLRSTEAVAFPKLKL

LAIEDMPNWEEWSFVEEEEEKEVQEEEAAAAAKEGREDGTAASKQKGEEAPSPTPRSLWL

LPCLTKLQLVECPKLRALPPQLGQQATNLKELDIRRARCLKTVEHLPFLSGILFVQSCQG

LEIISNLPQVRELLVNHCPNLRHVEMLGGLEQLWLSKNMQKISSLWVPGLEEQHRQLHGD

EHKLEVNEWF*

>NIV_RG003

MPYGNLEASENGNISDETPSDISRQTLKISLEKIENIINEAHGILPLLNLSNQSNVIERH

IGHANYSRSSVTTATPPPVVIGQEKDCHDIIDMLHQDAISVQPGSSNSVLCYSIIGIHGI

AGSGKSTLAQLVCANEKKDKQEKKDGHFDLIMWVHVSQHFSVDTILTEMLEAATGKKCGR

FNNLDILEQKLEEALGGKRFLLVLDDIWCHNSENQHKQQKILTPLRVGKPGSKVLVTSRT

ENALLALGALKCIPISELDDNAFLKLFLHYALPSVNMDEQDQKQLEEIGANIAKKLRRSP

LAARTVGGQLQIRPNVDFWRDACNRDLLNETMGALWWSYQHLNEQVRRCFSYCSIFPRRH

QLKRDELVTLWVAEGFITTADVGEEEVVARQYFDDLVSSSFLQTRTRGYGLKDYFTVHDL

LHDLAEKVAGNNCYRIQRGWAGGLLPQDVRHLYIETYDNTMITERILELENLRTLIIARD

RTNMIVNETVFESIFTKMKKLRVLIVETFSFREQNMLSFPASFSELKHLRYFGFPVGWRC

KLVFPSTFTKLYHLQVFDIGACGDLVFASKEDLCELTNLRHVIGSIMTVPNFGRLTSLLT

VPNIRVTKEVGYELQQLAQLNKLRGKLWIRGLQNVESKEAAVQANLAAKEHLQELTLTWD

GKIGSCPDVEAEVLEGLCPPTDLEILKIMDYKGSKYPSWLVGQQNSGPKHLRTLELSGSS

RLISIPEHNELFRNLYSLVISYCSWDSLPENMERLTSLKKLVFENCDGSWLLPALPQSLE

EFDVTYCYELAWSCQKIDHQNWQKIKNIPKKKL*

>NIV_RG004

MGDVPIGSNYTVPGTSKHRLKELLEHIVNLIDGADNIVALANLPEITENRAHIPGRALPP

RSTSNPPKVTGRDKTREELKNKLRDTEHDGSGTCYSVIGIHGIPGAGKTTLAQYVCEFEK

RDDYFDLVMWIHVTENFSVEGIYREMFEAASGKKCPRYNSLDALQINLKQVLHEKRFLLV

LDDIWCNKYNQGEKLEDLVRPLEAGKEGSKILATSRSTDAFSQLGPVRVDRFVIPELGRG

DFRQLFMYYAVGDITTIDDRDRRLIQVIGAEIAKKLKGSPLAATIVGAQLRHKDLDYWRD

YNNAREHFDDVMNSLALSFLQQLDEEVRICFAYCSMFPRKYNFERDELVKLWVTQGFINT

KSGEEKEAAAQRYFESLLSALFLQPSYDDNNERCYTMHDLLYDLAKEVAGSEYFTIENGK

IGGFSQDVRHLFIGIRYGKENIIKNLLELKELRTLIIDHTGLNDNEFISEIFDRVYTKLP

KLRNEEGYELYQLKRLNKLRCKLNIHGLRNVSNKEDALQAELHKKKNLTRVKLSWYSDSS

GGVISAQEEDLQSEVLEALRPPVWLQKLRIVCYKGSAYPDWMMTNGPEVPKYLQDLKLEN

CRPLGRKIPEHNELFKYLRKLHVFDCSWTYFPANMENLKSLQELLIHFCRNLLSLPTKLP

LSLLKLEIAECRSIKKLPVMPHSLLELILSSHNQEFITSCKTPGHQYFENIRHIPRKEIE

YEEDNLSTDSDEDEEREDILYPSKDDHASVVQGNCSIPNETGQQQQPPR*

>NIV_RG005

MQAAASASTGLLDAVLRKLGKMLADEYELQTGAKDGIRYIRAELESMQAALAKVSEVPPE

QLDNQVKLWAKKVRETSYSIEDTIDSFMLHVGHGDADDESGICACVRTISMLPWRYKSQR

DLATEIKRIKEEVDEVGNRRERYKLDSIIVATAPSDPRLLALYEDKAKLVGIDHSTDEII

KLLSMEREGDGTSEQKLKLVSIVGPGGMGKTTLANAVYQKLEEKFDCTAFVSVSLQPDVK

NILSGLLRQVTASKVKDDLEEDEGKDKDKKRYLIVIDDIWEEQPWKLIKCALFENKLGSK

VITTTRNTEIAKLCSADEVDGIIHQLQPLSDGDSEQLLYYKIFKNEGCPTELKDVSQKIL

KKCKGWPLAINAIASLLANKPTQTQGQWYSVLNSISTGLENNHGVKDMRLILSLSYRDMP

AQLRDCLLYLSIFPEDHIIGRDDLIQRWIAEDLVHGRQDDYLYELGNKYFNELINRSMIQ

PIDVDAFGRAQACKVHDLVLEYINSLSAEEDFVTIFNGLQSFPQSDSIHRLSLRNSEGEH

GIPKAIKRLPHVRTLVVSSCFFYSTPSLSIFPVLRVLELQRCTESNIKGVENLVHLRYLR

LTQAYYFFYDGDADHCINLPERIGNLQLLQTLDLKDAMIKELPHTVVQFSQLRVLEISLR

KFDKRCETLLLQCLCNVKQLEALCINAPDLSLDFMLQVDWAPTHLRRFTASPREQSKHML

RSGWVELSPFSRLPRWINSSLLLSDLSIMVRTLAQEDLEILEVLPVLRSVDLEVIQATGT

RLEFNGSVGGNGHATAFQCLGNLKFASRAVGLVFKPGAVQELQKLYLCFDVAETKDVHGD

FDFHSLENIASLKTLDVDIDCRCARLWEVEAVEAALSNATKLNPNCPTLGLTRHFPDLIL

HDQEEEIPEHLQAKKKEDVRTILIFCNLCSFIFVLIFLFSFLIS*

>NIV_RG006

MDRLYLVLDPKYKNRRTANGDKKTKSIKNRVEFAAAAASSYGHIPRVAAGSGLPSCAAGA

RYRRHFNRGGRQQGTEGAAVDATRGCWGRREPLLPPPAAVARPPPPPDLPAAGGMRVEEG

EGIAVGKRRWRWVPFPSPRRARRREEEGEMGEEGAREAGRRERAPCRCRWGGETGRQRER

RERSGATISCIVSPAGHHAAPLPLTTVRRSPPSPPPSSPPRVAGCPPPWPKKASSRRSSW

QDETCVAVSGLVGTVTAAGMETDEPATKAAGGEPKPSLATKAAGEQEKDDEEESSGGGGA

EPEVSATPAVVVFSSSHMFTGEEKETREGYFGRLKCKKGENLMKWRIWQSNGLASVESGI

LTMPFRRMALWRNPLLGVAECLISPREEIRFERRWGDIFLCCSGYTTSYQTASCKLGEAM

RSKSLHSMANPWRIAAMGSVITVLGWLLSPIISLLVNRFISYLFDASPKIQELEIQTVPK

LEQMLRKIEEERMHRKAKKERSAVQNLDTLAKLVKSALYEAEDVLDLIAYHQIEKDVIGD

DEPQGSSSKWHPHIDDAIHACKTSWIGRCITTLLEWAQGLYRSLRSRSAALLPISRSRCC

GSASDSLLERLSCFSGQFDFIRCCQSLFIWSVNWFEVARSYRDWFYDATGITATGYQLED

GTAVYSFMPAIARWKLRKRIEKLENTVTNVEKSHYLTQTSSGAWNDIVNMNRRSITSSST

RKVFGRDRERDMIRSMLREDDSLPSSSSRKCYSVICIYGIPGSGKTTLAQYVCEYEKEDK

YRYFDTVMLIYVSKTYRLEDIFRDMLEEITRNRHSEINDCKGLEAKLVENLRGKRFLLVL

DDLWVNDENHEKLLSPLSVGKSGSRILVTAQSKEAALGSNRLIPISDLEEEQYFSMFMHY

ALDSTIFDDREYIPIGRKIAKKLNRSPIAAVTVAGQLWRNPDIRFWQTTANLDVLNKTKG

ALWWSYNQLVVDVRRCFQYCSIFPRRYELERDNLVRMWIAQGFVKDNDGNNEDVEDVGQD

YFHDLHSCSFLQLKRKAPSDISTGEYFTVHDMFHELAKTIAGSDCVKIEKSITEHLPKHV

RHLCIESYSEILFPEKILELKNLRTLIMCYSVERMNQDDFERVLKKLTKLRVVHLDLRHL

SRVPPCIGGLKHLRYLGIMSPPPHSLILPAEFSKLYHLQELSVNPNTRLHCPSQLKIANL

INLRYMLTWYGLNIPDVGKLTSLRALYHFYVRKEKGYEIQQLEHLNNLRGKLFIDCIENV

QSKEEAVRARLSDKVYLTELTLRWGGTDERCSKKALESYKKLFFPPVTEIKQHQPPELQE

EVLEGLRPPSGITVLCIRDYGGVIYPSWLTGDGCDKEQEQDRPALQNLMFWSCKGSSDPP

KIGEFFTCLHILSVTDCSWNYLPVKLCRLKTLRELIVQECPNMMTLPKLPQSLKSIVISG

CHPSLADTCLTPGHPNWRRIKHIDQQIIR*

>NIV_RG008

MQGLQSSCPSCCCSSLWSPTMESAAASAFLKTVMGRLFMALEKEYNKHRGLAQESHSLQQ

DLRMIAAAMDDHQLSMGKSDAAARTAVARLHTEEMLDLAHDIEDCVDRFLHRLTCNHHKR

GGAGAGASLLRRVTHELSKVKSRSSFGDEIQKLKKRLREAHQRVLTINPPPILTAGGGQH

TASSSSAAVAPPCRAARSPVGIGEDVEELLSMLDEVEGEPVQMRVVSVVGFGGLGKTTLA

KAVYDDPRAKDKFRHRAWVVAGGSPEIRGILRDVLQQVRPDDAMDVDGQRLEASLKDYLK

DKRYLIVIDDIGMDQWSIISSAFEDNGTSSRIILTTTIQSVANMCSHGSGYVYQMNTLGE

DDSKKLAFPGCRSPELEQGSASLLGKCDGLPLALVSVSDYLKSSSEPTGELCAKLCRNLG

SHLKEKPGHDNFSELRKVLLDNYDSLSGYALSCLLYLGIFPSNRPLKKKVVIRRWLAEGY

ARSDSLHSEEDIADENFSKLIDRHIIQPIDTRNNSEVKTCKTHGIMHEFLLNKSLTQRFI

ATSSHDHPRVGIDTTNARHLSVHAGELTECVASDEELSRVRSLTIFGDAGDAISYFRKCK

LIRVLDLQEWNNLDDDHLKHICKLWHLKYLSFGGNISELPRSIEGLHCLETLDLRRTKIK

FLPIETIMLPHLAHLFGKFMLHKDDLKNVNKMSKLQKFFSSNKSNLRTLAGFITDQGKGF

LQLVRQMKKLRKVKIWCKHVAGSSNYIADLSQAIQEFTRTPIDRDSDRSLSLDCEECSEN

FLSSLDLEPCSEDFKYHLRSLKLHGKLLRLPPFVASLSGLTELCISSAILTQDHLSALIK

LNRLLYLKLIADKLENIDIKIGAFPSLRRLCFVMKSVTSALPTIEQGALPNLVSLQLLCQ

GLVGLSGIEIRHLKHLKEITIDSTVTVQTRQDWEQAAKNHPNRPRVLLFGKVDPMESEEP

EKPCAIGEKRKLSVAQPTGSDGGLDSSLKKMRLSEPSSSRLQVIVHPVVVTATEAAPQHS

FANL*

>NIV_RG009

MEAPTCGLWGAVLNLPGRLDGVLLRHGSILPKGAEEEIPLIKRDLHLMISILNGYYSESP

ELEDATATTMARRRCWTKEVRELSYDIEDCIDHYEHAATAGSAGGRTASGGIPPRRKITR

RRWQRTTPLWIPERLKQRLWMANKIREFSLRTQDALKRHAMFCSSVGGNGIATSTASSST

AATGDASSSSSTICWHTTRFRERDFCVPHVGISVAMNKLEDWLTAYDDEDQKRLSVVSIV

GVGGIGKTTLANELYRKLRRQFECWAFVRSSQKPDVRRILISILSQLRLQQPPESWKVHS

LISIIRAHLQDKRYLIIVDDICFISTWDIIKCALPDGTSSSRVLTTTQYDDLAVQSCGYD

TKYVFKMKSLSQHDSRYLFFNTVSGSRFIYSPGSTEVSDDIINRCGSLPLAIVSITSILE

KSRKMEQWGYVNKSLGYNLMKNPTLEGIKQVLDLRYNNLSEHLKPCVLYLSIYQEDYLIC

KDDLVNQWLAEGLICATKDHTKEEISEACFGELVSSKMIQPVHIDGNGDVMSFVIQHMVL

NFIRYKSIEENFVTVIHHSQTATKLSDKVRRLSLHFGNVKDAKLPINMRLSQVRTLAFFG

AYKYWWRSIKDQFPLLQVLILHFWHDEDIISFDLTIISQLFRLKYLKITSDVTLELQTKT

RGLQCLETLKIDARISTAPLDTTHLSGLLHLSLPADTNLANGIGHMTSLHTFGYFDLSYN

SVENVLSLGKLTNLRNLQLTCSTIRPNSLEIKLQCLGFILKKLSNLKSVTMSTAGSSCVN

STDASSANVSVRISGDGLSSMSSPPALVERLELLPRICIFSYLPKWISLLSKLRILKIGV

RELVRNDIDVLMGLRALTDLSLHVHTKPTEIIFFGGIGFKALKYLKINCCVAWLKFDMGA

MHSLYKLKLGYNVDGVDQERTIPDGIQYLHGLKEISVKIGGADPEKYDRRAEELAFMIDS

GLHDRCMSITLQCVRQMFDFNEDKSSLTHEEQRKLKQQEILEDDSDEEYDEIIQDSGEQE

VEQ*

>NIV_RG010

MESAVASALLKSVMGRLFTVLEKEYSKHRELAQETNSLQQDLRIVAAAMDDQLLSIRRSD

ARTAVARLHSKEMLDLAHDIEDYVDRFIHHLTCRQQCASAGRNSLLDRVAHELKKVQSRS

SFANEIQKLKRRLRQVHQDVIKNNPLAGGQSSSPSPQDRRIADNPVGIEEPVEELLSLLD

EVEGEPERMRVISIVGFGGLGKTTLAKAVYDSPRVKEKFHLRAWVPAGASPETSSGMRGI

LRAVIQKILPNVAMDVDGQLETSLKEYLKDKRYLIVIDDIGMDQWSIISSTFKDNGTSSR

IILTTTIQSIANSCSHGNGYVHQMNTLGEEDCKEIALPTGIRSPELETGSVPLLGKCDGL

PLALVSVSDYLKSSCEPTGELCANLCRNLGAHLKEQDGHPSFSELRKVLLDNYDSLSGYA

LSCLLYLGIFPSNRPLKKKVVIRRWLAEGYARSDSLRNEEDIAVENFNKLIDRNIILPVD

TRNNSDVKTCKTHGIMHEFLLNMSLAQRFIMTLSRDHPRLISNARHLSVHDGELTGYVTS

DEEFSRVRSLTVFGDASDTVSYVRKCKLIRVLDLQECNDFADDHLKHICKLWHLKYLSFG

YNINVLPRSIEGLHCLETLDLRRTKIKFLPIEAVMLPHLAHLFGKFMLHKDDLKNVNKMS

KLNPCKKQKKGMNILPKFFTSKKSNLQTLAGFITGENEGFLQLMGHMKKLRKVKIWCKHV

AGSSNYIADLSQAIQEFTKVPIDSDSNRSLSLDSEECSENFLSALHLEPCSEDFKYHLRS

LKLQGRFLRLPPFVTSLSGLTELFISSATLTQDHLSALITLNRLLYLKLISDKLENFEMK

HGAFPSLRRLCFVVKSVTSDLPTIKQGALPNLVSLHLLCRGLVGLSGIEIRHLKHLKEVV

IDSDVTPQTKQDWAHAAKNHPNRPKFSWPRKVDLVESEEPAKHLKTEKRKYCSNYELDYN

LQEMRLSESRDHKRQKIGEGDTSKSSVGIVYPMYGDVETDRTQVHLFNQETRRYDRTEVD

QKCPEMLQEYKDKHSMVVDVDLRSDEQVNPPHPKLKNLMPGKEYDRQELIPTEGAKVGQC

QSGGDEDQIVHNTNGKKVVVQANHFFEQEDQGSQVTMSYESSSLLSHMDTKSL*

>NIV_RG015

MAVYSVATGALAPVLSKLSALLGDEHLDLAERTRSDAMFIRCQLEAVHSLLLPRISWGMT

GEEVDALCKDELMAEVRELSYDMDDAIDEFFLEEPMAGGDGGPFDELKTRVEDVSKRFSD

SRRWRPPVEQHQPSLTAATVDCPPPHARFVHNMMDVSELVEMDKHEKELIKLLEQGADTS

IYASRWRIATPWHDKEQSIVVKVPEKRRDDMYDDALHWAVSSRLHGVPSGGAYGDYSRLQ

LRGEGANIRKLLSTLRNKVGRAQLVKVEDKRKRVEEATKPCEFHEVKTICILGLPGAGKT

TLAKLLYSHHSTTEQQFQHRAFVSLSPGANLTDTLTDILLQVGTYNDDATPYCGTGTPHQ

QYLIDNISAYLIGKKYLIIIDDVWHWEEWEVIRKSIPKNDLGSRIIMTTRLNSIAEKCRN

DDMDAFVYETEALDYVDAWLLCDKVARKSVTCMNINPCYDIVDMCYGMPLALIRVSSALA

EETQALDSDERQIWRALRRVEDGILDIPSLKPLAESLCLGYDHLPLYLRTLLLCCSVYHW

LDGGIVQRGRLVTRWIAEGFVSEEKAAEGYFDELVGRGWMKHRELNEYEIHPMMLAILRY

KSKEYNFVTCLGTGSDTCTSASLSYSSPTMAIRRLCLQRGYPMKCFSSMDVSHTRSLVIL

GDVIGVPLDMFKRLRVLDLEDNIGIEDSHLKKICEQLESLRLLKYLGLKGTRITKLPQEI

QKLKHLEILYLRSTGIKELPREIGELKQLRTLDVRDTRISELPSQIGELKHLRTLDVSNN

MWNIIELPSQIGELKHLQTLDVRNTLVRELPWQAGQLSESLRVLIDEDDSEEGMQLPKGI

CEDLIKGIPKAELAKCNEVLSIAIADRLGPPSVGIFKVIGSHKCIPKVLKDHFDGLSRLD

IRLCKLDEKDHEFLANNMPNLQMLVLRLQAPQREPIIINCTGFQMLERFLLDSRVPWITF

QEGAMPKLKHLEFKFYAGPPSNDPAVGITHLLSLQNVVFRCSEWYKSDNPGIKATIDVVK

KDARQHPNRPISLLITEGDKEVPNIEAHGSSENIVIVHAAPDDAISCSSCGRTSTSIQEG

TVRDRIPAMDLFWPEFNSYEKAKRN*

>NIV_RG016

MELAVGASEATMRSLLGKLGNLLAQEYSLVSGVRGDIQYINDELASMQAFLRDLSVVTEG

HNHDNRRKDWMKQIRDVAYDVEDCIDDFAHRLPQDSISDAKCSFILTKMYELLTWWPRRD

IASRIAELKVRAQQIADRRNRYGVNNPEHCDSSNSPRPRAHAAAQDIAEYQDTKPQIVSI

KEPVGMKTVMENLEKWLTEPQPDKGRAVLSIVGFGGVGKTTIAMALYRKVSGKFDCQASV

AVSQNYDEDEVLRSILNQVSKQEEAGGSTESSSRDENTREPQGSSSTSSREENTAESGTK

RMLNKLKKALPLSLLGGNDDKTSVRQQETMGSLQLREELKRRLAEKRYILLIDDIWSAKT

WNSIIIPFLPSENDKDSRIIVTTRFHAVGSTCSPRHKNDEATSSPGHGKDLLHKVDFLTG

DKPLDLFNASIPDPMKRTDRDKKLSKICGGLPLAIVTMAGLVACNPNKANSDWSKLCESL

FPYPVTTLNLDGVTRILDCCYNDLPADLKTCLLYLSIFPKGWKISRKRLARRWIAEGFAT

EKQGLTEEEVAEAYFNQLARRNLIRPVEHGSNGKVKAFQVHDMVLEYIMSKSIEENFITV

VGGHWQMTAPSNKVRRLSLQSSGSKHGNSTKGLNLAQVRSLTVFGNLNHVPFHSFNYGII

QVLDLEGWKGLKERHVTEICQMLVLKYLSIRRTEIAKIPSKIEKLEYLETLDIRETYVEE

LPKSVGQLKRISSILGGNKNTRKGLRLPQEKRNKAMKNPSPQGKTKEPAEKGFLSQEKAK

GTMKSLRVLSGIEIVDESAAVAASLHQLTGLRKLAIYKLKISEENDTFKELLSSIEYLGS

CGLQTLAINDENSKFINSLYNMSAPPRYLVSLELSGKLKWLPKWITSITTLNKLTISITV

LTTETLEILRNLPSLFSLTFAFSLSAAKQDQDTVKGILEDNKLATDGEIVIPAKEFKSLK

LLRFFAPFVPKLSFPDKSAMPALEIIEMRFQEFEGLFGIEILENLREVHLKVSDGAEAIT

KFLVSDLKDNTEKPKVFVDGIVTA*

>NIV_RLK002

MALGLLVWIYIVLLIALSTVSAASPPGPSKSNGSETDLAALLAFKAQLSDPLSILGSNWT

VGTPFCRWVGVSCSHHRQCVTALDLRDTPLLGELSPQLGNLSFLSILNLTNTGLTGSLPD

DIGRLHRLEILELGYNTLSALGLNGLTGPLPGNASFNLPALQWFSITRNDFTGPIPVGLA

ACQYLQVLGLPNNLFQGAFPPWLGKLTNLNIVSLGGNQLDAGPIPAALGNLTMLSVLDLA

SCNLTGPIPADIRHLGQLSELHLSMNQLTGPIPASIGNLSALSYLLLMGNMLDGLVPATV

GNMNSLRGLNIAENHLQGDLEFLSTVSNCRKLSFLRVDSNYFTGNLPDYVGNLSSTLQSF

VVAGNKLGGEIPSTISNLTGLMVLALSDNQFHSTIPESIMEMVNLRWLDLSGNSLAGSVP

SNAGMLKNAEKLFLQSNKLSGSIPKDMGNLTSLIQLDLSHNFFSDVLPVDIGNMKQINNI

DLSTNRFTGSIPNSIGQLQMISYLNLSVNSFDDSIPDSFGELTSLQTLDLSHNNISGTIP

KYLANFTILISLNLSFNNLHGQIPKGGVFSNITLQSLVGNSGLCGVARLGLPSCQTTSSK

RKGRMLKYLLPAITIVVGAFAFSLYVVIRMKVKKHQKISSSMVDMISNRLLSYQELVRAT

DNFSYDNMLGAGSFGKVYKGQLSSGLVVAIKVIHQHLEHAMRSFDTECHVLRMARHRNLI

KILNTCSNLDFRALVLEYMPNGSLEALLHSEGRMQLGFLERVDIMLDVSMAMEYLHHEHH

EVALHCDLKPSNVLLDDDMTAHVSDFGIARLLLGDDSSMISASMPGTVGYMAPEYGALGK

ASRKSDVFSYGIMLLEVFTRKRPTDAMFVGELNIRQWVYQAFPVELVHVLDTRLLQDCSS

PSSLHGFLVPVFDLGLLCSADSPEQRMAMSDVVVTLKKIRKDYVKSISTTGSVALPVYRL

CQIDINDRKRGLACIYERMNCPLWPFLLLH*

>NIV_RLK003

MALGSLVCLSALLLIPLSTVSAASSPGLTKSSNNDTDLTALLAFKAQFHDPDNILAGNWT

PGTPFCQWVGVSCSRHQQRVVALELPNVPLQGELSSHLGNLSFLSVLNLTNTGLTGLLPD

DIGRLHRLELLDLGHNAMLGGIPATIGNLSRLQLLNLQFNQLSGRIPTELQGLRSLININ

IQTNYLTGLVPNDLFNHTPSLRRLIMGNNSLSGPIPGCIGSLHMLEWLVLQHNNLTGPVP

PSIFNMSRLTVIALASNGLTGPIPGNTSFSLPALQRIYISINNFTGQIPMGLAACPYLQT

ISMHDNLFEGVLPSWLSKLRNLTGLTLSWNNFDAGPIPAGLSNLTMLTALDLNGCNLTGA

IPVDIGQLDQLWELQLLGNQLTGPIPASLGNLSSLARLVLNENQLDGSVPASIGNINYLT

DFIVSENRLHGDLNFLSTFSNCRNLSWIYIGMNYFTGSIPDYIGNLSGTLQEFRSHRNKL

TGQLPPSFSNLTGLRVIELSDNQLQGAIPESIMEMENLLELDLSGNSLVGSIPSNAGMLK

NAEHLFLQGNKFSGSIPKGIGNLTKLEILRLSNNQLSSTLPPSLFRLESLIQLNLSQNFL

SGALPIDIGQLKRINSMDLSRNRFLGSLPDSIGELQMITILNLSTNSIDGSIPNSFGNLT

GLQTLDLSHNRISGTIPEYLANFTILTSLNLSFNNLHGQIPEGGVFTNITLQSLVGNPGL

CGVAHLGFSLCQTSHKRNGQMLKYLLLAIFISVGVVACCLYVMIRKKVKHQENPADMVDT

INHQLLSYHELAHATNDFSDDNMLGSGSFGKVFKGQLSSGLVVAIKVIHQHLEHAMRSFD

TECRVLRMARHRNLIKILNTCSNLDFRALVLQYMPNGSLEALLHSDQRMQLGFLERLDIM

LDVSLAMEYLHHEHCEVVLHCDLKPSNVLFDDDMTAHVSDFGIARLLLVDDNSISASIPG

TVGYMAPEYGALGKASRKSDVFSYGIMLLEVFTAKRPTDAIFVGELNIRQWVLQAFPANL

VHVVDGQLLQDSSSSTSSIDAFLMPVFELGLLCSSDSPEQRMVMSDVVVTLKKIRKEYVK

SIATMGRDENQTAVFH*

>NIV_RLK005

MGVGPHCTTSLLIILAVVITSSLLTTTIKADEPSNDTDIAALLAFKAQFSDPLGFLRDGW

REDNASCFCQWIGVSCSRRRQRVTALELPGIPLQGSITPHLGNLSFLYVLNLANTSLTGT

LPGVIGRLHRLELLDLGYNALSGRIPPGLAACRKLQMLELGGNLLTDHVPEWLAGLSLLS

TLVIGQNELVGSIPVVLSNLTKLTVLDLSSCKLSGIIPLELGKMTQLNILHLSFNRLTGP

FPTSLGNLTKLSFLGLESNLLTGQVPETLGNLRSLYSLGIGKNHLQGKLHFFALLSNCRE

LQFLDIGMNSFSGSISASLLANLSNNLQSFYANNNNLTGSIPATISNLTNLNVIGLFDNQ

ISGTIPDSIMLMDNLQALDLSINNLFGPIPGQIGTPKGMVALSLSGNKLSSSIPNGVGNL

STLQYLFLSYNRLSSVIPASLVNLNNLLQLDISNNNLTGALPSDLSSFIAIGLMDISVNN

LVGSLPTSLGQLQLSSYLNLSQNTFNDSIPDSFKGLINLETLDLSHNNLSGGIPKYFANL

TYLTSLNLSFNNLQGQIPSGGVFSNITLQSLMGNPRLCGAPRLGFPACLEKSDSTRTKHL

LKIVLPTVIAAFGAIVVFLYLMIAKKMKNPDITASFGIADAICHRLVSYQEIVRATENFN

EDNLLGVGSFGKVFKGRLDDGLVVAIKILNMQVERAIRSFDAECHVLRMARHRNLIKILN

TCSNLDFRALFLQFMPNGNLESYLHSESRPCVGSFLKRMEIMLDVSMAMEYLHHEHHEVV

LHCDLKPSNVLFDEEMTAHVADFGIAKMLLGDDNSAVSASMPGTIGYMAPEYAFMGKASR

KSDVFSFGIMLLEVFTGKRPTDPMFIGGLTLRLWVSQSFPENLIDVADEHLLLDEETRLC

FDHQNTSLGSSSTGRSNSFLMSIFELGLLCSSESPEQRMAMNDVVSKLKGIKKDYSASML

EMQRPRQY*

>NIV_RLK006

MALLLPQNYIHLVILVQLLPLMVLDGFVLGGGGNGTGGDDLSALLDFKAQLSDPLGVLAT

SWTTNASLCRWVGVSCSRRRRRVVELHLRGVPLQGELTPHLGNLSFLRVLDLAAANLTGP

IPANLGRLRRLKILDLAHNTLSDAIPSALGNLTKLETLNLYDNHFSGHVPMELQNLYSLR

VMALDQNYLTVPDSVASLSMLRVLSLPSNQLSGPVPPAIFNMSRLETISIRKNNLTGAIP

TNESFNLPMLRKIDLYMNKFTGPIPSGLASCKHLEMISLGGNLFEDVVPAWLATLSQLKS

LSLGGNELVGPIPGQLGNLSMLNMLDLSFSNLSGPIPVELGTLSQLTFMSLSNNQLNGTF

PAFIGNLSELSHLELAYNQLTGHVPSTIGNNIRHLKHFEIRGNHLHGDLSFLSSLSNSQR

LEVLIISENLFTGCIPNSVGNLSTGILEFRANNNRLIGGLPAILSNLTNLRWINFADNQL

SKPILPASLMTLENLLGFDLSKNSIAGPIPKEISMLTRLVCLFLSDNKLSGSIPDGIGNL

TMLEHINLSNNKLSSIVPTSIFHLNNLILLLLFNNALTGALPSDLSHFQNIDHIDVSDNM

LDGQLPNSYAYHPMLTYLNLSHNSFRDSIPDSFSHLTNLATLDLSYNNLSGTIPKYLANF

TYLTTLNLSFNKLEGEIPTRGVFSNITLKSLRGNAGLCGSPRLGLLPCPDKSLYSTSAHH

FLKFVLPAIIVAIAAVAICLCRMTRKKIERKPDIAGATHYRLVSYHEIVRATENFNDDNK

LGAGSFGKVFKGRLRDGMVVAIKVLNMQVEQAMRSFDVECEVLRMVRHRNLIRILSICSN

LDFKALLLQYMPNGSLETYLHKEGHPPLGFLKRLDIMLDVSMAMEHLHYHHSEVVLHCDL

KPSNVLFDEEMTAHLADFGIAKLLLGDDNSAVSASMQGTLGYMAPEYASMGKASRKSDIF

SYGIMLLEVLTGKRPTDPMFVGDMSLRKWVSDAFPARLLDVLDDRLLQGEILIQQGVLQN

NDTSLPCSATWANEDLLVAVFELGLMCCSNSPAERMEINDVVVKLKRIRKDYLTCTKAI*

>GLA_RG001

IKSFSMATILDSLIGSCVNKLQGIITEEAILILGVEEELRKLQERMKQIQCFISDAERRG

MEDSAVHNWVLTHKEKKAYKLAIIGTGGIGKTTLAQKVFNDQKLKRSFDKHAWICVSQDY

SPASILGQLLRTIDVQYKQEESVGELQSKIESAIKDKSYFLVLDDVWQSDVWTNLLRTPL

YAATSGIILITTRKDTVAREIGVEEPHRVDLMSPAVGWELLWKSINIEDDKEVQNLRDIG

IEIVQKCGGLPLAIKVIAKVLASKDKTENEWKKILANNVWSMAKLPKEITEVHKDQLLED

TGEEYYYELISRNLLQPVVGSFDQRECKMHDLLRQLACYISREECYIGDPTSMVDNNMRK

LRRILVITEKDMVVIPSMGKEEIKLRTFRTQQNPLGIEKTFFMRFMYLCVLDLTDLLVEK

IPDCLGNLIHLHLLDLDGTLISSVPESIGALKNLQMLHLQRCKSLHSLPSAITRLCNLRR

LGIDFTPINKVPRGIGRLQFLNDLEGFPVGGGSDNTKMQDGWNLQELAHLSQLRQLDLNK

LERATPRSSTDALLLTDKKHLKKLNLCCTAPTDEAYSEEGIGNVEMIFEQLTPPRNLENL

MIVSFFGQRFPTWLGTAHLSSVKYVILTDCQSCVHLPPIGQLPNLKYLRIEGASAITKIG

PEFVGCWEGNLRSTEAVAFPKLEMLIIKDMPKWEEWSFVEEEEVQEEEAAAAAQEGGEDG

TAASKQKGEEAPSPTPRSSWLLPCLNELELMGCPKLRGLPPQLGQQATNLKELFIREAKC

LKTVEDLPFLSGFLQVEGCEGLERVSNLPQVRELFVNHCLNLRHVEELGGLEQLWLDEDM

QEISQLWVPGLQEQHRQLHGDEHELEVIEWL*

>GLA_RG002

MATIMGSLIGSCVNKLQGIITEEAILILGVEEELRKLQNRMKQIQCFISDAERRGMEDSA

VHNWISRLKDAMYDADDIIDLASFEGRKRLNDHSCSPRKSTACSGLSLLSCFSNIPVRHE

IGDKIRSLNKKLEEIAKDRIFATLENTQPADKGSTSELRKTSHIVEPNLVGKEIVHACRK

LVSLVVAHKEEKAYKLAIVGTGGVGKTTLAQKVFNDQKLKGTFNKHAWICVSQDYTPVSV

LKQLLRTMEIQHKQEESVGELQSKLESAIKDKGFFLVLDDLWHSDVWTNVLRTPLHAATS

GIILITTRQDTVAREIGVEEAHRVDLMSPAVGWELLWKSMNIQDEKEVKNLRDIGIEIVQ

KCGGLPLAIKVTARVLASKDKTENEWNTILAKNVWSRAKLPKEISGALYLSYDDLPQHLK

QCFLYCILFPEDWFLRRDELIMMWVAEGFVEVHKDQLLEDTAKEYYYELISRNLLQPVDT

SFDQSICKMHDLIRQLACYLSREECYIGDLKPLVDNTICKLRRMLVVGEKDTVVIPCTES

IGSLQNLLMLNLKRCKYLHSLPLATTQLCNLRRLGLAGTPINQVPKGIGRLKFLNDLDGF

PIGGGSDNTKMQDGWNLQDTDALLLTDKKHLKELNLCCTKPTDEEYSEKGISNVEMIFEQ

LTPPRNLEDLMIVSFFGRRFPTWLGITRLPSVKIMILSDCKSCLQLPPIGQLPNLKYLKI

KGASAITKIGPEFVGCWEGNLRSTEAVAFPKLEWLVIKDMPKWEEWSFVEEEEVQEEEAA

AAAQEGGEDGTAASKQKGEEAPSPTPRSSWLLPCLNELELMGCPKLRGLPPQLGQQATNL

KELFIREAKCLKTVEDLPFLSGFLQVEGCEGLERVSNLPQVRELFVNHCLNLRHVEELGG

LEQLWLYKGMQEISQLWVPGLQEQHRQLHGDEHELEVKEWL*

>GLA_RG003

MATILDSLVGSCANKLKEIITEEVILILGIQEELAELQRKTELIHCCISDAEARRMEESA

VDNWLGQLREVLYDVDDIIDLARFKGSILLTDHPSSSSRKSIACTGLSISTCFSNVQASH

EVAVKIRSLNRKIENISKDRVFLTLKSTVPTGSSSVLRVRKSSHLLEPNIVGKEIIHACR

KMVDLVLEHKGRKLYKLAIVGTGGVGKTTLAQKIYNDRKIKGSFNKKAWVCVSKVYSEAS

LLRELLRIMEVHHDQDESIGELQSKLEIAIKETNFLLVLDDMWQSDAWENLLRIPLHAAE

TGTILITTRNNIVALEIGVDHTYRVDLMSTDVGWELLWKSMNISESIELQTLQDVGIEIV

RKCGCLPLAIKVIARVLASKEQTENEWKKILSKNAWFMNNLPNDLRGFIEDHGGQLLEET

ADEYYYELIHRNLLQPDGLYYDHSSCKMHDLLRQLACYLSREESFVGNPESLVGNTVSKL

RRVSVVTDKNMAMLPSMDKVQCKVRTWKTSYEKTLRVDNSFFKRFPYLRVLDLTDSFVPS

IPRCIGNLIHLRLLDLDGTNVSCLPESIGNLKNLQILNSLPSAITQLCNLRRLGLNYSPI

DQVPKGIGKLEFLNDVEGFPVYGGSSNTKMQDGWNLEELAHLYQLRRLHMIKLERAAYST

TYPLLTDKGFLKFLYLWCTERTDEPYTEKDFGNIEKIFEQLIPPCNQEDLAIVKFFGRQY

PFWIDSTHLAYVKSLHLFKCKFCMHLPPVGQLPNLKYLKIEGAAAVTIIGPEFAGRRVSN

LGRTVAFPKLEELLIRDMPNWEEWFFIDEATSTAKERVDDGDSAMPKEKTLPPRMQILSR

LRRLELSGCPKLKALPRQLAQINSLKEIELRWVSSLKVVENFPLLSETLLIATCQALEKV

SNLPQVRELRVQDCPNLRLVEELGTLEQLWLYEDMHEVSTLWVQGLQQQCRQCHGEDLDV

YNWT*

>GLA_RG005

MATILDSFIGSCAKKLQEIITEEAILILGVKEELRELQERMEQIRCFVSDAENRGMDDSA

IHNWISRLKDAMYDADDIIDLASFEGSKLLNGHSSPPRKTTACSGLSLLSCFSNIQIRHE

VGEKIRSLNKKIEKIEKDNIFATLGNTEPADKGSTSELRKRSNVVEPNIVGKEIVHACRK

LVSLVLTHKEGKAYKIAIVGTGGIGKTTLAQKVYNDQKLKDSFSRRAWICVSKEYSPVHL

LRQLLRTMEVHYAQDELLEELQTKLALAIKNKSFFIVLDDLWQSDVWTNLLRTPLHAASS

GIIVVTTRYDTVALEIGVELIHRVDLMSLDVGWELLWKSMNILEEKEVQNLWDIGIEIVQ

KCGGLPLAIKVVARVMASKDKTESEWRKILTRNVWSMTKLPKEISGGLYLSYDDLPQHLK

QCFLYCIVFPEDWVFDRDDLIRMWVAEGFLEVQKDQLLEDTAEEYYYELIHRNLLQPVGT

YFDQSKCKMHDLLRQLACYLSREECYIGDPTSLVDNTIYKLRRILVITEKDMVVIPSMGK

EEIKLRTFTTDKQPRAIDNTLFMRLSYLRVLDLSDSLVQTIPDYVGNLIHLRLLNLDGTN

ISCLPESIGSLQNLQTLNLQRCESLHSLPLATTQLCNLRRLGLELTPINLVPNGIGRLKF

LNDLNGIPIGCGSNNTKMQVGWNLQELAHLSQLRRLYLDKLERATPCSGTESLLLTDKIH

LKVLMLSCTEQTDEEYSEEDVSNVEKIFEHLTPPHNLEDLFIGAFFGRRFPTWLGTTHLS

SVKFLILEDCKSCMHLPPMGQLPNLKYLRIDGAKEITKIGPEFIGCGVGNLRCTEAVAFP

KLEWLIINDMPNWEEWSFVEQEEEEVATAAKEGGDDGAAALFPRLSWLMPCLTKLDLIGC

PKLRALPPQLGQQATNLKGILICGASSLKTVEDLWFLSYAIHVQECGDLERISNLPHVRV

MYARDCPNLRCVEELGSLEQLWLYEDMQEISSLWVPRLREQHNQHHEDELEVNEWFPY*

>GLA_RG006

MATILGSLVGSCVNKLQGIITEEAILIIGVKEELRKLQERMKQIQCFINDAERRGMEDSA

VHNWISRLKDVMYDADDIIDLASFEGNKLLNGHSSSPRKRTASSALSPLSCFSNIRVRHE

IGDKIRTLNRKLAEIEKDKIFATLENTQPADKGSTSELRKTSHIVEPNLVGKEIVHACRK

LVSLVVAHKEDKAYKLAIVGTGGIGKTTLAQKVFNDQKLKGTFNKHAWICVSQDYTPVSV

LKQLLRTMEVQHAQEESAGELQSKLELAIKDKSFFLVLDDLWHSDVWTNLLRTPLHAATS

GIILITTRQDIVAREIGVEEAHRVDLMSPAVGWELLWKSMNIQDEKEVQNLRDIGIEIVQ

KCGGLPLAIKVTARVLASKDKTENEWKRILAKNVWSMAKLPKEISGALYLSYDDLPQHLK

QCFLYCIVFPEDWTLDRDELILMWVAEGFVEVHKDQLLEDTAEEYYYELISRNLLQPVYT

YFDQSRCKMHDLLRQLACYLSREECYIGDLKPLVDNTICKIRRMLVVGEKDTVVIPFTGK

EEIKLRTFTTDHQLQGVDNTFFMRLTHLRVLDLSYSLVQTIPDYIGSLIHLRMFNLDGTN

ISCLPESIGSLQNLLILNLQWCKYLHVLPLATTQLYNLRRLGLTDTPINQVPKGIGRLKF

LNDLEGFPIGGGSDNTKMQDGWNLEELAYLPQLRKLGMIKLERGTPRSSPDPFLLAEKKH

LKVLELQCTEQTDESYSVENVSNIEQIFEKLTPPHNLEKLVIVNFFGCRFPTWLGTAHLP

LVKSVILVDCKSCVHLPSIGQLPNLKYLRIEGASAISNIGPEFVGCWEGNLRSTEAVAFP

KLELLVIEDMPNLEEWSFVEEEEEEEEEEEEEEEEEEAQEEDASAAAKEAGENGTCASKE

EGALSPTPRSLWLLPCLTRLELDDCPKLMALPRLLGQQATNLKGLVIRHASCLKTVEDLP

FLSFLSIGGCEGLERVSNLPQVRELLVGGCPNLWHVEMLGSLEQLWLDKDMQEISQLWVP

RLQQQHRQLHGDEHELEVNEWL*

>GLA_RG007

MATILDSLVGSCANKLKEIITEEVILILGIQEELAELQRKTELIHCCISDAEARRMEESA

VDNWLGQLREVLYDVDDIIDLARFKGSILLTDHPSSSSRKSIACTGLSISTCFSNVQASH

EVAVKIRSLNRKIENISKDRVFLTLKSTVPTGSSSVLRVRKSSHLLEPNIVGKEIIHACR

KMVDLVLEHKGRKLYKLAIVGTGGVGKTTLAQKIYNDRKIKGSFNKKAWVCVSKVYSEAS

LLRELLRIMEVHHDQDESIGELQSKLEIAIKETNFLLVLDDMWQSDAWENLLRIPLHAAE

TGTILITTRNNIVALEIGVDHTYRVDLMSTDVGWELLWKSMNISESIELQTLQDVGIEIV

RKCGCLPLAIKVIARVLASKEQTENEWKKILSKNAWFMNNLPNDLRGALYLSYDELPRHL

KQCFLYCSVYPEDANIYRDDLTRMWIAEGFIEDHGGQLLEETADEYYYELIHRNLLQPDG

LYYDHSSCKMHDLLRQLACYLSREESFVGNPESLVGNTVSKLRRVSVVTDKNMAMLPSMD

KVQCKVRTWRTSYEKTLRVDNSFFKRFPYLRVLDLTDSFVPSIPRCIGNLIHLRLLDLDG

TNVSCLPESIGNLKNLQILNSLPSAITQLCNLRRLGLNYSPLDQVPKGIGKLEFLNDVEG

FPVYGGSSNTKMQDGWNLEELAHLYQLRRLHMIKLERAAYSTTYPLLTDKGFLKFLYLWC

TERTDEPYTEKDFGNIEKIFEQLIPPCNQEDLAIVKFFGRQYPFWIDSTHLAYVKSLHLF

KCKFCMHLPPVGQLPNLKYLKIEGAAAVTIIGPEFAGRRVSNLGRTVAFPKLEELLIRDM

PNWEEWFFIDEATSTAKERVDDGDSAMPKEKTLPPRMQILSRLRRLELSGCPKLKALPRQ

LAQINSLKEIELRWVSSLKVVENFPLLSETLLIATCQALEKVSNLPQVRELRVQDCPNLR

LVEELGTLEQLWLYEDMHEVSTLWVQGLQQQCRQCHGEDLDVYNWT*

>GLA_RG010

MSDEAPSGISRKKLKKSLEKIENIINEAHRILPLLSLPNQGNVNKRQIVHANSRSPVTTA

TPPPVVIGREKDCDNIISMLHEHVSNVQPGSSNSVLCYSIIGIYGIAGSGKSTLAQLVCA

SEKKDKQEKKDGHFDLIMWVHVSQNFSVDTILTEMLEAATGKKCDRFNNLDTLEQKLEEA

LSGKRFLLVLDDIWYHNSENQHEQQKILTPLRVGKPGSKVLVTSRTEYALLALGALKCIP

ISDLDDNVFLKLFLHYALPLVNMDERDQRKLEVIGANIAKKLRRSPLAARTVGGQLQIRP

NVDFWRDACNRDLLNETMGALWWSYQHLDEQVRRCFSYCSIFPRRHQLKRDELVNLWVAE

GFISTTDTGEEEAAAQQYFGELVSSSFLNKHMGEYSSEKNVYFTVHDLLHDLAEKVAGSD

CFRIQQDWAGVFPRNVRHLFIEAYDETIITERILEMENLRTLVIHCISRDMMDHETVYES

IFMKLRKLRVLKVQSDALWRQYTGFSVLSLPASIKKLKHLRYFGYQSGYVYKLVLPSTIS

TLHHFQVLDFGNCMELVFSSEEDLSKLNSLRHVIVSPLFPLSIRHLGRLTSLQTMPPIKV

ERGEGYELQQLRRLNKLRGRLEIQGLENVESKEAAAEANLGAKECLQQLVLVWEDDNESC

SPDVQEEVLEGLCPPMELESLEIKGYQGSRYPSWLVVQQNCGPNYLHELRLSGCSRMGSI

PALTHLCSLTIFNCSWDSLPDDMERLTSIKNLTLSHCYNILLLPTLPKSLELLRVDGCST

ELTSSCRTTGHPNWHKIKHIPRKYM*

>GLA_RG012

MADLVSISTAVGWVITPIISKLINRGFSYLNFDASEKLNRIQSKVVQLELMLEQQGSFGA

FVQGAERSGSTSIDAHMPHVLKMELKKILEKIENTVNEAHQILKLPDLFGVSDGKERQRV

AVTTSVPPPVVIGRDEDRDKIIAMLHEKIDNGQRKANGGLSYSVIGIHGIPGSGKSTLAQ

HVCASEKKGKREEKDGHFNLIMWIHVSRNFSVDKIFREILEEATGNPCPRFESLDILEQK

LEEALSGKHFLLVLDDVWYREGVNQQELQKVISPLKVGDLGSKILLTSRSRDALLALGAI

RCIPISDLDDNVFLQLFLHYALEGAEIDERERTIFEEIGSDIAKKLKRSPLAARTVGGQL

RIRPNADFWRDTRDRGLLDATTGALWWSYQHLDEQIRRCFAYCSIFPRRHRLKRDKLINL

WVAEGFISTTNAGEEMEDVGQRYFDELLSASFLQLVEKQTGALKMLAKNDCFRIENGWTG

VIPRDVRYLFIETYNKTIVMDKILQIENLRTLIIYSDKLDMLIEENVLESMFTRLRKLRV

LRVRTVTQSGTLSFPASIGQLKHLRYLSFWTSSSVKQVLPRTFSKLYHMQLLDTGHCRNI

VFPSGEDTVNLINLRHIVTFENQNFPDIRKLTSLQTLSFFTVKKGLGYDLQQLTHLNKLR

GRLQIHGLENVQNKEQAVAANLAAKERLKELILVWDGDNCSPDIETEVIEGLCPPMEIER

LELRNYHGLRYPHWMVRKQNGLKNLRELKLYKCGHLGPPPELFEFFYHLCVLSLSQCSWD

ALPDNMEHLNSLQKLNIFECQNIRSLPMLPQSLLHFELRGCDQHFTRSCKTIGHPNWQKI

QHIPSKEVEQIYSQEYLRSYHEVPKI*

>GLA_RG013

MPDPVTAGAAVGWGISALGWFVSPIISKLLSRCFSYLGFDGPQKLGEFQSKVFQLELILK

DVEVHPPSNRLEELLDKLKSAFYEAEDILDDIEHHRLERQILYPHGRKWVNMLQSAIPMC

SCLTNQDTTLHDISKMELEKILNKIENNINEALKILALLPSYSKDKERQTVAVNSRIEVT

TAAPPPVVIGRDMDHDKIIGMLHEKVDYGQRKIRSGALCYSVIGIHGIPGSGKSTLAQLV

CASERKKRHFKPIMWVHVSKHFSVDTILSEMLEAATGNPCPEFKNRDTLQQKLEEALTGE

RFLLVLDDVWCEKNSGQLELQKIVSPLKVGGSGSKILATSRTADALLALDAMRCIPIADM

DDDVFLQLFMHYALEGADIDERDRAILKEIGSGIAKKLRRSPLAASIVGRQLRMRLDADF

WRDASKRDLLKETMGALLWSYQQLDEQVRRCFAYCSIFPRRYRLKRDELINLWVAEGFIG

STDAGEEMEDVGGKYLDELVSASFLQQAGQQRAVSGAVDYFIVHDLLHDLAERVNRSDCF

RIENGWTGVLPPNVRHLFVETYNKAMISDKILEMESLRTLIIYRHKQDIPFDKFMRLKKL

RVLSMLSVTTSNVFSFPASIGRLKHLRYLAFRTGRVKLVFPSTFTNLYHLQVLDIGGCKD

LVFSSGEDINLLNLRHVISSADLNFRNIGSLTSLQTLSFFTAKKGPGYELRQLKHLSKLR

GRLEMHGLENVQSKEEALEANLASKEHLKKLALVWDKDTCCPEVQAEVLEGLYPPPELEK

LEITNYHGSRYPDWMVGKQKGPKNLHILSLSSCSGLGSSQKLSEFVNLRSLGLYMCSWNA

LPDNMEQLTSLQTLVIYKCQNICSLPMLPRSLVQFVLRGCNPEFRRSCETIGDPNWQKIQ

HIANKEFDSNQVDVPKTETAWKVHLYTPLLPRFPICICRMLRLSLRVVLTMFTPYLGSLQ

SLQLNQMSPAPPNSPDSPHRGR*

>GLA_RG015

MADPVTVSAAVGWGISAVGWVVSPIITNLINKGFSYLDLGSRGRSAKLNELENKVSELRR

VLEAVEESSVRDRLEPLLDRLKSAFYEAEDILDDVEYYHLERQILFQPDDKFRRNWMKKL

QSALPECSCLKKQKSTGRSSTSTSAPPDVSRLALKRILDKIENIINEAHKVLPLTNLPGY

TRADIVSSSTRSAVTTATPPPVVIGRDKDRDDIIAMLHENVDDIQPGSGSIIGIHGIAGS

GKSTLAQLVCASEKNADHFDLIMWVHVSQNFSVRTILTEMLEAATGKQCDNLNNVDILER

NLEAELRRHRFFLVLDDIWYDGENQQELQKILAPLKAGKAGSKILATSRNMDALLALGAG

RCILISDMDDNVFLKLFMHYALEGANIDEQDRRIFEVIGVDIAKKLRKSPLAARTVGGQL

RIRLNIDFWRDARDRDLLNQTMGALFWSYQHLGEQVRRCFSYCSIFPRRHRLKRDELVTL

WVAEGFIRTSDQGEEEAVGRQYFDELVSSSFLMKQTGGGPTNYFTVHDLLHDLAEKVAGS

DCFRIEKGLQGVIIPPGVRHLSIETFDKEMITEKISEMENLHSLIIHGSDIDMIADETLF

ESMFTRMRKLRVLGVKTFGFKQRRELSFPNSISNLKHLRYFGFATGSTSTCKLVLPRTMT

KLYHLQVLDFGYCRELVFSSEEDLCRLTNLRHFIASDQELNIPNFGRLTSLQTIPLLTVT

KEAGCEIQQLAQLNKLRGRLQIEGLENVESKEAAAGANLAAKKGLKELVLVWDWNDESCS

PDVQAEVLEGLCPPMELETLEIRGYRCSMYPSWLVGQLLNLNNGPKFLNQLKLNRCSPLG

SIPQQSELFTDLRSLVIWCCSWDSLPDNMERLKSLEKLELFLCPQILLLPALPLSLKTFR

LYRCSDVLTSSCQTIGHENWQKIKHIQNKHL*

>GLA_RG016

MGSAITVLGWLLSPIISLLVNRFISYLFDASPKIQELEIQTVPKLEQMPRKIEEERMHRK

AKKERSAVQNLDTLAKLVKSALYEAEDILDLIGYHQIEKDVIGDDEPQGSSSKWHPHIDE

AIHACKTSWIGRCITTLLEWAQSLHRSLRSRSAALLPISCSRCCGSASDSLLERLSCLSG

QFDFIRCCQSLFIWSVNWFEVARSYRDWFYDATGITATGYQLEDGTAVYSFMPAIARWKL

RKRIEKLENTVTNVEKSPYLTQTSSGAWNDIVNMNRRSITSSSTRKVFGRDRERDMIRSM

LREDDSLPSSSSRKCYSVICIYGIPGSGKTTLAQLEDIFRDMLEEITLSRHSEISDCRGL

EGKLVENLRGKRFLLVLDDLWVNDENHEKLLSPLNVGKSGSRILVTAQSKEAALGSNRLI

PISDLEEEQYFSMFMHYALDSTIFDDREYIPIGRKIAKKLNRSPIAAVTVAGQLWRNPDI

RFWQTTANLDVLNKTKGALWWSYNQLVVDVRRCFQYCSIFPRRYELERDNLVRMWIAQGF

VKDNDGNNEDVEDVGQDYFHDLHSCSFLQLKRKAPSDISTGEYFTVHGMFHELAKTIAGS

DCVKIEKSITEHLPKHVRHLCIESYSEILFPEKILELKNLRTLIMCYSVEGMNQDDFERV

LKKLTKLRVVHLDLRHLSRVPPCIGGLKHLRYLGIMSPPPHSLILPAEFSKLYHLQELSV

HPNTRLHCPSQLKIANLINLRYMLTWYGLNIPDVGKLTSLRALYHFYVRKEKGYEIQQLE

HLNNLRGKLFIDCIENVYLTELTLRWGGTDERCSKKALESYKKLFFPPVTEIKQHQPPEL

QEEVLEGLRPPSGITVLCIRDYGGVIYPSWLTGDGCDKEQEQDRPALQNLMFWSCKGSSD

PPKIGEFFTCLHTLSVTDCSWNYLPVKLCRLKTLRELIVQECPNMMTLPKLPQSLKSIVI

SGCLPSLADTCLTPGHPN*

>GLA_RG017

MFNLPGRLEELLCHHGSMLPKGADEEIPLIKQDLEEIISILHGHSEPKLEDHGMVVRCWM

KEVRELSYDIEDSIDQYEHAARSQNRPNIHHRKFNRWRGNKIPCIPQKLKQRLWMANKIR

EFSLRAQEALQRHAMYNNLGGVASTASTTRGDVCSATPLHPTQTQFREHVDNVRSVSIEA

DGMEASLNDLNKLKNLFAGIPTASLVQFREHADKVRGIHTDIEAILNKLENIPPGITTTT

TTRGDVSSTSSRQPTRFMESTCHVGIDAAMDQLENLIDVCGEEKLKVVSIVGVGGVGKTT

LANKLYRKLRWQFECRAFVRTSQKTDMTRLLINILSQVRPHQSPDNWKVHSLISSIRSYL

QDKRFLIVIDDLWATSTWDIIKCALPEGNKSSRILTTTEIEDLALQSCSYDLKFIFKMKA

FGEDDSRKLLFNIVFGSHSKCPPEVSETLYDIVRKCGGLPLAIVTVASLLASQLDKQEQW

DYINKSLGFSLMANPTLEGMKQLLNLCYNNLPQHLKACMLYLSMYQEDHIIWKDDLVSQW

IAEGFICATEGHDKEEISRAYFDELIGRKIIQPVHINDSGEVLSCVVHHMVLNFVTYKSI

EENFIIAIDHSQATIRFADKVRRLSIHFGNVEDAPPPTNMRLSQVRTVAFFGVLKYMPFI

LEFQLIKVLVLHFLGDEDSIGIFDLTKISELIRLRYLKVTSNVTVKLPTQMQGLQYLETL

KIDGKISEVPSDIIHLPGLLHLTLPAKTSLPNGIAHVTSLRTIGYFDLSCNSVENLWHLG

ELTNLRDLQLTYCEIHSDNLKDNMKYLGSILGKLRNLISITLSPPGSSCPDTLYIDRDTK

TRINVDGWSSVSSPPALLQRFELLPCVCIFSNLPNWIGQLGNLCILKIGIREVTSNNIDV

LGVLPELTVLSLYVHTKPAERIVFDNAGFSILKYFKFICSVACMKFEMGAMPSLRKLKLG

FDVHRADQHDIIPVGVEHLSGLEEISAKIRVACSAHDHCKRFAESALSNAFMMHPGRPSV

NIRCVDWTFDGKDDDNVRTQEEEHWTLQKQHHTAKEGSNEKSPVKQRDPREGAHKSVDGR

ETLRRIRVKVSSTVDDGFSWVKYGQKDILGTMYPRSYFRCIHRQTKGCLATKQVQPTDDD

HQILDVIYYGEHTCDQSARSDDRQLKSSRPAASSNLQEPQQPGLEQSRPAAKRRRKTMRW

KTQVRVSSVQDVGPLDDGYSWRKYGLKDILGAKYPRSYFRCTHRNTQGCVATKQIQRRDG

DPLLFDVVYHGDHTCSERASLNEQVTWPRSSASSTEQSQSSSTITYTAAAGSVEDDEEDV

TSAINFLSMDDMLDLGGGEVIDMDFPSFDLDAIEAFLS*

>GLA_RG018

MESAAASAFLKTVMGRLFMALEKEYNKHRGLAQESHSLQQDLRMIAAAMDDQQLSMGKSD

AAARTAVARLHTEEMLDLAHDIEDCVDRFLHRLTCNHHKRGGAGAGASLLRRVTHELSKV

KSRSSFGNEIQKLKKRLREAHQRVLTINPPPILTAGGGQHTASSSSAAVAPPCRAARSPV

GIGEDVEELLSMLDEVEGEPVQMRVVSVVGFGGLGKTTLAKAVYDDPRAKDKFRHRAWVA

AGGSPEIRGILRDVLQQVRPDDAMDVDGQRLEASLKDYLKDKRYLIVIDDIGMDQWSIIS

SAFEDNGTSSRIILTTTIQSVANMCSHGSGFVYQMNTLGEDDSKKLAFPGCRSPELEQGS

ASLLGKCDGLPLALVSVSDYLKSSSEPTGELCAKLCRNLGSHLKEKPGHDNFSELRKVLL

DNYDSLSGYALSCLLYLGIFPSNRPLKKKVVIRRWLAEGYARSDSLHSEEDIADENFSKL

IDRHIIQPIDTRNNSEVKTCKTHGIMHEFLLNKSLTQRFIATSSHDHPRVGIDTTNARHL

SVHAGELTECVASDEELSRVRSLTIFGDAGDAISYFRKCKLIRVLDLQEWNNLDDDHLKH

ICKLWHLKYLSFGGNISELPRSIEGLHCLETLDLRRTKIKFLPIETIMLPHLAHLFGKFM

LHKDDLKNVNKMSKLQKFFSSNKSNLRTLAGFITDQGKGFLQLVRQMKKLRKVKIWCKHV

AGSSNYIADLSQAIQEFTRTPIDRDSDRSLSLDCEECSENFLSSLDLEPCSEDFKYHLRS

LKLHGKLLRLAPFVASLSGLTELCISSAILTQDHLSALIKLNRLLYLKLIADKLENIEIK

IGAFPSLRRLCFVMKSVTSALPTIEQGALPNLVSLQLLCQGLVGLSGIEIRHLKHLKEIT

IDSTVTVQTRQDWEQAAKNHPNRPRVLLFGKVDPMESEEPEKPCAIGEKRKLSVAQPTGS

DGGLDSSLKKMRLSEPSSSRLQVIVHPVVVTATEAAP*

>GLA_RG019

MEAPTCGLWGAVLNLPGRLDGVLLRHGSILPKGAEEEIPLIKRDLHLMISILNGYYSESP

ELEDATATTMARRRCWTKEVRELSYDIEDCIDHYEHAATAGSAGGRTASGGIPPRRKITR

RRWQRTTPLWIPERLKQRLWMANKIREFSLRAQDALKRHAMFCSSVGGNGIATSIASSST

AATGDASSSSSTICWHTTRFRERDFCIPHVGINVAMNKLEDWLTACDDEDQKRLRVVSIV

GVGGIGKTTLANELYRKLRRQFECWAFVRSSQKPDVRRILISILSQLRLQQPPDSWKVHS

LISSIRAHLQDKRYLIIVDDICFISTWDIIKCALPDGTRSSRVLTTTQYDDLAVQSCGYD

TKYVFKMKSLSQHDSRYLFFNTVSGSRFIHSPGSTEVSDDIIRKCGSLPLAIVSITSILA

KSRKMEQWGYVNKSLGYNLMKNPTLEGIKQVLDLSYNNLSEHLKPCVLYLSIYQEDYLIC

KDDLVNQWLAEGLICATKDHTKEEISEACFGELVSSKMIQPVHIDGNGDVMSFVIQHMVL

NFIRYKSIEENFVTVIHHSQTATKLSDKVRRLSLHFGNVKDAKLPINMRLSQVRTLAFFG

AYKYWWRSIKDQFPLLQVLILHFWHDEDIISFDLTIISQLFRLKYLKITSDVTLELQTKT

RGLQCLETLKIDARISTAPLDTTHLSGLLHLSLPADTNLANGIGHMTSLHTFGYFDLSYN

SVENVLSLGKLTNLRNLQLTCSTIQPNSLEIKLQCLGFILQKLSSLKSVTMSTAGSSCVN

STDASSTNISVGISGDGLSSMSSPPALVERLELLPRICIFSYLPKWISLLSKLRILKIGV

RELVRNDIDVLMGLRALTDLSLHVHTKPTESIFFGRLGFKALKYLKINCCVAWLKFDMDA

MPSLYKLKLGYNVDGVDQERTIPDGIQDLYGLKEISVKIGGADPEKYDRRAEELAFMIDS

GLHDRCMSITLQCVRQMFDFNEDKSSLTHEEQRKLKQQEILEDDSDEEYDEIIQDSGEQE

VKQ*

>GLA_RG020

MESAVASALLKSVMGRLFTVLEKEYSKHRELAQETNSLQQDLRIVAAAMDDQLLSIRRSD

ARTAVARLHSKEMLDLAHDIEDYVDRFIHHLTCRQQCASAGRNSLLDRVAHELKKVQSRS

SFANEIQKLKRRLRQVHQDVIKNNPLAVTTSSGGQSSSPSPQDRHIADNPVGIEEPVEEL

LSLLDEVEGEPERMRVISIVGFGGLGKTTLAKAVYDSPRVKEKFHLRAWVPAGASPETSS

GMRGILRAVIQKILPNDALDVDGQLETSLKKYLKDKRYLIVIDDIGMDQWSIISSTFKDN

GTSSRIILTTTIQSIANSCSHGNGYVHQMNTLGEEDCKEIALPTGIRSPELETGSVPLLG

KCDGLPLALVSVSDYLKSSCEPTGELCANLCRNLGAHLKEQDGHPSFSELRKVLLDNYDS

LSGYALSCLLYLGIFPSNRPLKKKVVIRRWLAEGYARSDSLRNEEDIADENFNKLIDRNI

ILPVDTRNNSDVKTCKTHGIMHEFLLNMSLAQRFIMTLSRDHPRLISNARHLSVHDGELT

GYVASDEEFSRVRSLTVFGDASDTVSYVRKCKLIRVLDLQECNDFADDHLKHICKLWHLK

YLSFGYNINVLPRSIEGLHCLETLDLRRTKVKFLPIEAVMLPHLAHLFGKFMLHKDDLKN

VNKMSKLNPCKKQKKGMNILPKFFTSKKSNLQTLAGFITGENEGFLKLMGHMKKLRKVKI

WCKHVAGSSNYIADLSQAIQEFTKVPIDSDSNRSLSLDSGECSEDFLSALHLEPCSEDFK

YHVRSLKLQGRFLRLTPFVTSLSGLTEIFISSATLTQDHLSALITLNRLLYLKLIADKLE

NFEIKHGAFPSLRRLCFVVKSVTSDLPTIKQGALPNLVSLHLLCRGLVGLSGIEIRHLKH

LKEVVIDSDVTPQTKQDWARAAKNHPNRPKFSWPRKVDLVESEEPAKHLETEKRKYCSND

ELDYNLQEMRLSESRDHKRQKIGEGDTSKSSVGLVYPMYGDVGTDRTQVHLSNEETRRYD

RTEVDEKCPEMLQECKDKCSMVVDVDLRSDEQVNPPHPKLKNLMPGKEYDRQELIPTEGA

KVGQCQSGGAEDQIVHNTNCKKVVAQANHVFEQEDQGSQVTMSYESSSVTHGY*

>GLA_RG024

MEFAAASALLKSVMGRLFMALEKEYSKHKGLAQETHSLQQDLRMIAAAMDDQLRVLGRSD

ARNAVARLHTEEMLDLSHDIEDCVDRFLHRLTCNHKRGSGSNGAGPGGASSMVRRMAHEL

SKVQSRSSFADEIQKLKRRIREAHQRVMDIKSIVDVIAGGQPTTGAMSSSATAPCRNTCN

PVSIGEPVEELLSLLDEVEGEPEQMRVISVVGFGGLGKTTLARAVYDSPGAKGKFSHRAW

VTIGTSPERDSGILHALLQQVLPKDAIGVDGQHDLEALLKEYLKDKRYLIVMDDINMEQW

SIIRSTFVDNGTSSRIILTRTIQSVANMCSHGNGYVYKMNTLGEEDSKTLAFPGFRSPEL

EQGSESLLGKCDGLPLALVSVSDYLKSSSEPTGELCAKLYRNLGSHLKERDGHYSFSELR

KVLLDSYDSFSGYALSCLLYLGIFPNNRPLKKKVVIRRWLAEGYARSDSLRREEDIADEN

FSKLIDQNIVQPVDTRNNSEVKTCKTHGIMHEFLLNKSLSQIFIAKSSRDHPRLGINTNA

RHLSVHAGELTESVESDEELSRVRSLTIFGDAGHAICYVRKCKLIRVLDLQECNDLDDEH

LKYICKLWHLKYLSFGSNISELPRSIEGLHCLETLDLRRTEIKFLPIEAIMLPHLAHLFG

KFMLHRDDVNSVNKMTKLQKFFSSKKSNLQTLAGFITDESKGFLQHIGHMKKLRKVKIWF

KHVTGSSNYIADLSQAIQEFTKAPIDRDIDRSLSLDSEECPENFLSSLDLETCSEGSKYA

LRSLKLNGELHRLPPFVTLLSGLTELCISSATLTQGHLSALINLNRLLYLKLVAYKLVNF

EIKHGAFPSLRRLCFVVKSVTSALPTIEHGALPNLISLQLLCQGLVGLSGIEIKHMKYLK

EVTINSGVAIQWEQAAKNHPNRPKILILRKVNPMESEEPERPCAIREQRKISVAQTTSLD

DGLDSSLNKMRLSKPSSSRLQVFVHPVVITATEAAPQSSLANL*

>GLA_RG025

MSEAIALAMSKIKTCLETEASFKNAMFKLSKKDKLVKELLEKIKQIEEQLDITNDFIQEQ

ISTVNLGDPLDESWIATVRRLAFIVEDVMEKYLYYAHQLQEEGSQKHPVKRSSYVDVFNK

VGQVMDKINYHIGHLLSIREQSKLAPQLVPYSPYPDSEGQPELIGTNGPFNVLLKFLKHG

EDEAVLQPKVLSMLGIGGIGKTALVSKVYRMLREEFQRHASVTVSKMPDMKRVLFDVLDQ

IGLRKYPTSLNENQVIAQIGQDLKDKRYLIVIDDLWDMQHWKTIKDALPDANYGSRLIVT

TRLNNIAETCSSGQHDLTYKVMPLAHQDSRTLFLKEILGHEGSCSDAPVFDEILKMFGGM

PSALKCIGSFLRNKLVTTESQKIMMSSLHSELENFPSWQKLKKSLFLSCCGPSQTLEVCS

LYLSTLPDNHKIERGILTRKWISEGIILKENDLSINGVANKCFEELINRNVIQQVDNSFG

EETYEIHFLMHHVLRQIARERNFATFLSDNILISCKEPIHRLSFHCSKLRISIDKGDIQI

ISDSGDSNKKPKSLSLARSITLCGYAKPVSFKLLEHLHVLDLEGCWNVDNSSLDDICRMI

LLQYLSLKKTRITVLPPQIENLRGLKTLAVTQTEIAELPLQIGKLPDLETLDVRHTQVKE

IPKELVQLRKLVCLLFGQSGFHGGVKFPVGGNPSKSLKVLGAIDSTQCSASFMGELSSLT

GLTELSVVCYDGTKGKECNLRMMNSIFKFSNLESLTIYGDFILGNEVPALQNPPKLQKLK

VAGRCLSVPGWIDKFSNVTLLDIRVCSLEESDLKILCKMSSLQRLVLTQVHMPIKQLEIT

KEASFSKLNGFTFDCRVPWVTFKEEAMPSLQYLELKLYAGPAGKIPSGITCLPCLTKVIL

RYSAHYQSSASVQDTISKMRKESSEHPNMIVLSQNGEHEIFPQNAVSRNGEHEIVLVNAV

DGMHQD*

>GLA_RG027

MGRESIDVVTGALGSVLPKLKLLLEDEFTMELEMIYAALHDVANSRAEQQGRNKLWALEL

RELSYDVEDAVDTILVRLGGLESSTEAASSTGSCWLIKITKRATTHKVFDEIKDIRLRVK

EVNEWRDRYMIDDSLHNPRVSAIYDPPRLPADLSVDQHSLVGIDQAAAELIEMLALEGGA

FERRLKTVSIVGMGGLGKTTLAKLVYSMLKDQFQFGAFVSVSQCPDMNKVFQDMFYQLNE

GNYDHADKDVEQLILAKSDSLRKKRYFIIIDDIWSMEAWNMIGSALLKNENGSRVITTTR

IFKVASSADDVYTMRPLSPENSKRLLRKRICTGEDNSDGVELAEVCDKLLKKYDGLPLAV

LTIADALLDVEPINKQCYEHVKGRVMHDMIHIFSLSYHDLPPHLRTCLLYLSIFPEDYLI

KKDFLIWRWIAEGFIEYDGGISLFEVGESYFEELIDRSMIQPVEAGDEDSVDGCRVHGAV

LDLLCYLATEENFVTLLIDNEQNISLGRGKPRRLALQKGNNEDHTLQTDISLAKVRSFNA

SMCSANMMPLLSGFKFLRVLALEHCPSIKSYHLKYVGKLFLLRYLGLVGTPISELPDGIG

ELVFLQTLDLRETGIQELPRSICRLRKLMCLCVDSTAILPSGIGNLVALEDLRLYSVSTL

HFVKEELGQLTKLRILEIRFEELDEQMEDAFLRSLSNLQNLQTLEAPELRLGDLKILGRL

PSLRSLWISSRSNERPLVITVEDGFPSLIEFTLLNGAFGPDFQRGAMPKVRRVEFSFSLR

DFSSRADFGFGLENLLSLEHVTIRLHDKVHSVEAALRHLTKKHPRRPTITLIRDGEEPTG

SYGLTLI*

>GLA_RG028

MAVYSVATGALAPVLSKLSALLGDEHSERTRSDAMFIRCQLEAVHSLLLPRINLGMTGED

VDALCKDELMAAVRELSYDIDDAIDDFFLEGGDGSPFDDELKTRVDDVSKRFSDSRYWRP

PVEQHQPSLTAATVDSPPPHARFVHNMMDVSELVEMEQHLKELIKLLEQGADTSTYASRW

RTATPWHDKEQSIVVKVPEREWGGPNHPFRWARDSFERFRSGSLFGDTLQLDGEGANIRK

LLSTLRNKAGHAQLVQVEDKGKKVEETTTMPCEFHEVKTVCILGLPGAGKTTLAKLLYSH

HSTTEQQFQYRAFVSVSPGANLTETLTDIFLQVGADNDATPYCGTGTPHQQCLIDNISAY

LIAKKYLIIIDDVWRWEEWEVIRKSIPKNDLGSRIIMTTRLNSIAEKCRNDDIDAFVYET

EALDYLDAWLLCDRVARKSVTCMNINPCYYIVDMCYGMPLALICVSSALAEEIQALAGDD

ERQKWRALRLVEDGILDIPSLKPLAESLCRGYDHLPLYLRTLLLCCSVYHWLDGGIVERG

RLVTRWIAEGFVSEEKEAEGYFDELVGRGWIKHREWNEYEIHPMMLAILRYKSKEYNVVT

CLGMGSDTSTSASLSSYSSPTMAIRRLCLQRGYPMKCFTQSSPSMDVSHTRSLVILGDVT

GVPFDMFKRLRVLDLEDNLDIEDSHLKKICEQLESLRLLKYLGLKGTRITKLPQEIQKLK

HLEILYVGSTCIEELPQEIGELKHLRTLDVACTEIKKFPLHIREIQHLRVGSTRIEELPQ

EIGELKHLRTLDVRNTDVRELPSQIGELKHLQTLDVRNTEVRELPWQAGQIWESLRVLTD

DSEEGMQLPKGVREDLIKGIPEADLAKCREVLSIPIVDRLVSPPVGIFKVIGLRKCIPEV

FKDYFDVLSCLDIWLWKLEEEDHEFLANNMPNLQMLVLRFEAPQREPIIINNTGFRMLER

FHVDSRVPRITFQEGAMPKLKHLEFKFYAGPPSNDPAVGITHLLSLQKVVFRCSKWYKSD

NPGIKATIDVVKKEARQHPNQPISLLITEGDKEVPNEAHGSSENSACRRHWQQWAARPPH

CSKLRHQLL*

>GLA_RG029

MPLNYPQTHLLISLSLPPPTSGPPGGGRQWCSNRAGLAAHGGGGRCTGGCVWPVMGSASG

TTHGRTGGASEDDDRAAGVLRVARARTNPCHVSQPPVDVSAAKIFRALPRPRKLSAGVQC

LISTYCVCLTHTLQCLQELTIAAKSTEDKMELVVGASEATMRSLLGKLGNLLAQEYALIS

GVRGDIQYINDELASMQAFLRDLSVLPEGHYHDNRSKDWMKQIRDIAYDVEDCIDDFAHR

LPQDSISDAKCSFLVTKMYELLTWWPRRDIASRIAELKVRAQQIADRRNRYGVNNPELHC

DSSNRPRARSAAQDIAEYQVTKLQIIGIKEPVAMKTVMEDLEKWLTEPRPDNGRAVLSIV

GFGGVGKTTIAMALYRKVSGKFGCRASVAVSQNYDQDAVLRSILNQVSNQEEAGSSTESS

RDTTASREENTAEPGTKSTFIKLKKAPSLSLLGRNDDKTPVRQQETMDYVQLRQELKRRL

GEKRYILLIDDIWSANTWLDVVIPFLPDEANNNSRIIVTTRFHAVGSTCSRGHKNVEATS

SPRPGNNRVHKNVEATSSPRPGNDRVHKNVEATSSPRPGNDRVHKNVEATSSPRPGNNRV

HTVDFLSDDESQNLFNASIPDSMKSADRDKELSSICGGLPLAIVTMAGLVACNPNKTDCD

WSKLCKSLFPDPVTTLNLDGWQVTAPSNKVRRLSMQSSGSKNGNSTKGLNLAQVRSLTVF

GNLNHVPFRSFNYGIIHVLDLEGWKGLKERHMTEICQMLVLKYLSIRRTEIAKIPSKIEK

LEYLETLDIRETDVKELPKSVGQLKRIHSILGGNKKTREGLSLLQEKGNKTIKNPLPQGK

TKEPAKKGFLSQEKSKGIMKSLHVLSGIEIDEKSAAVATSLHQLTGLRKLAIYKLNIEWG

SEVFQELRSSIEYLGSCGLQTLAINDENSNFIDSLDDMSAPPRYLIALELSGMLYRLPEW

ITSITTLNKLTISITVLMTKTLEILRSLPSLFSLTFAFSLSAAKQDLDKIKDILENNKLA

TDGEIVIPAEGFKSLKLLRFFAPFVPKLSFSDKNAMPALEIIEMRFKDFEGLFGIEILEN

LRGVHLKVSDGAEAITNFLASDLKDNTEKPKVFVDGIVTA*

>GLA_RLK001

MALGSPVCIIVSALLLITLSPVAAAAAASPPGPSKSNGNDTDLAALLAFKGELSDPYNIL

ATNWTAGTPFCRWMGITCSRRQQQRVTGVELPGVPLQGKLSPHIGNLSFLSVLNLTITNL

TGSIPDDIGRLHRLELLDLGNNAFSGVIPASIGNLTRLGVLRLAVNQLSGQIPADLQGLH

SLRSINIRNNGLTGSIPNSLFNNTPLLSYLNIANNSLSGSIPACIGSLPMLQFLDLQVNQ

LAGPVPPGVFNMSMLGVIALALNGLTGPIPGNESFRLPSLWFFSIDANNFTGPIPQGFAA

CQQLQVFSLIQNLFEGTLPSWLGKLTNLVKLNLGENHFDGGSIPDALSNITMLASLELST

CNLTGTIPADIGKLGKLSDLLIARNQLRGPIPASLGNLSALSRLDLSTNLLDGSVPSTVG

SMNSLTYFVIFENSLQGDLKFLSALSNCRKLSVLEIDSNYFTGNLPDYVGNLSSTLQAFI

ARRNNISGVLPSTVWNLTSLKYLDLSDNQLHSTISESLMDLEILQWLDLSENSLFGPIPS

NIGVLKNVQRLFLGTNQFSGSISMGISNMTKLEYLDLSDNQLASTVPPSLFHLDRLVKLD

LSHNFLSGALPADIGYLKQMNIMDLSSNHFTGILPDSIAQLQMIAYLNLSVNSFQNSIPD

SFRVLTSLETLDLSHNNISGTIPEYLANFTVLSSLNLSFNNLHGQIPETGVFSNITLESL

VGNSGLCGAVRLGFSPCQATSPKRNHRIIKYLVPPIIITVGAVACCLYVILKYKVKYQKM

SVGMVDMASHQLLSYHELARATNDFSDDNMLGSGSFGKVFKGQLSSGLVVAIKVIHQHME

HAIRSFDTECRVLRTARHRNLIKILNTCSNLDFRALVLEYMPNGSLEALLHSDQRIQLSF

LERLDIMLDVSMAMEYLHHEHCEVVLHCDLKPSNVLFDDDMTAHVSDFGIARLLLGDDSS

MISASMPGTVGYMAPEYGALGKASRKSDVFSYGIMLLEVFTAKRPTDAMFVGELNIRQWV

LQAFPANLVHVIDGQLVQDSSSSTGSIDGFLMPVFELGLLCSSDSPEQRMVMSDVVVTLK

KIRKEYVKSIATMGRDENRTAVFH*

>GLA_RLK005

MGLVPPWMTLLVLVIMSSTSITVAEHHRRSSNDTDLAALLAFKAQLSDPHGVLGDGWRDN

VSFCHWIGVSCSRRRQRVTALVLPDTLLDGSITPHLGNLSFLTVLSHTNTSLTGSIPAEL

GRLARLRYLDLRRNTLSGSIPSTMGNLTRLQSLIFDRNHLSGKIPSELQNLQNLVHLSLQ

GNYLSGSIPDLIFNGSSSVLTFINLGNNSLSGPIPSVISSLSMLQVLVLQFNQLSGSLPP

TIFNMSRLEELYSAGNNLTGPITFPAGNQSFICPMIRTLGIGGNRFTGRIPPGLTACRKL

ETLGLCLNLLSGDVPEWLADLSELNFLYLCENELTGSIPAVLSNLTMLTELDLSFCNLTG

RIPTELATLTQLTYLYLESNQLTGPFPTFVCNLTRLSLLALDTNLLTRQVPETLGNLRSL

HWLDIGENHLQGKLDFFASLSNCRQLQILDIGNNSFSGSIPASLLANLSNNLVFFNAGEN

SLTGNLPDTISNLTNLNVISLSNNQLSGPIPDSIVLMENLQYSSLHGNIMFGPVPTQIGT

LQSIVVLYLDDNKFSGSIPNGVGNLTTLQDLRLSYNLLSSSIPASLVNLSNLLRLYISHN

NLTGALPSDLSPWRAIAEMGISANNLVGSLPTSWGHLQLLSYLNLSQNTFNDLIPDSFKG

LVNLETLDLSHNNLLGGIPKYFANLTFLTSLNLSFNNLQGQIPSGGVFSNITLQSLMGNP

RLCGVPRLGFPACLEKSHSTRTKHLLKIVLPAIIAAFGAIVVFLYLLIGKKMKNPDITAS

FDIADAICHRLVSYQEIVRATENFNEDNLLGVAIKILNMQVERALRSFDAECHVLRMARH

RNLIKILNTCSNLDFRALLLQFMPNGNLESYLHSESRPCVGSFLKRMEIMLDVSMAMEYL

HHEHHEVVLHCDLKPTNVLFDEEMTAHVADFGIAKMLLGDDNSAVSASMPGTIGYMAPGI

CTSQSKATYMRKASRKSDVFSFGIMLLEVFTGKRPTDPMFIGGLTLRLWVSQSFPDNLID

VADEHLLQDEETRLCFDYQNISSTSRSNSFLTLIFELGLLCSSESPEQRIAMNDVVSKLK

GIKKDYSASMLEMQRPRQY*

>GLA_RLK008

MALGLLVWIYIVLLIALSTVSAASPPGPSKSNGSETDLAALLAFQAQLSDPLGILGGNWT

VGTPFCRWVGVSCSHHRQRVTALDLRDTPLLGELSPQLGNLSFLSILNLTNTGLTGSVPD

DIRRLHRLEILELGYNTLSGSIPATIGKLTRLQVLDLQFNSLSGPIPADLQNLQNLSSIN

LRRNYLTGLIPNNLFNNTHLLTYLNIGNNSLSGPIPGCIGSLPILQTLVLQVNNLTGPVP

PAIFNMSTLRALALGLNGLTGPLPGNASFNLPALQWFSITRNDFTGPIPVGLAACQYLQV

LGLPDNLFQGAFPPWLGKLTNLNIVSLGGNQLDAGPIPAALGNLTMLSVLDLASCNLTGP

IPADIRHLSQLSELHLSMNQLTGPIPASIGNLSALSYLLLMGNMLDGLVPATVGNMNSLR

GLNIAENHLQGDLEFLSTVSNCRKLSFLRVDSNYFTGNLPDYVGNLSSTLQSFVVAGNKL

GGEIPSTISNLTGLMVLALSDNQFHSTIPESIMEMVNLRWLDLSGNSLAGSVPSNAGMLK

NAEKLFLQSNKLSGSIPKDMGNLTKLEHLVLSNNQLSSTVPPSIFHLSSLIQLDLSHNFF

SDVLPVDIGNMKQINSIDLSTNRFTGSIPNSIGQLQMISYLNLSVNSFDDSIPDSLGELT

SLQTLDLSHNNISGTIPKYLANFTILISLNLSFNNLHGQIPKGGVFSNITLQSLVGNSGL

CGVARLRLPSCQTTSPKRNGRMLKYLLPAITIVVGAFAFSLYVVIRMKVKKHQMISSGMV

DMISNRLLSYHELVRATDNFSYDNMLGTGSFGKVYKGQLSSGLVVAIKVIHQHLEHAMRS

FDTECHVLRMARHRNLIKILNTCTNLDFRALILEYMPNGSLEALLHSEGRMQLGFLERVD

IMLDVSMAMEYLHHEHHEVVLHCDLKPSNVLLDDDMTAHVSDFGIARLLLGDDSSMISAS

MPGTVGYMAPEYGALGKASRKSDVFSYGIMLLEVFTGKRPTDAMFVGELNIRQWVYQAFP

VELVHVLDTRLLQDCSSPSSLHGFLVPVFELGLLCSADSPEQRMVMSDVVVTLKKIRKDY

VKSISTAGSVALPAYTKE*

>BAR_RG001

MATILDSLIGSCVNKLQGIITEEAILILGVEEELRKLQERMKQIQCFISDAERRGMEDSA

VHNWVSWLKDAMYDADDIIDLASFEGSKLLNGHSSSPRKSFACSGLSFLSCFSNIRVRHK

IGDKIRSLNQKLEEIAKDKIFATLENTKSSHKVSTSELRKSSQIVEPNLVGKEILHACRK

LVSQVLTHKEKKAYKLAIIGTGGIGKTTLAQKVFNDQKLKRSFDKHEWICVSQDYSPAST

LGQLLRTIDVQYKQEESVGELQSKIESAIKDKSYFLVLDDVWQSDVWTNLLRTPLYAATS

GIILITTRKDTVAREIGVEEPHRVDLMSPAVGWELLWKSINIEDDKEVQNLRDIGIEIVQ

KCGGLPLAIKVIARVLASKDKTENEWKKILANNVWSMAKLPKEITGALYLSYDDLPQHLK

QCFLYCIVYPEDWTIHRDYLIRMWVAEGFVEVRKDQLLEDTGEEYYYELISRNLLQPVVG

SFDQRECKMHDLLRQLACYISREECYIGDPTSMVDNNMRKLRRILVITEKDMVVIPSMGK

EEIKLRTFRTQQNPLGIEKTFFMRFMYLRVLDLTDLLVEKIPDCLGNLIHLRLLDLDGTL

ISSVPESIGALKNLQMLHLQRCKSLHSLPSAITRLCNLRRLGIDFTPINKVPRGIGRLQF

LNDLEGFPVGGGSDNTKMQDGWNLQELAHLSQLRRLDLNKLERATPRSSTDALLLTDKKH

LKKLNLCCTAPTDEAYSEEGIGNVEMIFEQLTPPRNLEDLMIVSFFGQRFPTWLGTAHLS

SVKYVILTDCQSCMHLPPIGQLPNLKYLRIEGASAITKIGPEFVGCWEGNLRSTEAVAFP

KLEMLIIKDMPNWEEWSFVEEEEVQEEEAAAAAQEGGEDGTAASKQKGEEAPSPTPRSSW

LLPCLNELELMGCPKLRGLPPQLGQQATNLKELFIREAKCLKTVEDLPFLSGFLQVEGCE

GLERVSNLPQVRELFVNECPNLRHVEMLGSLEQLWLDEDMQEISQLWVPGLQEQHRQLHG

DEHELEVIEWL*

>BAR_RG005

MATILDSFIGSCAKKLQEIITEEAILILGVKEELRELQERMEQIRCFVSDAENRGMDDSA

IHNWISRLKDAMYDADDIIDLASFEGSKLLNGHSSPPRKTTACSGLSLLSCFSNIQIRHE

VGEKIRSLNKKIEKIEKDNIFATLGNTEPADKGSTSELRKRSNVVEPNIVGKEIVHACRK

LVSLVLTHKEGKAYKIAIVGTGGIGKTTLAQKVYNDQKLKDSFSRRAWICVSKEYSPVHL

LRQLLRTMEVHYAQDELLEELQTKLALAIKNKSFFIVLDDLWQSDVWTNLLRTPLHAASS

GIIVVTTRYDTVALEIGVELIHRVDLMSLDVGWELLWKSMNILEEKEVQNLWDIGIEIVQ

KCGGLPLAIKVVARVMASKDKTESEWRKILTRNVWSMTKLPKEISGGLYLSYDDLPQHLK

QCFLYCIVFPEDWVFDRDDLIRMWVAEGFLEVQKDQLLEDTAEEYYYELIHRNLLQPVGT

YFDQSKCKMHDLLRQLACYLSREECYIGDPTSLVDNTIYKLRRILVITEKDMVVIPSMGK

EEIKLRTFTTDKQPRAIDNTLFMRLSYLRVLDLSDSLVQTIPDYVGNLIHLRLLNLDGTN

ISCLPESIGSLQNLQTLNLQRCESLHSLPLATTQLCNLRRLGLELTPINLVPNGIGRLKF

LNDLNGIPIGCGSNNTKMQVGWNLQELAHLSQLRRLYLDKLERATPCSGTESLLLTDKIH

LKVLMLSCTEQTDEEYSEEDVSNVEKIFEHLTPPHNLEDLFIGAFFGRRFPTWLGTTHLS

SVKFLILEDCKSCMHLPPMGQLPNLKYLRIDGAKEITKIGPEFIGCGVGNLRCTEAVAFP

KLEWLIINDMPNWEEWSFVEQEEEEVATAAKEGGDDGAAALFPRLSWLMPCLTKLDLIGC

PKLRALPPQLGQQATNLKGILICGASSLKTVEDLWFLSYAIHVQECGDLERISNLPHVRV

MYARDCPNLRCVEELGSLEQLWLYEDMQEISSLWVPRLREQHNQHHEDELEVNEWFPY*

>BAR_RG008

MATILGSLVGSCVNKLQGIITEEAILIIGVKEELRKLQERMKQIQCFINDAERRGMEDSA

VHNWISRLKDVMYDADDIIDLASFEGNKLLNGHSSSPRKRTASSALSPLSCFSNIRVRHE

IGDKIRTLNRKLAEIEKDKIFATLENTQPADKGSTSELRKTSHIVEPNLVGKEIVHACRK

LVSLVVAHKEDKAYKLAIVGTGGIGKTTLAQKVFNDQKLKGTFNKHAWICVSQDYTPVSV

LKQLLRTMEVQHAQEESAGELQSKLELAIKDKSFFLVLDDLWHSDVWTNLLRTPLHAATS

GIILITTRQDIVAREIGVEEAHRVDLMSPAVGWELLWKSMNIQDEKEVQNLRDIGIEIVQ

KCGGLPLAIKVTARVLASKDKTENEWKRILAKNVWSMAKLPKEISGALYLSYDDLPQHLK

QCFLYCIVFPEDWTLDRDELILMWVAEGFVEVHKDQLLEDTAEEYYYELISRNLLQPVYT

YFDQSRCKMHDLLRQLACYLSREECYIGDLKPLVDNTICKIRRMLVVGEKDTVVIPFTGK

EEIKLRTFTTDHQLQGVDNTFFMRLTHLRVLDLSYSLVQTIPDYIGSLIHLRMFNLDGTN

ISCLPESIGSLQNLLILNLQWCKYLHVLPLATTQLYNLRRLGLTDTPINQVPKGIGRLKF

LNDLEGFPIGGGSDNTKMQDGWNLEELAYLPQLRKLGMIKLERGTPRSSPDPFLLAEKKH

LKVLELQCTEQTDESYSVENVSNIEQIFEKLTPPHNLEKLVIVNFFGCRFPTWLGTAHLP

LVKSVILVDCKSCVHLPSIGQLPNLKYLRIEGASAISNIGPEFVGCWEGNLRSTEAVAFP

KLELLVIEDMPNLEEWSFVEEEEEEEEEEEEEEEAQEEDASAAAKEAGENGTCASKEEGA

LSPTPRSLWLLPCLTRLELDDCPKLMALPRLLGQQATNLKGLVIRHASCLKTVEDLPFLS

FLSIGGCEGLERVSNLPQVRELLVGGCPNLWHVEMLGSLEQLWLDEDMQEISQLWVPRLQ

QQHRQLHGDEHELEVNEWL*

>BAR_RG010

MQGLTTPSCSTVALFHCCVCGILVLHFYQLWARCLIRISRVYFCLHCDRLRRDRVILISN

LLRNFGASPSTSFSMATILGSLIGSCVNKLQGIITEEAILILGVEEELRKLQKRMKQIQC

FISDAERRGMEDSAVHNWVSWLKDAMYDADDIIDLASFEGSKLLNGHSSSPRKTTACGGL

SPLSCFSNIQVRHEIGDKIRSLNRKLAEIEKDKIFATLKNAQPADKGSTSELRKTSHIVE

PNLVGKEILKVSRNLVCHVLAHKEKKAYKLAIVGTGGIGKTTLAQKLFNDQKLKGSFNKH

AWICVSQDYSPSSVLRQLLRTMEVQHRQEESVGELQSKLELAIKDKSYFLVLDDVWQHDV

WTNLLRTPLYAATSGIILITTRQDIVAREIGVEKQHRVDQMSPADGWELLWKSISIQDEK

EVQNLRDIGIKIIQKCGGLPLAIKVIARVLASKDKTENEWKRILDKNVWSMAKLPKEIRD

CIGYLIHLRLLDLDRTCISCLPESIGALKNLQMLHLHRCKSLHSLPTAITQLYNLRRLDI

VETPINQVPKGIGRLKFLNDLEGFPVSGGSDNAKMQDGWNLEELADLSKLRWLIMINLER

GTPHSGVDPFLLKEKKYLKVLNLWCTEQTDEAYLEENANNVENIFEMLTPPHNLRDLVIG

NFFGCRFPTWLGTTHLPSVKSVILANCKSCVHLPPIGQLPNLNYLKIIGASAITKIGPEF

VGCREGNLISTEAVAFPKLEMLIIKDMPNWEEWSFVDQQEEEEVQEEEEAAAAAKEGGED

GTVASKQKGKVALSPRSSWLMPCLRRLDLWDCPKLRALPPQLGQTNLKELLIRYTSCLKT

VEDLPFLSGLLLVERCEDLERISNLPQVRELFLNCCPNLRHVEELGGLEQLWLDEDMHEI

SQQWVPGLQEQRCKLHGDEHELVVNEWL*

>BAR_RG014

MADLVSISTAVGWVITPIISKLINRGFSYLNFDASEKLNRIQSKVVQLELMLEQQGSFGA

FVQGAERSGSTSIDAHMPHVLKMELKKILEKIENTVNEAHQILKLPDLFGVSDGKERQRV

AVTTSVPPPVVIGRDEDRDKIIAMLHEKIDNGQRKANGGLSYSVIGIHGIPGSGKSTLAQ

HVCASEKKGKREEKDGHFNLIMWIHVSRNFSVDKIFREILEEATGNPCPRFESLDILEQK

LEEALSGKHFLLVLDDVWYREGVNQQELQKVISPLKVGDLGSKILLTSRSRDALLALGAI

RCIPISDLDDNVFLQLFLHYALEGAEIDERERTIFEEIGSDIAKKLKRSPLAARTVGGQL

RIRPNADFWRDTRDRGLLDATTGALWWSYQHLDEQIRRCFAYCSIFPRRHRLKRDKLINL

WVAEGFISTTNAGEEMEDVGQRYFDELLSASFLQLVEKQTGAFSAVYYYTVHDLLHDLAD

KIAKNDCFRIENGWTGVIPRDVRYLFIETYNKTIVMDKILQIENLRTLIIYSDKLDMLIE

ENVLESMFTRLRKLRVLRVRTVTQSGTLSFPASIGQLKHLRYLSFWTSSSVKQVLPRTFS

KLYHMQLLDTGHCRNIVFPSGEDTVNLINLRHIVTFENQNFPDIRKLTSLQTLSFFTVKK

GLGYDLQQLTHLNKLRGRLQIHGLENVQNKEQAVAANLAAKERLKELILVWDGDNCSPDI

ETEVIEGLCPPMEIERLELRNYHGLRYPHWMVRKQNGLKNLRELKLYKCGHLGPPPELFE

FFYHLCVLSLSQCSWDALPDNMEHLNSLQKLNIFECQNIRSLPMLPQSLLHFELRGCDQH

FTRSCKTIGHPNWQKIQHIPSKEVEQFYSQEYLRSYHEVPKI*

>BAR_RG015

MPDPVTAGAAVGWGISALGWFVSPIISKLLSRCFSYLGFDGPQKLGEFQSKVFQLELILK

DVEVHPPSNRLEELLDKLKSAFYEAEDILDDIEHHRLERQILYPHGRKWVNMLQSAIPMC

SCLTNQERCHSWKEAAQRGSIEGGRGLKGQPTTVGGTQGTQSEWVDCFFQLIAKDTMGWM

REATLLQSISGPAAPVSYQPHEVATSRQGKGVPDWQDCLDSKGAPEIADPTDTTLHDISK

MELEKILNKIENNINEALKILALLPSYSKDKERQTVAVNSRIEVTTAAPPPVVIGRDMDH

DKIIGMLHDKVDYGQRKIRSGALCYSVIGIHGIPGSGKSTLAQLVCASERKKRHFKPIMW

VHVSKHFSVDTILSEMLEAATGNPCPEFKNRDTLQQKLEEALTGERFLLVLDDVWCEKNS

GQLELQKIVSPLKVGGSGSKILATSRTADALLALDAMRCIPIADMDDDVFLQLFMHYALE

GADIDERDRAILKEIGSGIAKKLRRSPLAASIVGRQLRMRLDADFWRDASKRDLLKETMG

ALLWSYQQLDEQVRRCFAYCSIFPRRYRLKRDELINLWVAEGFIGSTDAGEEMEDVGGKY

LDELVSASFLQQAGQQRAVSGAVDYFIVHDLLHDLAERVNRSDCFRIENGWTGVLPPNVR

HLFVETYNKAMISDKILEMESLRTLIIYRHKQDIPFDKFMRLKKLRVLSMLSVTTSNVFS

FPASIGRLKHLRYLAFRTGRVKLVFPSTFTNLYHLQVLDIGGCKDLVFSSGEDINLLNLR

HVISSADLNFRNIGSLTSLQTLSFFTAKKGPGYELRQLKHLSKLRGRLEMHGLENVQSKE

EALEANLASKEHLKKLALVWDKDTCCPEVQAEVLEGLYPPPELEKLEITNYHGSRYPDWM

VGKQKGPKNLHILSLSSCSGLGSSQKLSEFVNLRSLGLYMCSWNALPDNMEQLTSLQTLV

IYKCQNICSLPMLPRSLVQFVLRGCNPEFRRSCETIGDPNWQKIQHIANKEFDSNQVDVP

KTETAWKSLQLNQMSPAPPNSPDSPHRGR*

>BAR_RG018

MANPWKVAAMGATISICGWFISPIISLVVNKIISYIGFDWSGELEKLKRERVPKLKELLS

NAEVHRMLAESRNDVDADHVARLEDMVNRLRSALYEADDILDLVEYYRLKKKVLGESGSS

DSSTWLQLLHGYSRRVHNALLVPAAPVLRRWYAAMRACVARTKIALRLPTSTSVRGRRGL

LNSLRNCGGYILTCPSKVIMAARSLRSFFCDAATELFNLNYQQANDTMSRGIPPDMSKLR

LKIILNKIENIICEADKQLPHLDKPPNKVTDAIPPPAVIGRDNDRDNIIAMLHENVDGVQ

PGSSNSVLSGRSIIGIHGISGSGKSTLAQHICDHLKKDKQEENDCHFDLIMWVHVSENFD

VVDIFSKILKEATKLNTEEHDLEKALSEKRFLLVLDDVWSVKVESHQSKEMKDKIVSLLK

AGKAGSKILATSQTDKALLALGAAKERCILISEMGDTVFLELFMHYALDGVDIDEQDRRA

FEVIGAQIANKLKGSPLAATIVGARLRQENLNYWISFNDQKHLGDVMDFLKWSYQQLDEE

ARRCFAYCSIFPRRYQLQRGELVKLWVAQGFITTTNGDKKEIDAERHFDHLVSVLFLRPV

NDDYSGHIAYFTMHDLLYDLAKMVVGSECYTVQKGWAGDLPQNVRHLFIEIDGEENMSKK

VLELENLRTLIIIHTQDTEKLLVEDVLECMFTNLRMLRALILKVELQSFKGGRQIFIPES

IGELLHLRYFYLSKALYSKVIFPRRFARLYHMEVVNVGYSEWGKVEFSSGEDTTNLINLR

VVPYDAEFDFPFVGRMESLQTLHKFTVKRETGYELFQLKKLNKLKHLLKIDGLENVPTKE

DAHEAELHNKSYIRGLKLTWNPSGETSVQDQDLQSEVIQALRPPTHLQTLAIESYNGSSY

PSWMMDGGPEVLMCLRDLMLIDCTELGSVPEQSVLFKYLHSLQIIRCNWRYFPDNMEHLK

SLVELVIQNCSEILSLPTLPQSLQQFKIKNCDAILSLPTLPQSLKELITVGCYKLELLPT

LPPSLQKLKTKYCNAILSLPTLPQSLKELIIVGCKNLESLPTLPQSLKELMIEDCKNINS

LPTLPQSLQKLMLYRFRNIDLPTMPQSLELFDVLTNDQKFARSCETVGEENWEKIRHIPT

RNINYDEYYVQGNNFNPFSSHLFTWITEVLLILTYTCCFRHKLTQTFVLNIHL*

>BAR_RG019

MANPWRIAAMGSAITVLGWLLSPIISLLVNRFISYLFDASPKIQELEIQTVPKLEQMLRK

IEEERMHRKAKKERSAVQNLDTLAKLVKSALYEAEDILDLIGYHQIEKDVIGDDEPQGSS

SKWHPHIDEAIHACKTSWIGRCITTLLEWAQSLYRSLRSRSAALLPISCSRCCGSASDSL

LERLSCLSGQFDFIRCCQSLFIWSVNWFEVARSYRDWFYDATGITATGYQLEDGTAVYSF

MPAIARWKLRKRIEKLENTVTNVEKSPYLTQTSSGAWNDIVNMNRRSITSSSTRKVFGRD

RERDMIRSMLREDDSLPSSSSRKCYFVICIYGIPGSGKTTLAQLEDIFRDMLEEITQSRH

SEISDCRGLEAKLVENLRGKRFLLVLDDLWVNDENHEKLLSPLNVGKSGSRILVTAQSKE

AALGSNRLIPISDLEEEQYFSMFMHYALDSTIFDDREYIPIGRKIAKKLNRSPIAAVTVA

GQLWRNPDIRFWQTTANLDVLNKTKGALWWSYNQLVVDVRRCFQYCSIFPRRYELERDNL

VRTWIAQGFVKDNDGNNEDVEDVGQDYFHDLHSCSFLQLKRKAPSDISTGEYFTVHDMFH

ELAKTIAGSDCVKIEKSITEHLPKHVRHLCIESYSEILFPEKILELKNLRTLIMCYSVKG

MNQDDFERVLKKLTKLRVVHLDLRHLSRVPPCIGGLKHLRYLGIMSPPPHSLILPAEFSK

LYHLQELSVHPNTRLHCPSQLKIANLINLRYMLTWYGLNIPDVGKLTSLRALYHFYSKEE

AVRARLSDKVYLTELTLRWGGTDERCSKKALESYKRLFFPPVTEIKQHQPPELQEEVLEG

LRPPSGITVLCIRDYGGVIYPSWLTGDGCDKEQEQDRPALQNLMFWSCKGSSDPPKIGEF

FTCLHTLSVTDCSWNYLPVKLCRLKTLRELIVQECPNMMTLPKLPQSLKSIVISGCLPSL

ADTCLTPGHPNWRRIKHIDQQIIR*

>BAR_RG020

MEGSMFNLPGRLDRLLLRHGSMLPKGAEEEIPLIKQDLEEIISILHGHCSEPKLENHAMV

VRCWMKEVRELSYDIEDCIDQYEHATTATRSRTGPNIRRRKFNQRHGKMIPGVPWKLKQR

LWMANKIREFSLRTQEALQRHTMYNNLGGITIASTTGGDACSATPWHPTHFREHTDNIRS

VGIDADGMEAALNDLNKLKNLLASIPTASLEQFREHANKVCHIHPDMEAILNKLKNIPPG

ITTTSTTTRGDVSSTSSRQPMRFMESAGLVGIDAAVNKLENLLDVCGEEKLKVVSIVGVG

GVGKTTLANKLYCKLQRQFECRAFVQTSQKTDMRRLLINILSQVQPHQSPDNWKVHSLIS

SIRTHLQDKRYLIIIDGLWATSTWDVIKCALPDGNSSSRILTTTEIEDLALQSCSYDLKF

IFKMKPFGEGDSRKLFFSIVFGSHSKCPPEVSETLYDIVRKCGGLPLAIVTVASLLASQL

EKQEQWDYINKSLGYGLMANPTLEGMKQLLNICYNNLPQHLKVCMLYLSMYQDHIIWKDD

LVSQWIAEGFICATEGHDKEEISSAYFDELVGRKIIQPVHIDDSGEVLSCVVHHMVLNFV

TYKSIEENFIIAIDHSQATIRFADKVRRLSIHFSNVEDATPPTNMRLSQVRTVAFFGVLK

YMPFVMEFRLIKVLVLHILGDEDSIGIFDLTKISELVRLRYLKVTSNVTIKLPTQMQGLQ

YLETLKIDGTISEVPTDIYLPGLLHLTLPAKTNLPSGIVHMTSLRTIGYFDLSCNSAENL

WSLGELSNLRDLQLTYSEIHSDNLKDNMKYLGFILGKLRNLTSITLSPPGSSCPDNLHID

RDTKTRINVDGWSSVSSPPALLQRFELLPCVCIFSNLPNWIGQLGNLCILKIGIREVTSN

NIDVLGVLPELTVLSLYVHTKPAERIVFDNAGFSILKYFEFICSVAWMKFEMGTMPSLRK

LKLGFDVHIADQHDIIPVGIEHLSGLEEISAKIRVACTAHDHCRRFAESALTNAFMMHPG

RPSVNIRCVDWTFHDKDNDCVGTREEECRTPMKQEHFVKEDLSEKSAVLQNEHDEEAHKF

VDRRYYSIMDAAEIRRCPWSINEEQEQPVLIYDARTKISQSSSMHGEFWAAVQRLTGPAA

TPAKTKRHLHLTTSPELEDGFLPVRSLVFPSAPDPRCNMKKKKMRAGLEGVEQCGRTGPP

NRRGPNPGGLQSLSSATKPCDEMMRTETPNSYCKASQCLAACRVAVDRPGAAVGCCCKPE

DSCNC*

>BAR_RG021

MLAAAAAPPSSSLSDRRKYLCISCVSQVACEQQNTPILVHGVRCGECFLGGHHGEAVHGA

GEPVQQAQGPRARNQLPPARVPHDCRRHGRPAPFYGQDGLGKTTLAKAVYDGPHAKDKFC

LRAWITADGSPETSNWMREILRDVLQQVRPGDAMDVDGQHLEASLKEYLKDKRYLIIIDD

IRMDQWRIISSAFENNGTGSRILLTTTIQSVANRSPELENHSESLLGKCDGLPLALVSVS

DYLKSSTEATGELCAKLCRDLGSHLTGNHGHDNFSELRKVLLGNYDSFSGSALSCLLYLG

IFPNNHPLRKKVLIRRWLAEGYARSDDPWRSEEDTADDNFSNLIDQNIIQPVDTRNNSEV

KTCKTHGIMHEFLLNRSLAKRFITTSPHDPRVGINTTNSRHLSVDAAKQTKCMASDEELS

RVRSLTIFGDAGDAISYLHKCNLIRVLDLQECSDLNDNHLKRICVLSPWHLKYLNLGDNI

SELPRSIEGLHCLETLDLRETEIKFLPIEGIMLPHLAHLFGKFMLHKDDLNNVKKISKLL

KLFSSNKSNLQTLAGFITDGRKGFLQLIGHMKKLRKVKIWCRHVEGSSNYIADLSKAIQE

FTKAPIDMDRVRSLSIDSKEYCEKFLSSLDLEPCSEYSKYHLSSLKIHGKLLRLPPFFTS

LSGLIDLCITPATLTQDHLSALINLNRLLYLKLIADKLENLEIKHEALLSLRRLCFVVKS

VALAQPKIEQGALPNLVSLQLLCQGLVGLSGIEIRHLKNLKEVTIDSGVTAQTRQDWEQA

AKNHPNRPRVLLLGKVDPVESEEPGRPCAIRGRGKSSIGQESSEDGSDSSLKRMRLAEPS

SSSQLQVTGHQHPVVVAATEAASQPSMANL*

>BAR_RG022

MEEEVLSTCGLWGATPSLPGRLEGLLRRHGNILPNGAEDELPLIKQDVEKIISIILHGYS

KPKLEDNAMVVRCWMKEVRELSYDIEDCIDHYEHAMANSRSGFNDNIRRRKFNRRHGNKL

PPWVQEKLKQRLWMANKMREFSTRAQEALQRHAMYSNHGVATTAAIASTNTCGIDVSSSS

SLRPVRCEERAEDVLVGVDSAMNKLEDYLSGHAGEEKLRVLSIVGFGGIGKTTLANELYS

KIGRQFECRAFVRASQKPDMRRILTSILSQLRPHQPPDHRKVHSLISSIRAHLQDKRYMV

VIDNLWDISTWDIMRCALPDGNNCSRILTTTEIGDLALQSCGYDSKYVLTMKPLGEEDSR

KLFFSTIFGPYPKCPPGLSEVSYDIIRKCGGFPLAIVTIANLLSSRLGKPKEWDYVNKSL

CYSLVTNPTLEGMKQVLNLSYNSLPLHLKACLLYLTIYQEDYLIWKDDLVKQWIAEGFIC

TTEEQDKEEISGSYFDELLNRRMIQPVHINDNGEVISCVVHHMVMKFITYKSMEENFVIS

IDHSQTATKFADKVRRLSLDAGLTEDVMQPINMRLSQVRTLAFWGTSKFMPSIVDFRLLQ

VLILHLWDDHDNISLDLTRIAELFRLKYLKVTSNVILELQTQIQGLEFLETLQIDARVNA

VPSDVVQLRRLLHLSFPAETNLPNGIGQMTSLHTLGNFDLSGSSIENVQSLGELTNLQDL

RLTCSTVQTDNLKNKLQLFLGSVLWKLSNLKSMILVSTGPFHEIPLDEPGDTNSANDTGA

TTTSVTISSDHGLSSVSSPHTLLQRLEFLPHSFIFSYLPKWISQLNKLCILKIGVSELVR

NDVCVLCGLSVLAVLSLYIHNKPAERIIIGRTGFLALKYLKFKCRVPWLKFEANAMPILR

KLKLCFNVYEADQHGTIPDGIEHLSGLREVSAKIGLSGTADDLDKRSVESALNDAIKMHP

GHPRVNIQCVEWVFNGKEDNSSGSLQKQYNTRKRGSKEQRVIHKSQSKKRVLQNDSNEGS

RERVDKRYGDLSSVYISLHIPLRTYSVNPQERDWRGLIPHLKREEISISESSSSDESIIS

SFSNIETQFDLSKSSVNMPGMDGLVKILYPRSIDERFELSKSSVNVPRMDAHVKTPHPRS

IAKRSDISKSSINLPGMDDHAKIPHPRSVTERQRRRTISNKIRKLQDLVPNMDKQTSTAD

MLDTAIHYIEELRSRNEPALLPVSDWSMWRSKFSSAMVNARLFGVGYISSELLD*

>BAR_RG023

MASAAASAFLEAVMGKLFMVLDKEYNKHKALEQEISSLQQEFRMVAAAMDDQLLSMGRSD

ARTAVARLHAEEMLDLEHDIEDCVDRFMHLLTCKHNSLSGRTSLVCRVKHEVKKVQSRSS

FSEEIQKLRRCLSEAQQRVININPNPPSGCKPAESRSSTSSSTPCRAACSPVGIGEPMEE

LLSLLDEVEGEPEQVRVISIVGFGGLGKTTLAKAVYDSPRAKDRFSHRAWVTAGGSPETT

DWMREILRDVLQQVRPDNAMDVDAQHLEASLREYLTDKRYLIVIDDIDVDQLRIIESIFP

DNGTGSRIIVTTDNQQVANTCSHGNGYVYQMKTLGKEDSKKLAFSGLRSVEPGQGPASLL

AKCDGLPLALVSVSDYLKSSSEPTGELCAELCLNLGSDLKDDGHYSFAQLRKVLLDNYDS

FSGYTLSCLLYLGIFPNNRPLKKKVVIRRWLAEGYARSDDPRRSEEYTADKNFRKLIDRN

IIQPVNTRNNSEVKTCKTHGIMHEFLLNKSLAQRFIGTSLHDHPRVGINTTNARHLSVDA

AKQTECVASDEELSRVRSLTIFGDAGDTISCLRKCKLLRVLDLQECNDLNDDHLKHICEL

WHLKYLSLGGNINELPRSIQGLHCLETLDLRRTEIKFLPIEAIMLPHLAHLFGKFMLNKD

DLNNAKKMSKLQKVFSSNKSNLKTLAGFITDERKEFLQLIGHMKKLRKVKIWCKHVEGSN

NYIADLSKAIQEFTKAPIDMDRVRFLSLDSEECSENFLSSIHLEPCSEDFKYTLKSLKLH

GNLLQLPLFVTLLSGLIELCISSAILTQEHLSALVNLNSLLYLKLVADKLENFEIKQGAF

LSLRRLCFVVKNAASASPKFEQGAMPNLVSLQLLCQGLVGLSGIEIRHLKHLKEVTIDSR

VTAQTRQDWEQAAKNHPNRPRVLLLGEVHSVESEEPGRPMEKRRICVGQASSEDERDSSL

KRMRLSDPSSSRLQVIGHPHPVVVTATEAASQPSMAN*

>BAR_RG024

MEGAMFNLPGRLEELLCHHGSMLPKGADEEIPLIKQDLEEIISILHGHSEPKLEDHGMMV

RCWMKEVRELSYDIEDSIDQYEHATRSRNRPNIRHRKFNRWRGNKIPCFPQKLKQRLWMA

NKIREFSLRAQEALQRHAMYNNLGGVASTASTTRGDVCSATPLHPTQMQFREHVDNVRSV

SIEADGMEAALNDLNKLKNLLAGIPTASLVQFREHADKVRGIHTNIETILNKLENIPPGI

TTTTTTRGDVSSTSSRQPTRFMESTCHVGIDAAMDQLENLIDVCGEEKLKVVSIVGVGGV

GKTTLANKLYHKLRWQFECWAFVRSSQKTDMRRLLINILSQVWPHQSPDNWKVHTLISSI

RTHLQDKRYLIIIDDLWATSTWDIIKCALPDGNNSCRILTTTEIEDLALQSCSYDLKFIF

KMKPFGEDDSRKLFFSSIFGSHSKCPPEVSETSYDIVRKCGGLPLAIVIVASLVASHLEK

QEQWDYINKSLGYSLMANPTLEGMKQLLNLCYNNLPQHLKACMLYLSMYQDHIIWKDDLV

SQWIAEGFICAIEGHDEEEISRAYFDELLGRKIIQPVHIDDNGEVLSCVIHHVVLNFVTY

KTIEENFTIVIDHSQTTIRFADKIRRLSVHFGKVEDSTPPTNMRLSQVRSVAYFGVLKYM

PFIMEFRLIKVLVLHFLGDEDSTGIFDLTKISELVRLRYLKVTSNVTLKLPTRMQGLQYL

ETLKIDGKISAVPSDIIHLPGLLHLTLPAKTNLPNGIVHMTSLRTIGYFDLSCNSAENLW

SLGELTNLRDLQLTCSEIHSDNLKNNMKYLGSILGKLHNLKSMTLSPVGSSYADTLHIDR

ATSTGISVIGCSSVSSPPALLQRFDLLPCVCIFSNLPNWIGQLGNLCILKIGIRKITSDS

IDVLGVLPELTVLSLYVHTKLEERIIFKKAGFSILKYFKFKFRCSLVWMKFEAGALPNLR

KLKLGFDVHREDRYDTVPFGIEHLSRLEEISAKIRIDCTADNLSRRFAESSFANAIRMHT

GCPSINVRCVDWTSDCKDDDNVGTGEEEHRTLEKHHHIVKEGSNETTAVLQEDTWERAHK

SVNNRKTQPTERTKVRMSSMVDDGFSWRKYGQKQIQGDMHPRSYYRCAYSRDNHCPAKKQ

VQPTKDDPTVVDVVYYGEHTCGYTAHSRPTGSSNLQEEPQQPGLEQIPATSPYYDWIDLR

FIIPPSNKKVEKTKRKRVQVRVSSVEDVSQEYDGYSWRKYGQKDILGAKYPRGYYMCSHR

NTQGCKATKQVHRTDGDPLLFDVVYLGVHTCSDHIASLNEQVLLSDLSSASPQQSQINLQ

LSELDHSSATVTCTASSSVVVDDQDHQVVTSAAASFLSVDDMLDLDEDGEVWTTTTDEAT

AWDDGESIGEVSDMDFL*

>BAR_RG027

MESAVASALLKSVMGRLFTVLEKEYSKHRELAQETNSLQQDLRIVAAAMDDQLLSMGRSD

ARTAVARLQSKEMLDLAHDIEDYVDRFIHHLTCRQQCASAGRNSLVDRVAHELKKVQSRS

SFANEIQKLKRRLRQVHQDVIKNNPLAVTTSSGGQSSSPSPQDRHIADNPVGIEEPVEEL

LSLLDEVEGEPERMRVISIVGFGGLGKTTLAKAVYDSPRVKEKFHLRAWVPAGASPETSS

GMRGILRAVIQKILPNDALDVDGQLETSLKKYLKDKRYLIVIDDIGMDQWSIISSTFKDN

GTSSRIILTTTIQSIANSCSHGNGYVHQMNTLGEEDCKEIALPTGIRSPELETGSVPLLG

KCDGLPLALVSVSDYLKSSCEPTGELCANLCRNLGAHLKEQDGHPSFSELRKVLLDNYDS

LSGYALSCLLYLGIFPSNRPLKKKVVIRRWLAEGYARSDSLRNEEDIADENFNKLIDRNI

ILPVDTRNNSDVKTCKTHGIMHEFLLNMSLAQRFIMTLSRDHPRLISNARHLSVHDGELT

GYVASDEEFSRVRSLTVFGDASDTVSYVRKCKLIRVLDLQECNDFADDHLKHICKLWHLK

YLSFGYNINVLPRSIEGLHCLETLDLRRTKVKFLPIEAVMLPHLAHLFGKFMLHKDDLKN

VNKMSKLNPCKKQKKGMNILPKFFTSKKSNLQTLAGFITGENEGFLKLMGHMKKLRKVKI

WCKHVAGSSNYIADLSQAIQEFTKVPIDSDSNRSLSLYSGECSEDFLSALHLEPCSEDFK

YHVRSLKLQGRFLRLTPFVTSLSGLTEIFISSATLTQDHLSALITLNRLLYLKLIADKLE

NFEIKHGAFPSLRRLCFVVKSVTSDLPTIKQGALPNLVSLHLLCRGLVGLSGIEIRHLKH

LKEVVIYSDVTPQTKQDWARAAKNHPNRPKFSWPRKVDLVESEEPAKHLETEKRKYCSND

ELDYNLQEMRLSESRDHKRQKIGEGDTSKSSVGLVYPMYGDVGTDRTQVHLSNEETRRYD

RTEVDQKCPEMLQECKDKCSMVVDVDLRSDEQVNPPHPKLKNLMPGKEYDRQELIPTEGA

KVGQCQSGGAEDQIVHNTNCKKVVAQANHVFEQEDQGSQWHALSPQQDIASFDDWWASAE

VRVDGQEEENSPCVAPLNKAAPARCRADEGCRRSAVEGPASRRFDGEISPNLTTMAQSLY

GPNASLKSLNPSDIAHLRFTGVIFKS*

>BAR_RG030

MEGAVFNLPGRLDELLRRHRSILPKGAEDEIPLIKQDLEEIISILHGHCSEPKLEDHAMV

VKCWMKEVRELSYDIEDCIDQYEEFIEQYEHVAAARYYVRRRKFNRRHGNKLPPWVPEKL

KQRLWMANKIREFSLRVQEVIQRYTMYKNDLTGIASTASTTTTTIISDVSSSSSSHPAPG

GKCGYVGIDAATNKIEDWLTDGEHKKLKVVSIVGVGGVGKTTLANELYRKLGHQFEYRAF

VRSSHKPDMRSILISMLSQIHPQQPPDNWKVHNLISSIRTHLKDKRYLIIIDDLWSTSTW

DIISCTLPDGNSCSRILTTTEIEDLALQSCSYDSKYIFEMKPLGEEDSRNLFFSTVFGSR

PTCPPELSEASYDIVKKCGGLPLAVVTIASLLASQLEKQEQWDYINKTLGYSLMANPNLE

GMKQLLSLCYNSLPQHLKACMLYFSTYQEDTIIWKDDLVNQWIAEGFICAIEGHDKEEIS

RAYFDELVDKKIIQPVHINDNGEVLSCVVHYMVLNLITNTSIEENFIIAIDHSQATTRLA

DKVRRLSIHFSNVEDATPPTNMRLSQVRTLAFSGVLKCMSFITGFRLLRVLILHIWGDED

SISVNLNKISELVRLRYLKVTSNVTLELPTQMQGLQYLETMKIDGKIGAVPSDIIYLPGL

LHLSLPAKTNLPNGIFHLISLRTLGYFDLGCNSIENMQSLGELTNLQDLQLTYSTAHSDD

LKNYMQCLGSILEKLKSLKSITLSRADSSDANTLHIESAISMRISVDGWSNLSSPPALLQ

RIELLPCVCIFSSIPNWIGKLGNLCILKIGIREVTRSDVDVLGRLPALTVLSLYVHRKPM

ERIIFDNVGFSILKYFKFRCIVAWMKFEAGAMPNLQKLKLGFDVRRADQHGTIPVGIKHL

SGLKEISAKIRVACTVDDLCKGFAESELTNAIRMHPGRPRVNIRCIDWTFDGKDDNNVGT

REEESTTFEKQHHIVKVDSTVKFAVPEKDPGREADKSIDRRYGFLTLKIPYPLINRTFLV

YYDEYCFYYEYFC*

>BAR_RG031

MESAAASALLKSVMGRLFMALEKEYSKHKGLAQETHSLQQDLRMIAAAMDDQLRVLGRND

ARNAVARLHTEEMLDLSHDIEDCVDRFLHRLTCRQQRGSGSNGAGPRASSMVRRMAHEFS

KVQSRSSFADEIQKLKRRIREAHQRVMDIKSIVDVIAGGQPTTGAMSSSATAPCRNTCNP

VGIGEPVEELLSLLDEVEGEPEQMRVISVVGFGGLGKTTLARAVYDSPGAKGKFSHRAWV

TAGSSPESSSRILHALLQQVLPKDAIGVDGQHDLEALLKEYLKDKRYLIVMDDINMEQWS

IIRSTFEDNGTSSRIILTTTIQSVANMCSHGNGYVYKMNTLGEEDSKTLAFPGFRSPELE

QGSESLLGKCDGLPLALVSVSDYLKSSSEPTGELCAKLCRNLGSHLKERDGHYSFSELRK

VLLDSYDSFSGYALSCLLYLGIFPNNRPLKKKVVIRRWLAEGYARSDSLRSEEDIADENF

SKLIDQNIVQPVDTRNNSEVKTCKTHGIMHEFLLNKSLSQIFIAKSSRDHPRLGIDTNAR

HLSVHAGELTESVESDEELSRVRSLTIFGDAGDAICYVRKCKLIRVLDLQECNDLDDEHL

KYICKLWHLKYLSFGSNISELPRSIEGLHCLETLDLRRTKINFLPTEAIMLPHLAHLFGK

FMLHRDDVNSVNKMTKLQKFFSSKKSNLQTLAGFITDESKGFLQHIGHMKKLRKVKIWFK

HVPGSSNYIADLSQAIQEFTKAPIDRDIDRSLSLDSEECPENFLSSLDLETCSEGSKYAL

RSLKLNGELHRLPPFVTLLSGLTELCISSATLTQDHLSALINLNRLLYLKLVAYKLVNIE

IKHGEFPSLRRLCFVVKSVNSALPTIEHGALPNLISLQLLCQGLVGLSGIEIKHMKYLKE

VTINSGVAIQWEQAAKKHPNRPKILILRKVNPMESEEPERPCAIREQRKISVAQTTSLDD

RLDSSLNKMRLSKPSSSRLQVFVHPVVITATEAAPQSSLANL*

>BAR_RG032

MARRSETARSQDESRSGGRGRTQGTEPSSDQSWPGQPTTNGRKIGGYGGRRRRGWEDWGT

EKNTTTLITLGSRRESSTQRKRGSAEPPIEARVGMEAAAMAVTAATGALEPVLLKLAALL

DDGECNLLEGSRSDAEFIRSELEAVHSLLTPNILGRMGDDDAACKDGLIAEVRELSYDLD

DAVDDFLELNFEQRRSTSPFGELKARVEEHVSNRFSDWKLPATSLRPLSVNRRAGLLPPD

GDLVGMGKRKEELIELLEQGSSDASRWRKRKPHVPLRIIRGEMQQIVIKVAILDERNRSK

AMTLVAKTGGVCSIAIVGDPRDKVLVVGDGIDPIKLTSALRKKVGHAELLQVSQANKDVK

ETTPMLAPVKSICEFHEVKTVCILGLPGGGKTRVARVLYHTLGTQFQCRVFTSVSPSSSS

SPSPNLTETLADIFAQAQLGVTDTPSTPYGGSGTGRALQQHLIDNISAFLLNKKYLIVID

DIWHWEEWEVIRKSIPKNDLGGRIIMTTRLNSIAEKCHSDDNDVFVYEVGDLDNNDALSL

SWGIATKSGAGNKIGTGEDNPCYDIVNMCYGMPLALIWLSSALAGEIEELGGAEVKKCRD

LRHIEDGILDIPSLQPLAESLCLGYNHLPLYLRTLLLYCSAYHWSNRIERGRLVRRWIAE

GFVSEEKEAEGYFGELIDRGWITQHGDNNSYNYYEIHPVMLAFLRCKSKEYNFLTCLGLG

SDTSTSASSPRLIRRLSLQGGYPVDCLSSMSMDVSHTCSLVVLGDVAGIPFNMFKRLRVL

DLEDNKDIQDSHLQGICEQLSLRVRYLGLKGTRIRKLPQEMRKLKHLEILYVGSTRISEL

PQEIGELKHLRILDVRNTDITELPLQIRELQHLHTLDVRNTPISELPPQVGKLQNLKIMC

VRSTGVRELPKEIGELKHLQTLDMRNTSMRELPWQAGQISQSLRVLAGDSGDGVRLPEGV

CEALINAIPGAARAKCREVLSIAIIDRFGPPLVGIFKVPGSHMRIPKMIKDHFRVLSCLD

IRLCHKLEDDDQKFLAEMPNLQTLVLRFEALLRQPITIKDTGFQMLESFRVDSRVPRITF

HEDAMPNLKLLEFKFYAGPPSDNPVCITNLKSLQKVVFRCSPWYKSDAPGISATVDVVKK

EAEEHPNRPITLLINAGYKEIPIESHGSSENIAGSSGTSGIDTEPAQAQHDNLPAVRDDY

KGKGILLDGRCPTCGRATKIEEETQDRVADIEIQTETTS*

>BAR_RG033

MELVVGASEATMKSLLGKLGNLLAQEYALISGIRGDIQYINDELASMQAFLRDLSNVPEG

HSHGHRMKDWMKQIRDIAYDVEDCIDDFAHRLPQDSISDAKWSFLLTKIYELWTWWPRRV

IASNIAQLKVRAQQIADRRSRYGVNNPEHLDSSSSARTRAVNYEIAEYQVTSPQIIGIKE

PVGMKTVMEELEVWLTNPQAENGQAVLSIVGFGGVGKTTIATALYRKVSDKFQCRASVAV

SQNYDQGKVLNSILSQVSNQEQGSSTTVSEKKNLTSGAKSMLKTALSLLRGNCICQPEND

GNPDNTPIRLQETTDDDQNPRKLEQLLAEKSYILLIDDIWSAETWESIRSILPKNNKGGR

IIVTTRFQAVGSTCSPLETDRLHTVDFLTDDESQNLFNTSICESKIRKDSNKVDEQVPEE

IWKICGGLPLAIVTMAGLVACNPRKACCDWSKLCKSLFPEQETPLTLDGVTRILDCCYND

LPADLKTCLLYLSIFPKGWKISRKRLSRRWIAEGFANEKQGLTQERVAEAYFNQLARRNL

VRPMDHGSNGKVKTFQVHDMVLEYIMSKSIEENFITVVGGHWQMTAPSNKVRRLSMQSSG

SNHGSSTKGLNLAQVRSLTVFGNLNHVPFHSFNYGIIQVLDLEDWKGLKERHMTEICQML

LLKYLSIRRTEISKIPSKIQKLEYLETLDIRETYVRELPKSIVQLKRISSILGGNKNIRK

GLRLPQEKSKKPIKNPLPQGKTKKPTKKGFLSQEKGKGAMKALRVLSGIEIVEESSEVAA

GLHQLTGLRKLAIYKLNITNGGDTFKQLQSSIEYLGSCGLQTLAINDENSEFINSLGDMP

APPRYLVALELSGKLEKLPKWITSITTLNKLTISVTVLRTETLEILRILPSLFSLTFAFS

LSAAKQDQDIIKDILENNKWDSDGEIVIPAEGFKSLKLLRFFAPLVPKLSFLDKNAMPAL

EIIEMRFKDFEGLFGIEILENLREVHLKVSDGAEAITKFLVNDLKDNTEKPKVFVDGIVT

A*

>BAR_RLK003

MALVRLPVWIFVAALLIASSSTVPCASSPGPTASKSNSSDTDLAALLAFKAQLSDPNNIL

AGNWTTGTPFCRWVGVSCSSHRRRRQRAAALELPNVPLQGELSSHLGNISFLFILNLTNT

GLTGSVPNEIGRLRRLELLDLSHNAMSGGIPIAIGNLTRLQLLNLQFNQLYGPIPAELQG

LHSLGSMNLRHNYLTGSIPDDLFNNTPLLTYLNVGNNSLSGLIPGCIGTLPILQHLNLQA

NNLTRAVPPAIFNMSKLSTISLISNGLTGPIPGNTSFSLPVLRWFAISKNNFFGQIPVGL

AACPYLQVIAMPYNLFEGVLPPWLGRLTNLDAISLGGNNFDAGPIPTELSNLTMLIVLDL

MTCNLTGNIPADIGHLGQLSWLHLAMNQLTGPIPDSLGNLSSLAILLLKGNLLDGSLPST

VDSMNSLTAVDVTENNLHGDLNFLSTVSNCRKLSTLQMDLNYITGILPDYVGNLSSQLKW

FTLSNNKLTGTLPATISNLTALEVIDLSHNQLRNAIPESIMTIENLQWLDLSGNSLSGFI

PTNTALLRNIVKLFLESNEISGSIPKDMRNLTNLEHLLLSDNKLMSTIPPSLFHLDKIVR

LDLSRNFLSGALPVDIGYLKQITIMDLSDNHFSGRIPYSIGQLQMLTHLNLSANGFYDSV

PDSFGNLTGLQTLDISHNSISGTIPNYLANFTTLDSLNLSFNKLHGQIPEGGVFANITLQ

YLEGNSGLCAAARLGFPPCQTTSPNRNNGHMLKYLLPTIIIVVGVVACCLYVMIRKKANH

QNTSAGKADLISHQLLSYHELLRATDDFSDDNMLGFGSFGKVFRGQLSNGMVVAIKVIHQ

HLEHAMRSFDTECRVLRMARHRNLIKILNTCSNLDFRALVLQYMPKGSLEALLHSEQGKQ

LGFLERLDIMLDVSMAMEYLHHEHYEVVLHCDLKPSNVLFDDDMTAHVADFGIARLLLGD

DNSMISASMPGTVGYMAPEYGTLGKASRKSDVFSYGIMLLEVFTAKRPTDAMFVGELNIR

QWVQQAFPAELVHVVDCQLLQDGSSSSSNTHDFLVPVFELGLFCSADSPEQRMAMSDVVV

TLKKIRKDYVKLMATTVSVVQQ*

>BAR_RLK004

MAWLLLPPFNSIRLLMLMLVHSLTIPYASGSIPRDGGSSSNGTGVDLSALLAFKARLSDP

LGVLAGNWTTKVSMCRWVGVSCSRRRPRVVGLKLWDVPLQGELTPHLGNLSFLRVLNLSG

VNLTGPIPPDLGRLQRLRILRLADNTISDAIPSALGNLTKLEILNLYGNHISGNIPAELQ

NLHSLRQMVLTRNYLSGPVPEYLFNATPSLTHIYLGYNSLSGSIPYCVGSLPMLRVLALP

DNQLSGPVPPAIFNISSLEVIGVPENNLTGPIPTNRSFNLPMLQYIELDTNNFTGLISSG

LASCQKLEIISLSENLFSGVVPQWLAKMSRLTFLFLDGNELVGTIPSLLGNLSMLSGLDL

SDSNLTGHIPVELGTLTKLTYLYLSLNQLIGTFPAFIGNLSELSYLGLGYNQLTGPVPST

FGNIRSLIEIKIGGNHLQGDLSFLSSLCNCRQLQYLLISHNSFTGSLPNYVGNLSTELLG

FEGDDNHLTGGLSATLSNLTNLRALNLSYNQLSDSIPASLMKLENLQGLDLTSNGISGPI

PEEIGTARFVWLYLTDNKLSGSIPDSIGNLTMLEHISLSDNKLSSTIPTSLFYLSIVQLF

LSNNTLTGTLPSDLSHIQDMYALDTSDNLLVGQLPNSFGYHQMLAYLNLSHNSFTDSIPN

SISHLTSLEVLDLSYNNLSGTIPKYLANFTYLTTLNLSSNKLKGEIPNGGVFSNITLISL

MGNAALCVLPRLGFSPCLNKSDSTNGSHYLKFILPAITMAVGALALCLYQMTRKRIKREL

DIAEPTSFRLVSYQEIVRATESFNEDNMLGTGSFGKVYKGHLDDGMVVAVKVLNMQEEQA

IRSFDVECQVLRMVRHRNLIRILNICSNLEFRALLLQYMPNGSLETYLHKEGHPPLGFLK

RLDIMLDVSMAMEHLHYHHSEVVLHCDLKPSNVLFDEEITAHVADFGIAKLLLGDDNSAV

SASMPGTIGYMAPEYAFMGKASRKSDVFSYGIMLLEVFTGKRPTDAMFVGDVSLRKWVSE

AFPARIADIVDGRLLQAEALIEQGVRQNNATSLPRSATWPNEGLLLPIFELGLMCCSSSP

AERMEINDVVVKLKSIRKDYFACTGAI*

>BAR_RLK006

MADEPSNGTDIAALLAFKAQVSDPLGFLRDAWREDNASCFCRWIGVSCSRRRQRVTALEL

PGIPLQGSITPHLGNLSFLYVLNLTNTSLTGTLPGVIGRLHRLELLDLGYNALSGNIPAT

IGNLTKLELLNLQFNQLSGPIPAELQGLRSLGSMNLRRNYLSGSIPNTLFNNTPLLGYLS

IGNNSLSGPIPHVIFSLHVLQVLVLEHNQLSGSLPPAIFNMSRLEKLYATRNNLTGPIPH

PAGNQTLISIPMIRVMCLSFNGFTGRIPPGLAACRKLQMLELGGNLLTDHVPEWLAGLSL

LSTLVIGQNELVGSIPVVLSNLTKLTVLDLSSCKLSGMIPLELGKMTQLNILHLSFNRLT

GPFPTSLGNLTKLSFLGLESNLLTGQVPETLGNLRSLYSLGIGKNHLQGKLHFFALLSNC

RELQFLDIGMNSFSGSISASLLANLSNNLQYFYANNNNLTGSIPATISNLTNLNVIGLFD

NQISGTIPDSIMLMDNLQALDLSINNLFGPIPGQIGTPKGMVALSLSGNNLSSSIPNGVG

NLSTLQYLFLSYNRLSLVIPASLVNLSNLLQLDISNNNLTGSLPSDLSSFKVIGLMDISA

NNLVGSLPTSLGQLQLSSYLNLSQNTFNDSIPDSFKGLINLETLDLSHNNLSGGIPKYFS

NLTYLTSLNLSFNNLQGQIPSGGIFSNITMQSLMGNAGLCGAPRLGFPACLEKSDSTRTK

HLVKIVLPTVIAAFGAIVVFLYLMIAKKMKNPDITASFGIADAICHRLVSYQEIVRATEN

FNEDNLLGVGSFGKVFKGRLDDGLVVAIKILNMQVERAIRSFDAECHVLRMARHRNLIKI

LNTCSNLDFRALLLQFMPSGNLESYLHSESRPCVGSFLKRMEIMLDVSMAMEYLHHEHHE

VVLHCDLKPSNVLFDEEMTAHVADFGIAKMLLGDDNSAVSASMPGTIGYMAPEYAFMGKA

SRKSDVFSFGIMLLEVFTGKRPTDPMFIGGLTLRLWVSQSFPENLIDVADEHLLLDEETR

LCFDHQNTSLGSSSTGRSNSFLMSIFELGLLCSSESPEQRMAMNDVVSKLKGIKKDYSAS

MLEMQRPRQY*

>BAR_RLK008

MALVRLPVWIFVAALLIASSSTVPCASSLGPIASKSNGSDTDLAALLAFKAQLSDPNNIL

ATAGSRRRQRVTALKLPNVPLQGELSSHLGNISFLFILNLTNTGLAGSVPNEIGRLHRLE

LLDLGHNAMSGGIPIAIGNLTRLQLLNLQFNQLYGPIPEELQGLHSLGSMNLRHNYLTGS

IPDDLFNNTPLLTYLNVGNNSLSGLIPGCIGSLPILQHLNLQANNLTGAVPPAIFNMSKL

STISLISNGLTGPIPGNTSFSLPVLRWFAISKNNFFGQIPVGLAACPYLQVIAMPYNLFE

GVLPPWLGRLTNLDAISLGGNNFDAGPIPTELSNLTMLTVLDLTTCNLTGNIPADIGHLG

QLSWLHLAMNQLTGPIPASLGNLSSLAILLLKGNLLDGSLPSTVDSMNSLTAVDVTENNL

HRDLNFLSTVSNCRKLSTLQMDLNYITGILPDYVGNLSSQLKWFTLSNNKLTGTLPATIS

NLTALEVIDLSHNQLRNAIPESIMTIENLQWLELSGNSLSGFIPSNTALLRNIVKLFLES

NEISGSIPKDMRNLTNLEHLLLSDNQLTSTIPPSLFHLDKIVRLDLSRNFLSGALPVDVG

YLKQITIMDLSDNHFSGRIPYSIGQLQMLTHLNLSANGFYDSVPDSFGNLTGLQTLDISH

NSISGTIPNYLANFTTLVSLNLSFNKLHGQIPEEGVFANITLQYLEGNSGLCGAARLGFP

PCQTTSPNRNNGNMLKYLLPTIIIVVGVVACCLYVVIRKKANHQNTSAGKADLISHQLLS

YHELLRATDDFSDDSMLGFGSFGKVFRGQLSNGMVVAIKVIHQHLEHAMRSFDTECHVLR

MARHRNLTCSNLDFRALVLQYMPKGSLEALLHSEQGKQLSFLERLDIMLDVSMAMEYLHH

EHYEVVLHCDLKPSNVLFDDDMTAHVADFGIARLLLGDDNSMISASMPGTVGYMAPEYGT

LGKASRKSDVFSYGIMLLEVFTAKRPTDAMFVGELNIRQWVQQAFPAELVHVVDCQLLQD

GSSSSSNTHDFLVPVFELGLLCSADSPEQRMAMSDVVVTLKKIRKDYVKLMATTVSVVQQ*

>BAR_RLK010

MALGLLVWMYIVLLIALSTVSAASPPGPSKSNGSETDLAALLAFKAQLSDPLGILGSNWT

VGTPFCRWVGVSCSHHRQRVTALDLRDTPLLGELSPRLGNLSFLSILNLTNTGLTGSVPD

DIGRLHRLEILELGYNTLSGSIPATIGNLTRLQVLDLQFNSLSGPIPADLQNLQNLSSIN

LRRNYLTGLIPNNLFNNTHLLTYLNIGNNSLSGPIPDCIGSLPILQTLVLQVNNLTGPVP

PAIFNMSMLRALALGLNGLTGPLPGNASFNLPALQWFSITRNDFTGPIPVGLAACQYLQV

LGLPDNLFQGAFPPWLGKLTNLNIISLGGNQLDAGPIPAAVGNLTMLSVLDLASCNLTGP

IPADIRHLGQLSELHLSMNQLTGPIPASIGNLSALSYLLLMGNMLDGLVPATVGNINSLR

GLNIAENHLQGDLEFLSTVSNCRKLSFLRVDSNYFTGNLPDYVGNLSSTLQSFVVAGNKL

GGEIPSTISNLTGLMVLALSDNQFHSTIPESIMEMVNLRWLDLSGNSLAGSVPSNAGMLK

NAEKLFLQSNKLSGSIPKDMGNLTKLEHLVLSNNQLSSTVPPSIFHLSSLIQLDLSHNFF

SDVLPADIGNMKQINNIDLSTNRFTGSIPNSIGQLQMISYLNLSVNSFDDSIPDSFGELT

SLQTLDLSHNNISGTIPKYLANFTILISLNLSFNNLHGQIPKGGVFSNITLQSLVGNSGL

CGVARLGLPSCQTTSPKRNGRMLKYLLPAITIVVGAFAFSLYVVIRMKVKKHQKISSSMV

DMISNRLLSYHELVRATDNFSYDNMLGAGSFGKVYKGQLSSGLVVAIKVIHQHLEHAMRS

FDTECHVLRMARHRNLIKILNTCTNLDFRALVLEYMPNGSLEALLHSEGRMQLGFLERVD

IMLDVSMAMEYLHHEHHEVVLHCDLKPSNVLLDDDMTAHVSDFGIARLLLGDDSSMISAS

MPGTVGYMAPEYGALGKASRKSDVFSYGIMLLEVFTGKRPTDAMFVGELNIRQWVYQAFP

VELVHVLDTRLLQDCSSPSSGFLVPVFELGLLCSADSPEQRMAMSDVVVTLKKIRKDYVK

SISTTGSVALPAYTKE*

>BAR_RLK011

MAPGSPVCIIVSALLLITLSPVAAAAAASPGPSKSNGSDTDLAALLAFKGELSDPYNILA

TNWTAGTPFCRWMGITCSRRQRQRVTGVELPGVPLQGKISPHIGNLSFLSVLNLTITNLT

GSIPDDIGRLHRLELLDLGNNALSGVIPASIGNLTRLGVLRLAVNQLSGQIPADLQGLHS

LRNINIRNNGLTGSIPNSLFNNTPLLSYLNIANNSLSGSIPACIGSLPMLQFLDLQVNQL

AGPVPPGVFNMSMLGVIALALNGLTGPIPGNESFRLPSLWFFSIDANNFTGPIPQGFAVC

QQLQVFSLIQNLFEGALPSWLGKLTNLVKLNLGENHFDGGSIPDALSNITMLASLELSTC

NLTGTIPADIGKLGKLSDLLIARNQLRGPIPASLGNLSALSRLDLSTNLLDGSVPATVGS

MNSLTYFVIFENSLQGDLKFLSALSNCRKLSVLEIDSNYFTGNLPDYVGNLSSTLQAFIA

RRNNISGVLPSTVWNLTSLKYLDLSDNQLHSTISESIMDLEILQWLDLSENSLFGPIPSN

IGMLKNVQRLFLGTNQFSGSISMGISNMTKLEYLDLSDNQLASTVPPSLFHLDRLVKLDL

SHNFLSGALPADIGYLKQMNIMDLSSNHFTGILPDSIAQLQMIAYLNLSVNSFQNSIPDS

FRVLTSLETLDLSHNNISGTIPEYLANFTVLSSLNLSFNNLHGQIPETGVFSNITLESLV

GNSGLCGAVRLGFSPCQTTSPKKNHRIIKYLVPPIIITVGAVACCLYVILKYKVKHQKMS

VGMVDMARHQLLSYHELARATNDFSDDNMLGSGSFGKVFKGQLSSGLVVAIKVIHQHMEH

AIRSFDTECRVLRTARHRNLIKILNTCSNLDFRALVLEYMPNGSLEALLHSDQRIQLSFL

ERLDIMLDVSMAMEYLHHEHCEVVLHCDLKPSNVLFNDDMTAHVSDFGIARLLLGDDSSM

ISASMPGTVGYMAPEYGAIGKASRKSDVFSYGIMLLEVFTRRRPTDAMFAGELNIRQWVL

QAFPADLVHVVDGQLLQDSSSCTSSIDGFLMPVFELGLLCSADSPEQRMVMSDVVVMLKK

IRKDYMKSIATMGKAEHRPAVFH*
